# Continuation rates of two different-sized copper intrauterine devices among nulliparous women: Interim 12-month results of a single-blind, randomised, multicentre trial

**DOI:** 10.1016/j.eclinm.2022.101554

**Published:** 2022-07-16

**Authors:** David Hubacher, Courtney A. Schreiber, David K. Turok, Jeffrey T. Jensen, Mitchell D. Creinin, Kavita Nanda, Katharine O'Connell White, Ila Dayananda, Stephanie B. Teal, Pai-Lien Chen, Beatrice A. Chen, Alisa B. Goldberg, Jennifer L. Kerns, Clint Dart, Anita L. Nelson, Michael A. Thomas, David F. Archer, Jill E. Brown, Paula M. Castaño, Anne E. Burke, Bliss Kaneshiro, Diana L. Blithe

**Affiliations:** a FHI 360, Durham, NC, USA; b Department of Obstetrics and Gynecology, University of Pennsylvania, Philadelphia, PA, USA; c Department of Obstetrics and Gynecology, University of Utah, Salt Lake City, UT, USA; d Department of Obstetrics & Gynecology, Oregon Health & Science University, Portland, OR, USA; e Department of Obstetrics and Gynecology, University of California, Davis, Sacramento, CA, USA; f Department of Obstetrics and Gynecology, Boston University, Boston, MA, USA; g Planned Parenthood Greater New York, New York, NY, USA; h Department of Obstetrics and Gynecology, University Hospitals Medical Center and Case Western Reserve University, Cleveland, OH, USA; i Department of Obstetrics, Gynecology and Reproductive Sciences, University of Pittsburgh and Magee-Womens Research Institute, Pittsburgh, PA, USA; j Planned Parenthood League of Massachusetts, Boston, MA, USA; k Department of Obstetrics, Gynecology and Reproductive Sciences, Center for Global Reproductive Health, University of California, San Francisco, San Francisco, CA, USA; l Health Decisions, Durham, NC, USA; m Essential Access Health, Los Angeles, CA, USA; n Department of Obstetrics and Gynecology, University of Cincinnati, Cincinnati, OH, USA; o Department of Obstetrics and Gynecology, Eastern Virginia Medical School, Norfolk, VA, USA; p Contraceptive Development Program, Eunice Kennedy Shriver National Institute of Child Health and Human Development, National Institutes of Health, Bethesda, MD, USA; q Department of Obstetrics and Gynecology, Columbia University Irving Medical Center, New York, NY, USA; r Department of Gynecology and Obstetrics, Johns Hopkins University, Baltimore, MD, USA; s Department of Obstetrics, Gynecology, and Women's Health, University of Hawaii, Honolulu, HI, USA

**Keywords:** Copper intrauterine device, IUD, Nulliparous, Continuation rates, 12-Month, Early removal, Bleeding and pain, Expulsion, Comparative, Trial, Randomized, Blinded, Satisfaction

## Abstract

**Background:**

The most widely used copper intrauterine device (IUD) in the world (the TCu380A), and the only product available in many countries, causes side effects and early removals for many users. These problems are exacerbated in nulliparous women, who have smaller uterine cavities compared to parous women. We compared first-year continuation rates and reasons/probabilities for early removal of the TCu380A versus a smaller Belgian copper IUD among nulliparous users.

**Methods:**

This 12-month interim report is derived from a pre-planned interim analysis of a sub population and focused on key secondary comparative endpoints. In this participant-blinded trial at 16 centres in the USA, we randomised participants aged 17–40 in a 4:1 ratio to the NT380-Mini or the TCu380A. In the first year, participants had follow-up visits at 6-weeks and 3, 6, and 12-months, and a phone contact at 9 months; we documented continued use, expulsions, and reasons for removal. Among participants with successful IUD placement, we compared probabilities of IUD continuation and specific reasons for discontinuation using log-rank tests. This trial is registered with ClinicalTrials.gov number NCT03124160 and is closed to recruitment.

**Findings:**

Between June 1, 2017, and February 25, 2019, we assigned 927 nulliparous women to either the NT380-Mini (*n* = 744) or the TCu380A (*n* = 183); the analysis population was 732 (NT380-Mini) and 176 (TCu380A). Participants using the NT380-Mini, compared to the TCu380A, had higher 12-month continuation rates (78·7% [95% CI: 72·9–84·5%] vs. 70·2% [95% CI: 59·7–80·7], *p* = 0·014), lower rates of removal for bleeding and/or pain (8·1% vs. 16·2%, *p* = 0·003) and lower IUD expulsion rates (4·8% vs. 8·9%, *p* = 0·023), respectively.

**Interpretation:**

The NT380-Mini offers important benefits for a nulliparous population compared to the TCu380A in the first twelve months, when pivotal experiences typically occur. Higher continuation rates with the NT380-Mini may avert disruptions in contraceptive use and help users avoid unintended pregnancy.

**Funding:**

Bill & Melinda Gates Foundation, *Eunice Kennedy Shriver* National Institute of Child Health and Human Development, and Mona Lisa, N.V. (Belgium).

**Research in context**

**Evidence before this study**

We searched PubMed for articles up to Oct 1, 2021 using terms “(nulliparous) AND (IUD) AND (copper) AND (randomized) AND (trial)” We did not find any new evidence that wasn't previously included in several systematic reviews. Most previous trials involved only parous women and found similar clinical performance among different types of copper intrauterine device (IUDs). Of the trials involving/reporting only nulliparous women, only one included the TCu380A compared to a smaller product, yet the veracity of reporting is questionable. The remaining trials of nulliparous women involved products no longer used, or products of similar sizes, or trials with insufficient study sizes. In some countries, only a large product, such as the TCu380A, is available to serve the needs of both parous and nulliparous women. However, even in countries with alternative copper IUDs on the market, provision of larger products occurs among nulliparous women.

**Added value of this study**

Because the uterine cavity of nulliparous women is more compact than that of parous women, a smaller product could fit better and cause fewer side effects. This comparative trial among nulliparous women found important first-year advantages of a smaller copper IUD: higher overall continuation rates, fewer removals due to bleeding and/or pain, and fewer product expulsions from the uterine cavity.

**Implications of all the available evidence**

The results of this randomised trial provide scientific evidence that the dominant copper IUD used worldwide causes increased side effects and expulsion rates compared to the NT380-Mini in a nulliparous population. While these results do not categorically prove that all smaller products perform better, the findings make clear that an important population interested in preventing first births with copper IUDs, needs access to additional choices. Complete 3-year safety and efficacy results will be published when available.

Alt-text: Unlabelled box

## Introduction

Increased bleeding and pelvic pain are side effects that affect some copper intrauterine device (IUD) users, with first-year removal rates of 5% to 15% for these reasons.^1^^,^^2^ Nulliparous users have twice the rate of removal due to bleeding and pain compared to parous users.^2^ Disproportionately high removal rates for side effects may be due to numerous factors, including size and shape of the product and incompatibility with the dimensions of the uterine cavity.^3^ Numerous organizations support IUD use for nulliparous women and/or adolescents because the benefits far outweigh any serious health risks.^4^, ^5^, ^6^

Nulliparous women typically have smaller uteri than parous women. A systematic review of endometrial cavity dimensions (mean fundal width x mean cavity length) measured mechanically reported 25·1mm x 33·7mm (nulliparous) versus 34·9mm x 38·6mm (parous); imaging measurements were 28·2mm x 37·0mm (nulliparous) versus 32·1mm x 44·3mm (parous).^7^ Using 3-dimensional ultrasound, Wildemeersch et al.^8^ estimated that the mean fundal uterine width of nulliparous women was 21·6mm, with 75% of measurements falling between 18mm and 24·8mm. IUD embedment is associated with narrow uterine cavities.^9^

Globally, the TCu380A is the most widely used copper IUD and is the largest product in its class; the T-shaped plastic frame measures 32mm (horizontal arms) x 36mm (vertical stem) and has 380mm^2^ surface area of copper (the active pharmaceutical ingredient for contraception).^10^ It is the only copper IUD currently marketed in the USA. The product, developed in the 1970s, was sized according to the typical dimensions of parous uteri.^11^

Previous research suggests that smaller copper IUDs may have lower rates of expulsion and removals due to bleeding and pain compared to larger IUDs among nulliparous users,^12^ but clear evidence is lacking.^13^ Most prior trials had shortcomings: dissimilar comparative devices (very different shapes and/or varying amounts of copper) and small study sizes involving many products no longer used. One adequately powered study from Mexico conducted over 20 years ago (the last published comparative trial on the topic) demonstrated superiority of a smaller product,^14^ yet had numerous methodological flaws casting doubt on the findings.^15^ A comprehensive, systematic review of 34 comparative copper IUD trials spanning over 30 years could not provide sub-analyses on nulliparous women due to insufficient numbers; the authors could only conclude that in a predominately parous population, the TCu380A is the most effective product, with bleeding and pain removal rates similar to other products.^16^

Early copper IUD discontinuations are more concentrated in the pivotal first year, compared to later years.^2^ Users who experience early signs of intolerable or bothersome side effects are likely to seek product removal within a year of IUD placement.^17^ Nulliparous women trying a copper IUD for the first time may have limited options because better products or evidence of benefits are unavailable. A recent database study of the UK's National Health Service (NHS) reported high first-year discontinuations with larger copper products and found that 44% of patients receiving the standard (larger) copper IUD were nulliparous, despite having smaller products available in the NHS.^13^ Here we present first-year results of an ongoing randomised trial of two copper IUDs which differ in size and frame type, focusing on nulliparous participants. We compare secondary endpoints of continuation rates, reasons for early removal, and product satisfaction.

## Methods

### Study design and study oversight

We are conducting a three-year, phase III single (participant)-blind randomised trial at 16 US centres to measure contraceptive efficacy of the NT380-Mini (main goal of the program for regulatory filing and possible approval in the USA) and to compare secondary endpoints of the NT380-Mini and the TCu380A in a predominately nulliparous population. Recruitment occurred from June 2017 through February 2019. This 12-month interim report is derived from a pre-planned interim analysis of a sub population, per contractual agreement with funder, and focused on key secondary comparative endpoints. A central IRB and centre-specific IRBs (as required) approved the study; all participants gave written informed consent before any study procedures occurred. We provided annual continuing review documentation to the central IRB (Advarra protocol number Pro00020530) and centre IRBs. The trial is registered on Clinicaltrials.gov (NCT03124160).^18^

We used an adaptive trial design (monitoring interim results on safety, efficacy, and IUD expulsions, and taking action if necessary to end the trial early). An independent data and safety and monitoring board (DSMB) has conducted periodic reviews of this study. The DSMB did three interim assessments on pregnancy rates and IUD expulsion rates using cumulative 6-month, 9-month, and 12-month estimates. For each review, the DSMB used pre-specified thresholds on the lower bound of the 95% confidence intervals to identify possible concerns. If the lower bound on the 6-month pregnancy rate exceeded 1.0% and/or if the IUD expulsion rate exceeded 5.6% in either arm of the trial, the DSMB could have recommended changes in the conduct of the study. For the 9-month estimates, the lower bound thresholds were 1.2% and 7.1%, respectively. The equivalent 12-month thresholds were 1.4% and 8.4%, respectively.

### Procedures

The Mona Lisa® NT Cu380-Mini (henceforth NT380-Mini) is a Nova-T-framed plastic IUD measuring 24mm (horizontal arms) x 30 mm (vertical stem); the copper wire on the stem has 380 mm^2^ of surface area. It was first approved in Belgium in 2014 (Mona Lisa, N.V., Belgium) for use up to 5 years and is marketed in thirteen European countries and in Canada. The comparator product is the TCu380A (Paragard®, Cooper Surgical, Inc., Trumbull, CT); it is a Tatum-T-framed plastic IUD measuring 32 mm (horizontal arms) x 36mm (vertical stem) with copper on the arms and stem totalling 380 mm^2^ of surface area. This product was developed in the 1970s and approved by the US Food and Drug Administration (FDA) in 1984. The branded product has a 10-year indication for contraception in the USA; the generic TCu380A manufactured and distributed worldwide has a 12-year indication in some countries. The insertion tubes for both products are similarly simple with a straw-like design; however, the NT380-Mini tube is 3.7 mm in diameter and the TCu380A is 4.5 mm in diameter. All clinicians placing the products for this study were highly experienced with the TCu380A; though insertion procedures are similar for the products, clinicians were trained on the NT380-Mini placement and practiced (using uterine models) prior to the study.

We recruited healthy participants seeking contraception who were aged 16–40 years, non-pregnant, sexually active with a male partner, had regular menstrual cycles (21–35 days when not using hormones), and were willing to use only the study IUD for contraception. We limited parous enrollment to a maximum of 20%, to better evaluate the product in a nulliparous population. We excluded participants with the following characteristics: currently pregnant, intention to become pregnant within 37 months, known infertility of patient or partner, history of allergy or sensitivity to copper, use of injectable contraception in the last 9 months without two spontaneous menses, within 30 days of abortion/delivery, and medical contraindications.^19^ The full inclusion/exclusion criteria are listed in the online appendix.

At screening, we collected medical history, demographic data (age, parity, level of education, race/ethnicity, marital status), previous contraceptive use, and other data. In most situations, eligible participants enrolled (had IUD placement) at screening. Clinicians attempted IUD placement if the uterus sounded to at least 55mm; if placement was not successful after two attempts, the participant was discontinued.

Participants returned for clinic visits at 6 weeks and 3, 6 and 12 months; at 9 months, we collected key information from participants via phone. At clinic visits we conducted string checks to confirm IUD placement. Site staff entered data into the proprietary electronic data capture system, which ran background logic and consistency checks. Additional logic and consistency checks were performed with statistical software and queries were written to ensure accuracy of data. Verification of entered data was done to ensure data entry matched the source documents. The sponsor's study clinicians conducted blinded review of adverse events from all sites and provided standardized coding for regulatory purposes. Site clinicians performed ultrasonography for missing strings or if clinically indicated. Clinicians recorded a primary reason for IUD discontinuation and any additional contributing reasons for early removal. At clinic/phone visits, we asked participants: “How satisfied are you with the study device?” and “Would you recommend this IUD to others?” Participants also recorded responses to these questions on a diary at 6-days post-IUD placement.

### Randomisation and masking

We stratified randomisation by site and parity (0 or 1+), in a 4:1 ratio (NT380-Mini:TCu380A), using central computer-generated assignments. Clinicians and staff concealed product identity from the participant, by discarding packaging and shielding the IUD from view. Participants remained blinded until product removal. The statistical team at Health Decisions created the randomisation schedule and used block sizes of five to randomly assign a single TCu380A to one position within each block. The team coordinated the upload of the schedule into the proprietary electronic data capture system. Once the randomisation schedule was uploaded into the system, it was then held by the quality management team in a secure limited access location. For scheduled DSMB reviews, the lead biostatistician requested access to the randomisation schedule to create the output by treatment group.

The DSMB reviewed interim safety and efficacy results on three occasions. The information was provided by the assigned biostatistician at Health Decisions. Operational bias was minimized by providing only blinded (aggregate) tables in the open session with the trial's sponsors. In the closed sessions of the DSMB meetings, the biostatistician reviewed tables by treatment group. At each meeting, the DSMB recommended that the trial continue, since no safety or efficacy measures crossed the thresholds for concern.

### Outcomes

The main endpoint for this comparative analysis was IUD discontinuation at or before 12 months (365 days). We examined primary reasons for discontinuation and grouped bleeding and pain adverse event (AE) removals into one category. We classified expulsions as spontaneous complete or partial (when any part of the IUD was in the cervix or just at the internal os by exam or sonography). We defined accidental IUD self-removal as a distinct patient-reported outcome (separate from expulsion) when it occurred while removing menstrual hygiene products. The remaining categories included non-medical removals (desire for pregnancy, not sexually active, withdrew consent) and other medical reasons, including removal due to pregnancy. To measure the broader impact of specific bleeding and pain side effects on early removal, we examined these non-primary contributing reasons: increased menstrual bleeding, intermenstrual bleeding/spotting, dysmenorrhea, and intermenstrual pelvic pain.

For product satisfaction, participants reported one of four responses: highly satisfied, satisfied, dissatisfied, or highly dissatisfied. Participants gave yes/no answers as to whether they would recommend the IUD to others.

### Statistical analysis

Using a fixed study size as calculated for the primary endpoint (contraceptive efficacy), the protocol's stated power calculation for comparative analyses focused on IUD continuation rates in the first year of product use. We estimated that a study of 1000 participants, randomised in a 4:1 ratio favouring the NT380-Mini, would provide 87% power via the logrank test to detect a clinically important 10% higher 12-month continuation rate with the NT380-Mini, assuming the TCu380A rate was 70% using a two-sided alpha of 0·05, and 5% loss to follow-up. We determined the trial's overall study size in consultation with the FDA, to adequately assess safety and efficacy of the NT380-Mini for a new drug application. We chose a 4:1 ratio favouring the NT380-Mini because of budgetary constraints and the need to accumulate enough analysis cycles for adequate precision to estimate contraceptive failure of the NT380-Mini (the target upper 95% confidence interval was 1 pregnancy per 100 women-years of use). Secondary endpoints for comparisons to the TCu380A (the material presented here) were informational, not regulatory.

For this analysis, we included only nulliparous participants: nulligravid women and previously gravid women who only had miscarriages/terminations. We used the Kaplan–Meier product limit method to estimate 12-month cumulative probabilities (and associated 95% confidence intervals) for IUD continuation and primary discontinuation reasons, and used logrank statistics to compare products. We did additional discontinuation analyses to estimate probabilities for type of expulsion and for specific bleeding/pain reasons. The latter tallies included any primary bleeding/pain adverse event reasons as well as any non-primary contributing bleeding/pain reasons for removal; this approach enabled participants’ multiple bleeding and pain problems to be considered. We censored participants who were lost to follow-up or withdrew consent, by analysing their last documented date of product use.

For satisfaction and product recommendations at each timepoint, we used chi-square statistics to test for product differences. We did a supporting analysis using last-observation-carried-forward to better understand the potential role of attrition on these outcomes; this entailed applying the last opinions on the product (at or before IUD discontinuation) to subsequent timepoints to maintain a steady sample size for analysis. We did not make any adjustments for interim analyses or allowances for multiplicity. For all analyses, we used SAS® V9·4 (SAS Institute, Inc., Cary, NC, USA) and the CONSORT statement was used for reporting this analysis.

### Role of the funding source

Only author-employees at *Eunice Kennedy Shriver* National Institute of Child Health and Human Development (NICHD) had input on the study design, analysis, interpretation, and manuscript. The other funders provided only financial assistance and/or study product for the trial; they had no role in study design, analysis, interpretation, or manuscript writing. All authors had full access to the data from their centre and had final responsibility for the decision to submit for publication.

## Results

**Figure 1:**
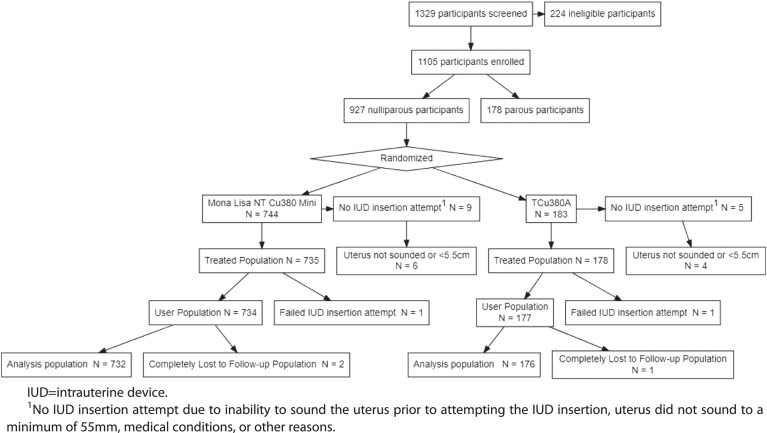
**Analysis population flow diagram for nulliparous participants**.

We screened 1329 potential participants and enrolled 1105, of whom 927 (83·9%) were nulliparous and included in this analysis. The 927 nulliparous participants included 744 randomised to the NT380-Mini and 183 to the TCu380A; successful IUD placement occurred in 734 (98·7%) NT380-Mini and 177 (96·7%) TCu380A participants (Figure 1). Baseline characteristics were similar in the two groups (Table 1). Notably, about 9% of participants used a copper IUD previously and 13% used a hormonal IUD previously.

**Table 1:** Characteristics of participants at baseline, intent-to-treat population.

	Treatment	
	NT380-Mini(*N* = 744)	TCu380A(*N* = 183)
Characteristics	*n* (%)	*n* (%)
**Age**: mean (SD)	25·4 (4.8)	25·6 (4.5)
**Ethnicity**		
Hispanic or Latino origin	108 (14.5)	21 (11·5)
Not Hispanic or Latino origin	631 (84.8)	162 (88·5)
Not reported	5 (0·7)	0 (0·0)
**Race**		
American Indian or Alaska native	6 (0·8)	1 (0·5)
Asian	76 (10·3)	15 (8·2)
Native Hawaiian or other Pacific Islander	4 (0·5)	0 (0·0)
Black or African American	52 (7·1)	14 (7·7)
White	531 (72·1)	134 (73·2)
Multiple	50 (6·8)	16 (8·7)
Other	17 (2·3)	3 (1·6)
**Highest level of education**		
Less than high school	4 (0·5)	0 (0·0)
High school grad or equivalent	61 (8·2)	10 (5·5)
Some college, no degree	233 (31·3)	48 (26·2)
2-yr College/Associate's	42 (5·6)	17 (9·3)
4-yr College/Bachelor's	292 (39·2)	83 (45·4)
Master or equivalent	94 (12·6)	23 (12·6)
Doctorate or equivalent	18 (2·4)	2 (1·1)
**Gravidity**		
Never pregnant	620 (83·6)	153 (83·6)
Previous pregnancy without live birth	122 (16·4)	30 (16·4)
**Marital status**		
Never Married	650 (87·4)	156 (85·2)
Married	60 (8·1)	23 (12·6)
Separated	7 (0·9)	1 (0·5)
Divorced	27 (3·6)	3 (1·6)
**BMI** (kg/m^2^): mean (SD)	25·9 (6·3)	25·9 (6·3)
**Menstrual pattern**		
Average Cycle Length (# of Days)		
Mean (SD)	28·5 (2·1)	28·9 (4.9)
**Usual flow duration(days)**		
Mean (SD)	4.9 (1·1)	4.9 (1·06)
**Heaviest volume of menstrual flow**		
Light	75 (10·1)	19 (10·4)
Moderate	427 (57·5)	107 (58·8)
Heavy	241 (32·4)	56 (30·8)
**Any history of irregular periods?**		
Yes	149 (20·1)	38 (20·9)
No	594 (79·9)	144 (79·1)
**Usual bleeding pattern**		
No Bleeding	2 (0·3)	1 (0·5)
Spotting Only	9 (1·2)	1 (0·5)
Irregular Pattern	9 (1·2)	1 (0·5)
Regular Pattern	723 (97·3)	180 (98·4)
**Usual bleeding or spotting between periods?**		
Yes	44 (5·9)	11 (6·0)
No	699 (94·1)	172 (94·0)
**Participant's overall opinion of her bleeding pattern**		
Acceptable	716 (96·4)	181 (98·9)
Not Acceptable	27 (3·6)	2 (1·1)
**Previous use of copper IUD**		
Yes	63 (8·5)	21 (11·5)
No	680 (91·5)	162 (88·5)
**Previous use of hormonal IUD**		
Yes	97 (13·1)	28 (15·3)
No	646 (86·9)	155 (84·7)

Missing data (*n*): BMI [NT380-Mini(12) and TCu380A(6)], Race [NT380-Mini(8)], Gravidity [NT380-Mini(2)], Menstrual data [NT380-Mini(6) and TCu380A(1)].

**Figure 2:**
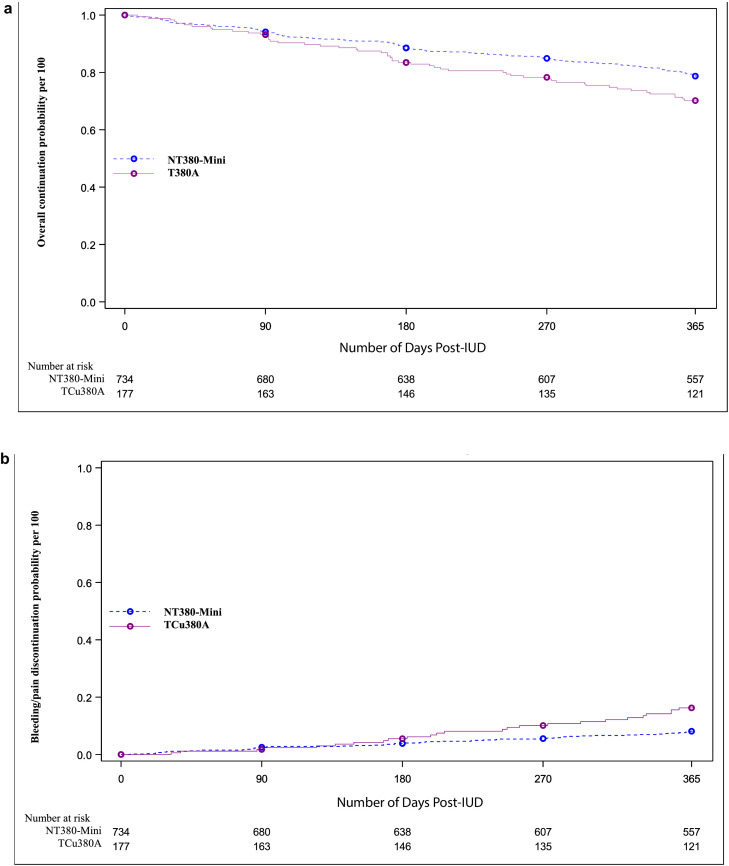
**a: 12-Month probabilities of overall product continuation**. Kaplan–Meier plot of 12-month probabilities of overall continuation per 100. NT380-Mini (78.7%) versus TCu380A (70.2%), logrank test *p*-value=0.014. **b: 12-Month probabilities of discontinuation due to bleeding and/or pain**. Kaplan–Meier plot of 12-month probabilities per 100. NT380-Mini (8.1%) versus TCu380A (16.2%), logrank test *p*-value=0.003.

Twelve-month continuation rates were 78·7% (95% CI:72·9–84·5) and 70·2% (95% CI:59·7–80·7) for the NT380-Mini and the TCu380A, respectively (*p* = 0·014) (Figure 2a). Overall, the most frequent primary reasons for discontinuation were adverse events due to pain/vaginal bleeding: cumulative probabilities of discontinuation were 8·1% (95% CI:5·9–10·3) and 16·2% (95% CI:10·2–22·3) of NT380-Mini and TCu380A participants, respectively (*p* = 0·003, Figure 2b and Table 2). Twelve-month IUD expulsion rates were 4·8% (95% CI:3·0–6·5) in NT380-Mini users and 8·9% (95% CI:4·1–13·8) in TCu380A users, *p* = 0·023; partial expulsions contributed most to this difference (Table 2). Probabilities of accidental self-removals of the IUD were 2·9% and 0·6% in NT380-Mini and TCu380A users, respectively, *p* = 0·11.

**Figure 3:**
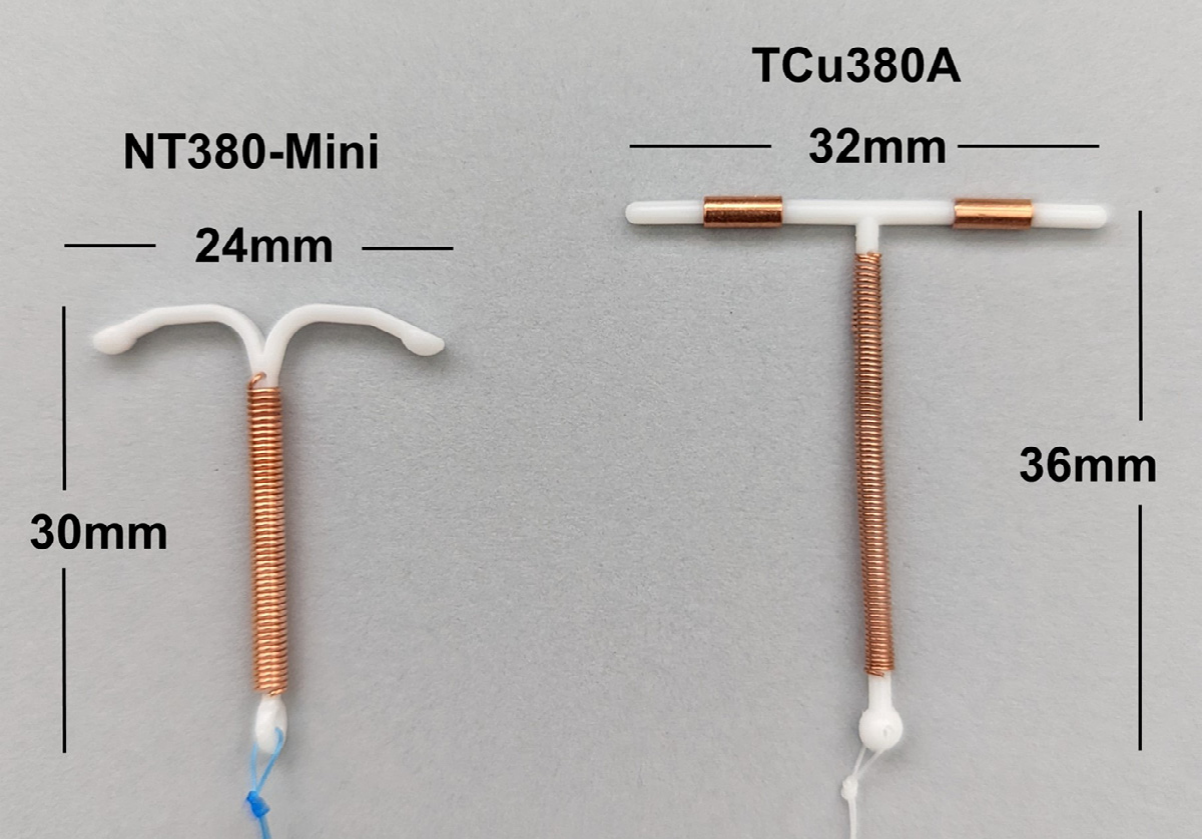
**Dimensions of TCu380A and NT380-Mini**.

**Table 2:** 12-Month IUD discontinuation probabilities by reason and product, user population.

Outcome	NT380-Mini (*N* = 734)		TCu380A (*N* = 177)		*p*-value
	Probability (%)	*n* (95% CI)	Probability (%)	*n* (95% CI)	
**Continued use**	78·7	581 (72·9–84·5)	70·2	125 (59·7–80·7)	0·014
**IUD discontinuation**	21·3	153 (18·3–24·3)	29·8	52 (23·0–36·7)	0·014
**Lost to follow-up**^a^	3·7	25 (2·2–5·3)	3·4	5 (0·2–6·5)	0·764
**Primary reasons for discontinuation**					
**Any adverse event removal due to bleeding and/or pain**^b^	8·1	54 (5·9–10·3)	16·2	25 (10·2–22·3)	0·003
**Any expulsions**	4·8	32 (3·0–6·5)	8·9	15 (4·1–13·8)	0·023
Spontaneous complete expulsion	1·9	13 (0·8–3·1)	1·7	3 (0·0–4·1)	0·978
Partial expulsion (IUD in cervix or at os)	2·9	19 (1·5–4·3)	7·3	12 (2·8–11·8)	0·005
**Accidental self-removal of IUD**	2·9	19 (1·5–4·2)	0·6	1 (0·0–1·9)	0·112
**Other medical reasons**^c^	4·1	26 (2·5–5·7)	3·6	5 (0·3–6·9)	0·739
**Other reasons**^d^	3·5	22 (2·0–5·0)	4·0	6 (0·6–7·4)	0·693
**All bleeding and pain reasons (includes primary adverse events and contributing reasons for removal)**					
Intermenstrual pain^f^	2·5	16 (1·2–3·8)	10·1	15 (5·0–15·2)	<0·001
Menstrual pain^f^	4·4	29 (2·7–6·0)	8·3	12 (3·6–13·1)	0·084
Increased menstrual bleeding^e^^,^^f^	4·0	26 (2·4–5·6)	6·8	10 (2·5–11·2)	0·160
Intermenstrual bleeding/spotting^f^	1·9	12 (0·8–3·0)	3·5	5 (0·3–6·7)	0·251

a Separate survival analysis with lost-to-follow-up categorized as an endpoint for comparative purposes.

b A participant with more than one bleeding/pain adverse event is tallied once for these 79 person-events.

c Includes malposition of IUD, vaginal disorders, pregnancy, anxiety, acne, miscellaneous.

d Includes desire for pregnancy, not sexually active, withdrew consent.

e Increased menstrual bleeding includes increased menstrual blood loss, prolonged menses, increased frequency of menses.

f A participant may have more than one reason. *p*-values from Kaplan–Meier logrank tests.

Considering all bleeding and pain removal reasons (primary adverse events and contributing bleeding and pain reasons for removal), the probability of removal for intermenstrual pain was statistically significantly lower for the NT380-Mini (2·5%; 95% CI:1·2–3·8) compared to the TCu380A (10·1%; 95% CI:5·0–15·2), *p* < 0·001) (Table 2). Removal rates for menstrual pain were 4·4% versus 8·3% (*p* = 0·08), removal rates for increased menstrual bleeding were 4·0% versus 6·8% (*p* = 0·16), and removal rates for intermenstrual bleeding/spotting were 1·9% versus 3·5% (*p* = 0·25), in NT380-Mini and TCu380A users, respectively. Incidence of all types of adverse events and detail on serious adverse events can be found in Supplementary Table 1.

Product satisfaction exceeded 92% and product recommendations were 90% or higher at all timepoints for both products (Table 3). Satisfaction levels with the products were not statistically different for any timepoint. Product recommendations were higher for the NT380-Mini at 3- and 12-months, (*p* = 0·002 and *p* < 0·001, respectively). In our supporting analysis using last-observation-carried-forward to mitigate the potential role of attrition (data not shown), the results reported in Table 3 did not change.

**Table 3:** Satisfaction with product by time post-insertion and product, user population.

	6-Days		6-Weeks		3-Months^a^		6-Months		9-Months		12-Months^b^	
	NT380-Mini	TCu380A	NT380-Mini	TCu380A	NT380-Mini	TCu380A	NT380-Mini	TCu380A	NT380-Mini	TCu380A	NT380-Mini	TCu380A
Satisfaction items	(*N* = 703)	(*N* = 169)	(*N* = 698)	(*N* = 166)	(*N* = 669)	(*N* = 157)	(*N* = 618)	(*N* = 142)	(*N* = 586)	(*N* = 129)	(*N* = 472)	(*N* = 100)
**How satisfied are you with the study product?** no. (%)												
Highly satisfied	379 (53·9)	77 (45·6)	320 (45·8)	74 (44·6)	378 (56·5)	82 (52·2)	356 (57·6)	74 (52·1)	343 (58·5)	66 (51·2)	313 (66·3)	59 (59·0)
Satisfied	293 (41·7)	83 (49·1)	359 (51·4)	84 (50·6)	278 (41·6)	67 (42·7)	247 (40·0)	57 (40·1)	220 (37·5)	56 (43·4)	151 (32·0)	35 (35·0)
Dissatisfied	25 (3·6)	8 (4·7)	18 (2·6)	7 (4·2)	12 (1·8)	8 (5·1)	14 (2·3)	9 (6·3)	19 (3·2)	6 (4·7)	8 (1·7)	6 (6·0)
Highly dissatisfied	6 (0·9)	1 (0·6)	1 (0·1)	1 (0·6)	1 (0·1)	0 (0·0)	1 (0·2)	2 (1·4)	4 (0·7)	1 (0·8)	0 (0·0)	0 (0·0)
**Would you recommend this IUD to others?**^c^												
no. (%)												
Yes	665 (94·6)	157 (92·9)	661 (94·7)	153 (92·2)	647 (96·7)	143 (91·1)	598 (96·8)	134 (94·4)	564 (96·4)	124 (96·1)	460 (97·5)	90 (90·0)
No	38 (5·4)	12 (7·1)	37 (5·3)	13 (7·8)	22 (3·3)	14 (8·9)	20 (3·2)	8 (5·6)	21 (3·6)	5 (3·9)	12 (2·5)	10 (10·0)

a For recommendation variable, *p*-value=0·002.

b For recommendation variable, *p*-value <0·001.

c At 9-months, based on *n* = 585 due to missing data (NT380-Mini group).

## Discussion

This randomised trial involving nulliparous participants demonstrated important first-year benefits of the smaller copper IUD compared to the larger standard product: higher 12-month continuation rates, fewer expulsions, fewer removals due to bleeding and/or pain, and higher product recommendations at some time periods. Our results address a significant, but largely forgotten matter, that was never adequately examined in the tranche of comparative copper IUD trials that started in the 1970s and ended over 20 years ago.

Our key 12-month measures for the TCu380A (70·2% overall continuation probability, 16·2% removal due to bleeding and/or pain adverse events, and 8·9% expulsion) are similar to previous randomised trials and help validate our findings. For example, a 1970s study with a 50% nulliparous population reported 12-month rates of 69·7% (continuation), 14·2% (removal rate for bleeding and/or pain), and 7·1% (expulsion rate).^1^ More recently, an observational study in the USA reported an overall 12-month continuation rate of 85% for the TCu380A^20^ and a 6% first-year expulsion rate among nulliparous users.^21^

We found a statistically significant higher probability of partial IUD expulsion in the TCu380A group compared to the NT380-Mini. Given lack of blinding of providers, their discretion at doing ultrasounds, and the possibility that the TCu380A group might have had more ultrasounds (leading to surveillance bias in detecting partial expulsions), we examined the frequency of ultrasounds. We found both treatment groups had the same percentage of ultrasounds in the first year (3·55% of NT380-Mini participants and 3·70% of TCu380A participants, *p*-value 0·664, data not shown). Thus, it is unlikely that this affected our results. The higher expulsion rate in the TCu380A group supports the notion that the TCu380A may be incompatible with the uterine cavities of some nulliparous users. Increased frequency or intensity of uterine contractions with a larger product *in situ,* may push a product toward the lower uterine cavity. Though IUDs have been shown to move toward and away from the fundus over time,^22^ a product that becomes temporarily or permanently malpositioned might also produce unacceptable bleeding and pain side effects that lead to early removal. As described in the 1970 US patent for the TCu380A frame, “the device is of such a size that the crossbar of the “T” may exert some lateral pressure on the endometrial wall of the uterus. X-ray studies indicate that the ends of the crossbar will become slightly embedded in the endometrium suggesting a gentle anchoring phenomenon”.^23^

While IUD size is one factor, our findings may be due to other differences between the products. For example, the shorter arms of the NT380-Mini splay outward from the stem and flex upward (the Nova-T design used on hormonal IUDs), whereas the TCu380A arms are rigidly perpendicular to the stem (Figure 3). Also, the TCu380A has copper on the arms/stem, while the NT380-Mini relies on copper affixed only to the stem. Sivin and Stern^1^ showed that the TCu380A had higher contraceptive efficacy, yet higher rates of removal due to bleeding and pain compared to the same frame with 200mm^2^ copper on the stem only. In the review of clinical records at the UK's NHS, investigators found that only “smallness” explained why one copper IUD performed better than the standard (larger) product in a nulliparous population.^13^ In that study, the two copper IUDs were equivalent in all aspects except size, yet patients’ complaints of bleeding/pain and incidence of early removals were twice as high with the standard (larger) copper IUD. An observational cohort study of parous/nulliparous copper IUD users in six European countries, found that smaller IUD arm-spans and other design differences (lower copper content, horseshoe-shaped frames, flex-up arms, and arms without copper) were associated with fewer side effects and fewer early IUD removals; however, in the nulliparous-only subpopulation, these patterns were largely absent.^24^ Of note, smaller frames on hormonal IUDs do not show benefits in the post-placement period compared to larger framed products.^25^ Our findings are not necessarily generalizable to any other device that is simply smaller than the TCu380A.

Our satisfaction results reflect the thoughts of current users at the time of interview. These measures are imperfect; attrition from dissatisfaction results in retention of higher percentages of satisfied users, which generally biases comparisons toward neutrality. Our supporting analysis to combat the potential impact of attrition also did not reveal major product differences. The reasons for apparent inconsistencies between opinion questions and early removals due to bleeding and/or pain are unclear. Perhaps opinion questions are too broad, and/or pre-placement counselling about potential bleeding/pain led participants to expect far worse, and/or social desirability bias concealed participants’ true feelings. Perhaps we do not have enough information about prior experiences, expectations or other factors that are too complex at the individual patient level to understand these nuances. Regardless, in light of unknown validity of what these data measure, incidence of early removal and the underlying cause is perhaps a more reliable indicator of product acceptability.

Our trial has strengths and weaknesses. One important strength is the location of the trial. We chose the USA, where only one copper IUD is currently approved and marketed; this avoided recruitment challenges and perhaps ethical issues we may have encountered in other countries (concerns over randomising nulliparous participants to larger products when smaller products are available). An important weakness is that we cannot claim that small size explains all the benefits, since the NT380-Mini differs in other ways from the TCu380A. Due to budgetary constraints and study size requirements for regulatory review of a new IUD, our 4:1 randomisation ratio may have prevented optimal comparative capabilities. Both products in our trial have full approval and are marketed in many EU countries. During the trial, the DSMB, the IRBs, and the USFDA reviewed incidence of events and continuously affirmed participant safety. Full safety information on the complete population (parous and nulliparous) and complete time period will be submitted to the USFDA for regulatory review.

Approximately 8·4% of women worldwide use IUDs (about 160 million people); of all reversible contraceptives, the IUD is second only to condoms in terms of highest prevalence of use.^26^ While this estimate includes hormonal products, only upper-income countries have substantial use of hormonal IUDs; still, even in the UK for example, new IUD users select copper products 36% of the time.^27^ Nulliparous women (which includes the nulligravid subset) are increasingly choosing IUDs; in the USA, the annual percent change in prevalence of IUD use among nulligravid women was 11% from 2008 to 2017^28^ largely due to proven safety of intrauterine contraception and the availability of hormonal devices. More recently, growing interest in female-controlled, non-hormonal contraception led to the development and approval of a new non-hormonal vaginal gel product in 2020; however, that product unfortunately fails to prevent pregnancy at an estimated first-year rate of 27·5 per 100 person-years.^29^ Better non-hormonal contraceptives, with proven benefits for nulliparous women, serve broad societal needs of equity, regardless of parity.

In conclusion, this trial found important benefits of the NT380-Mini copper IUD compared to the TCu380A in the first year. For nulliparous users who try the copper IUD for the first time, the impact of these benefits may be of lasting importance; those with positive experiences will likely consider using a copper IUD in subsequent life stages. The copper IUD technology remains the only long-acting, non-hormonal form of reversible contraception; any improvements in performance stand to benefit even broader populations of potential users. Complete 3-year safety and efficacy findings will be published when available, to more fully understand product performance.

## Contributors

DH conceived the trial, secured initial funding, and wrote the first draft of the manuscript, DLB secured funding and expanded the scope of the trial, CD oversaw the randomisation plan, PLC, CD, and DH verified the underlying data, all authors contributed to protocol development and critical review and feedback on the manuscript. All authors had full access to the data from their centre and had final responsibility for the decision to submit for publication. All authors read and approved the manuscript.

## Data sharing statement

Metadata will be available for public access after final regulatory review by the USFDA or two years after publication of this manuscript, whichever comes first. Interested parties may submit a request via email to the corresponding author (or subsequently named alternative) with a brief sketch of what will be done with the data. De-identified individual participant data and a data dictionary defining each raw data variable used in this analysis will be made available. No additional study documents will be provided.

## Declaration of interests

DH, PLC, and KN's institution (FHI 360) received funding from Bill & Melinda Gates Foundation and Mona Lisa, N.V. to conduct this study. The following investigators’ institutions received contract funding from FHI 360 (with funds originating from Bill & Melinda Gates Foundation) to help conduct this study: CAS, ID, BAC, ABG, CD, ALN. The following investigators’ institutions received contract funding from *Eunice Kennedy Shriver* National Institute of Child Health and Human Development (NICHD) to help conduct this study: DKT, JTJ, MDC, KOW, SBT, JLK, CD, MAT, DFA, PMC, AEB. These two employees of the NIH were supported by *Eunice Kennedy Shriver* National Institute of Child Health and Human Development (NICHD) to help conduct this study: DLB and JEB. No author received personal financial payments for this work. No author has financial relationships with Mona Lisa, N.V. DH reports board membership on Society of Family Planning. CAS reports contracts or grants to institution from Bayer Pharma, Sebela Pharma, and Danco Pharma; royalties to self and institution from Atheneum Pharma; payment for expert testimony from Center for Reproductive Rights, Planned Parenthood Federation and ACLU; a U.S. Provisional Patent Application No. 62/777,369; honoraria from American Board of Obstetrics & Gynecology. DKT reports contracts or grants to institution from The Laura and John Arnold Foundation, Society of Family Planning Research Fund, the William and Flora Hewlett Foundation, the Willard L. Eccles Foundation, the Intermountain Community Care Foundation, Bayer Women's Health, Organon, Cooper Surgical, Medicines360, Sebela Pharmaceuticals and anonymous foundation. MCD reports contracts to institution from Chemo Research SL, Evofem, HRA Pharma, Medicines360, Merck, Sebela; consulting fees from Estetra SRL, FHI360, Libbs, Mayne, Medicines360; speaking honorarium from Gedeon Richter, Mayne; payment/reimbursement of expenses for travel to meetings to present research from Medicines360; payment/reimbursement of expenses for travel to meetings to present research or attend Advisory Board from Estetra SRL; payment/reimbursement of expenses for travel to attend Advisory Board from TherapeuticsMD; Advisory Board meetings with honorarium from Evofem, Fuji Pharma, Mayne, Merck, Searchlight and TherapeuticsMD; honorarium for duties as Deputy Editor of the journal Contraception. KOW reports grants or contracts to institution from Bayer Pharmaceuticals and honoraria for a remote consultancy from Bayer Pharmaceuticals. SBT reports contracts to institution from Sebela, Medicines 360, Bayer Healthcare, Chemo Research, S.L., Merck & Co.; DSMB for Merck & Co.; Advisory Board for Bayer Healthcare; Society of Family Planning board member; American Board of Obstetrics & Gynecology (Complex Family Planning Division Member). BAC reports contracts or grants to institution from Medicines360, Sebela, and Mylan; member of American College of Obstetrics and Gynecology working group. ABG reports grants or contracts to institution from Merck; royalties from Uptodate; consulting fees from Society of Family Planning; board member of Society of Family Planning. ALN reports contracts or grants to institution from Merck, Mylan, Myovant, Sagami Rubber Industries, Sebela; consulting fees from Agile Therapeutics, Bayer HealthCare, Mayne Pharma, Pfizer, TherapeuticsMD; payment or honoraria for lectures, presentations, speakers bureaus, manuscript writing or educational events from Agile Pharma, Bayer HealthCare, Myovant Sciences, Merck, TherapeuticsMD; support for attending meetings and/or travel from Agile Pharma, Bayer HealthCare, Myovant Sciences, Merck, Pfizer, TherapeuticsMD. MAT reports Board member of American Society for Reproductive Medicine. DFA reports grants or contracts to institution from Bayer Healthcare, Dare Biosciences, Estetra, Myovant, and ObsEva; consulting fees from Bayer Healthcare, Exeltis, Mithra, Lupin, ObsEva, Mithra; honoraria for lecture from Exeltis; DSMB member; Board of directors for Diczfalusy Foundation; stock ownership in InnovaGyn, Inc.; stock options with Agile Therapeutics. PMC reports consulting fees from Bayer to provide remote IUD consultative support to clinicians; payments to institution from Bayer for clinical research. AEB reports grants or contracts to institution from Merck & Co, Inc (USA) and Scope/Chemo (Spain); honorarium and travel to meetings from TherapeuticsMD; support for related work and travel as associate editor for ACOG Clinical Updates; support for travel/honorarium from American Board of Obstetrics & Gynecology. BK reports grants or contracts to institution from Contramed Pharmaceuticals (Sebela), Evofem Biosciences, Gynuity Health Projects; royalties from Uptodate; consulting fees from Merck. The remaining authors do not have any related relationships to disclose: JTJ, KN, ID, PLC, JLK, CD, JEB, DLB.

## Appendix Supplementary materials

Image, application 1

Image, application 2
