# Supplementary material for: Continuation rates of two different-sized copper intrauterine devices among nulliparous women: Interim 12-month results of a single-blind, randomised, multicentre trial
Source: eClinicalMedicine. 2022 Jul 16;51:101554. doi: 10.1016/j.eclinm.2022.101554 (PMC9294241; doi:10.1016/j.eclinm.2022.101554)
Supplement: Supplementary file 1 [file mmc1.pdf]

NICHD/FHI 360

Clinical Research Protocol

A multi-center, single-blind, randomized clinical trial to compare two copper IUDs: Mona Lisa NT Cu380 Mini and ParaGard

Protocol Number:	CCN016
Version Date:	Final version 03.00 19Dec2018
Investigational Product:	Mona Lisa® NT Cu380 Mini
Development Phase:	Phase III
IND Number:	132657
Sponsor/IND Holder<br><br><br><br>Co-Sponsor	FHI 360<br>359 Blackwell Street, Suite 200<br>Durham, NC 27701<br><br>National Institutes of Health<br>*Eunice Kennedy Shriver* National Institute of Child Health & Human Development (NICHD)<br>Contraceptive Clinical Trial Network (CCTN) (Female)<br>6100 Executive Boulevard, Room 8B13<br>Bethesda, MD 20892 USA
Funding Organizations:	The Bill and Melinda Gates Foundation (FHI 360 portion) and the *Eunice Kennedy Shriver* National Institute of Child Health & Human Development (NICHD) of the National Institutes of Health

Principal Investigators:	<u>FHI 360 Sites</u><br>Courtney Schreiber, MD, MPH: Perelman School of Medicine at the University of Pennsylvania<br>Beatrice A. Chen, MD, MPH: University of Pittsburgh<br>Anita Nelson, MD: Essential Access Health<br>Alisa Goldberg, MD: Planned Parenthood League of Massachusetts,<br>Ila Dayananda, MD: Planned Parenthood New York<br><br><u>NICHD Sites</u><br>David Archer, MD: Eastern Virginia Medical School<br>Anne Burke, MD: Johns Hopkins University<br>Paula Castaño, MD, MPH: Columbia University<br>Mitchell Creinin, MD: University of California, Davis<br>Philip Darney, MD, MSc: University of California, San Francisco<br>Jeffrey Jensen, MD, MPH: Oregon Health & Science University<br>Subsite: Bliss Kaneshiro, MD, MPH: University of Hawaii<br>Stephanie Teal, MD, MPH: University of Colorado Denver<br>Michael A. Thomas, MD: University of Cincinnati<br>David Turok, MD: University of Utah<br>Katharine White, MD, MPH: Boston Medical Center Corporation
Medical Monitors:	**FHI 360**<br>Name: Kavita Nanda, MD, MHS<br>Telephone: 919-544-7040, ext. 11507<br>Fax: 919-544-7261<br>E-mail: knanda@fhi360.org<br><br>**NICHD**<br>Name: Jill Long, MD, MPH, MHS<br>Telephone: 301-496-1662<br>E-mail: jill.long@nih.gov

Program Directors:	**FHI360**<br>Name: David Hubacher, PhD<br>Telephone: 919 544 7040, ext 11223<br>Fax: 919 544 7261<br>E-mail: dhubacher@tlli360.org<br><br>**NICHD**<br>Name: Diana Blithe, PhD<br>Telephone: 301 435 6990<br>E-mail: blithcd@nih.gov
Coordinating Center:	Name: Clint Dart, MS<br>Health Decisions<br>2510 Meridian Parkway, Suite 100<br>Durham, NC 27713, USA<br>Telephone: 919-967-1111<br>E-mail: cdart@healthdec.com

Approval:

Diana L. Blithe -S Digitally signed by Diana L. Blithe -S
Date: 2018.12.20 15:13:32 -05'00'

Diana Blithe, PhD — *Date*
Program Director, NICHD

12/19/2018

David Hubacher, PhD — *Date*
Senior Epidemiologist, FHI 360

PROTOCOL AGREEMENT

I, the undersigned, will conduct the clinical study as described in the Protocol and will adhere to the *Code of Federal Regulations*, Title 21 and Title 45, Part 46, Good Clinical Practices (GCP), *International Council for Harmonisation (ICH)*, and the Declaration of Helsinki. I have read and understood the contents of the Protocol and Investigator Brochure.

The signature of the investigator below indicates acceptance of the protocol and a complete understanding of the investigator obligations as outlined in Investigator Obligations.

Protocol Number: CCN016

Protocol Title: **A multi-center, single-blind, randomized clinical trial to compare two copper IUDs: Mona Lisa NT Cu380 Mini and ParaGard**

Protocol Date: 19Dec2018

____ __________________

Principal Investigator Signature *Date*

____

Print Name and Title

Site # __________

Site Name ____

Address ____

____

____

____

Phone Number ____

1 BACKGROUND AND RATIONALE 14

2 STUDY PRODUCTS 15

2.1 BRIEF PRODUCT DESCRIPTIONS – STRUCTURE AND REGULATORY STATUS 15

2.1.1 ParaGard (control) 15

2.1.2 Mona Lisa NT Cu380 Mini (test product) 15

2.2 OVERVIEW OF NON-CLINICAL STUDIES 15

2.2.1 Mona Lisa NT Cu380 Mini 16

2.3 OVERVIEW OF CLINICAL STUDIES 16

3 STUDY OBJECTIVES 17

3.1 PRIMARY OBJECTIVE 17

3.2 SECONDARY OBJECTIVES 17

4 STUDY DESIGN 17

4.1 STUDY OVERVIEW 17

4.2 STUDY DURATION 18

5 PRINCIPAL INVESTIGATORS AND RESEARCH SITES 18

5.1 FHI 360 SITES 18

5.2 NICHD SITES 18

6 CRITERIA FOR EVALUATION 19

6.1 PRIMARY ENDPOINT 19

6.2 SECONDARY ENDPOINTS 19

6.3 SAFETY EVALUATIONS 19

7 SUBJECT SELECTION 19

7.1 STUDY POPULATION 19

7.2 INCLUSION CRITERIA 20

7.3 EXCLUSION CRITERIA 20

7.4 ALLOWABLE CRITERIA 21

7.5 NUMBER OF SUBJECTS 21

8 CONCURRENT MEDICATIONS AND TREATMENTS 22

8.1 ALLOWED MEDICATIONS AND TREATMENTS 22

8.2 PROHIBITED MEDICATIONS AND TREATMENTS 22

9 STUDY TREATMENTS 22

9.1 METHOD OF ASSIGNING SUBJECTS TO TREATMENT GROUPS 22

9.2 BLINDING (PARTICIPANT ONLY) 23

9.3 FORMULATION OF TEST AND CONTROL PRODUCTS 23

9.3.1 Formulation of Test and Control Products 23

9.3.2 Packaging and Labeling 24

9.4 PROVISION OF STUDY PRODUCT TO PARTICIPANTS 24

9.4.1 Dosage Regimen 24

9.4.2 Dispensing ...24
9.4.3 Administration Instructions ...25
9.5 SUPPLY OF STUDY PRODUCT AT THE SITE ...25
9.5.1 Storage ...25
9.6 STUDY PRODUCT ACCOUNTABILITY ...25
9.7 MEASURES OF TREATMENT COMPLIANCE ...26
9.8 STUDY PRODUCT DISPOSAL/RETURN ...26
10 STUDY PROCEDURES AND GUIDELINES ... 26
10.1 INVESTIGATOR'S MEETING AND PROTOCOL TRAINING ...26
10.2 CLINICAL ASSESSMENTS AND PROCEDURES ...26
10.2.1 Medical, Demographic, and Reproductive Health History ...27
10.2.2 Vital signs and Pelvic Exam ...27
10.2.3 Ultrasound examination ...27
10.2.4 Pap Test ...28
10.2.5 Laboratory Assessments ...28
10.2.6 Specimen collection and Processing ...28
10.3 SUBJECT ASSESSMENTS ...29
10.3.1 Follow Up Form ...29
10.3.2 Daily Diary ...29
10.3.3 Other Contraceptive Use ...29
10.3.4 Post Placement Pain Forms ...29
11 EVALUATIONS BY VISIT ... 30
11.1 SCREENING AND ENROLLMENT/IUD INSERTION ...30
11.1.1 Screening Procedures ...30
11.1.2 Visit 1 (Enrollment Procedures and IUD Insertion) ...31
11.2 CLINIC-BASED FOLLOW-UP VISITS ...34
11.2.1 Visit 2 (6-weeks post-insertion ± 5 days) ...34
11.2.2 Visit 3 (3 months post-insertion ± 14 days) ...34
11.2.3 Visit 4 (6 months post-insertion ± 14 days) ...34
11.2.4 Visit 5 (12 months post-insertion ± 14 days) ...34
11.2.5 Visit 6 (24 months post-insertion ± 14 days) ...34
11.2.6 Unscheduled visits (any time the participant returns for services outside the schedule) ...35
11.3 TELEPHONE CALL 1, 2, AND 3 (9 MONTHS, 18 MONTHS, AND 30 MONTHS POST-INSERTION ± 14 DAYS) ..36
11.4 EXIT (37 MONTHS POST-INSERTION ± 14 DAYS) OR EARLY DISCONTINUATION VISIT ...37
11.5 TELEPHONE CALL 4 FOR PREGNANCY TEST RESULT (17-DAYS POST-REMOVAL + 3 DAYS) ...39
11.6 EARLY DISCONTINUATION VISIT ...39
12 SAFETY ... 40
12.1 ADVERSE EVENTS ...40
12.2 SERIOUS ADVERSE EXPERIENCES (SAE) ...42
12.2.1 Serious Adverse Experience Reporting ...43
12.3 MEDICAL MONITOR ...44
13 PREGNANCY DETERMINATION AND FOLLOW UP ... 44
14 MONITORING ... 45
14.1 CLINICAL SITE MONITORING ...45

14.1.1 *Quality control and quality assurance* ... 46
14.1.2 *Source documents and access to source data/documents* ... 46
14.2 REGULATORY ... 47
15 DISCONTINUATION AND REPLACEMENT OF SUBJECTS ... 47
15.1 EARLY DISCONTINUATION OF STUDY PRODUCT ... 47
15.1.1 *Lost to Follow-Up* ... 48
15.2 WITHDRAWAL OF SUBJECTS FROM THE STUDY ... 48
15.3 REPLACEMENT OF SUBJECTS ... 48
15.4 STUDY PRODUCT ERRORS ... 48
16 PROTOCOL DEVIATIONS ... 49
17 AMENDMENTS TO THE PROTOCOL ... 49
18 DATA SAFETY AND EFFICACY MONITORING ... 50
18.1 DATA AND SAFETY MONITORING BOARD (DSMB) ... 50
19 STATISTICAL METHODS AND CONSIDERATIONS ... 50
19.1 SAMPLE SIZE ... 50
19.2 STATISTICAL ANALYSES ... 51
19.2.1 *Primary analysis* ... 51
19.2.2 *Analysis of secondary endpoints* ... 52
20 DATA COLLECTION, RETENTION AND MONITORING ... 53
20.1 ELECTRONIC DATA CAPTURE SYSTEM ... 53
20.2 DATA HANDLING AND PROCESSING ... 53
20.3 CONFIDENTIALITY AND REPORTING OF RESULTS ... 53
20.4 ARCHIVAL OF DATA ... 53
20.5 AVAILABILITY AND RETENTION OF INVESTIGATIONAL RECORDS ... 53
20.6 SUBJECT CONFIDENTIALITY ... 54
21 ADMINISTRATIVE, ETHICAL, REGULATORY CONSIDERATIONS ... 54
21.1 INSTITUTIONAL REVIEW BOARDS AND INDEPENDENT ETHICS COMMITTEES ... 54
21.2 PROTOCOL REGISTRATION ... 54
21.3 RISKS AND BENEFITS ... 55
21.3.1 *Risks* ... 55
21.3.2 *Benefits* ... 56
21.4 INFORMED CONSENT ... 56
21.5 CONFLICTS OF INTEREST ... 57
21.6 CONFIDENTIALITY ... 57
21.7 PUBLICATIONS ... 57
21.7.1 *Publication Policy* ... 58
21.7.2 *Authorship eligibility* ... 58
22 REFERENCES ... 59
23 INVESTIGATOR OBLIGATIONS ... 60
24 APPENDIX 1: SCHEDULE OF ASSESSMENTS ... 61

25 APPENDIX 2: IUD CONFIRMATION PROCESSES 64

26 APPENDIX 3: DAILY DIARY.. 65

27 APPENDIX 4: OTHER CONTRACEPTIVE USE FORM 66

28 APPENDIX 5: POST PLACEMENT PAIN FORM – CLINIC 67

29 APPENDIX 6: POST PLACEMENT PAIN FORM – HOME 68

30 APPENDIX 7: PARAGARD PRESCRIBING INFORMATION 69

31 APPENDIX 8: MONA LISA NT CU380 MINI PRESCRIBING INFORMATION.. 74

LIST OF ABBREVIATIONS

**AE**	Adverse event
**β-hCG**	Beta-human chorionic gonadotropin
**CCTN**	Contraceptive Clinical Trials Network
**CFR**	Code of Federal Regulations
**CRF**	Case report form
**CV**	Curriculum Vitae
**DHHS**	Department of Health and Human Services
**DSMB**	Data and Safety Monitoring Board
**eCRF**	Electronic case report form
**EDC**	Electronic data capture
**EU**	European Union
**FDA**	Food and Drug Administration
**GC/CT**	Neisseria gonorrhoeae / Chlamydia trachomatis
**GCP**	Good Clinical Practice
**HIPAA**	Health Insurance Portability and Accountability Act of 1996
**HIV/AIDS**	Human immunodeficiency virus / acquired immunodeficiency syndrome
**ICF**	Informed consent form
**ICH**	International Council for Harmonisation
**IND**	Investigational New Drug
**IRB**	Institutional Review Board
**ISO**	International Organization for Standardization
**IUD**	Intrauterine device
**LTFU**	Lost to follow-up
**NAAT**	Nucleic acid amplification test
**NICHD**	National Institute of Child Health & Human Development
**NIH**	National Institutes of Health
**OC**	Oral Contraceptive
**OHRP**	Office for Human Research Protections
**PI**	Principal Investigator
**Q1,Q2,Q3,Q4**	In the first, second, third, fourth quarter of a year
**STI/RTI**	Sexually transmitted infection / Reproductive tract infection
**SAE**	Serious adverse experience
**SCCC**	Statistical and Clinical Coordinating Center
**SOP**	Standard Operating Procedure
**TVUS**	Transvaginal ultrasound
**US**	United States
**UTI**	Urinary tract infection

PROTOCOL SYNOPSIS

**TITLE**	A multi-center, single-blind, randomized clinical trial to compare two copper IUDs: Mona Lisa NT Cu380 Mini and ParaGard
**PROTOCOL NUMBER**	CCN016
**PHASE**	III
**SPONSORS**	FHI 360<br><br>NICHD
**IND NUMBER**	132657
**IND HOLDER**	FHI 360
**FUNDING ORGANIZATIONS**	The Bill and Melinda Gates Foundation (FHI 360) and the *Eunice Kennedy Shriver* National Institute of Child Health & Human Development (NICHD) of the National Institutes of Health
**NUMBER OF SITES**	Approximately 16 sites in the United States (US)
**RATIONALE**	The only currently marketed copper intrauterine device (IUD) in the US, first approved by the FDA in 1984, consists of a plastic T-shaped frame with 380 mm^2 of copper surface. Successful use of this product has been demonstrated in parous and nulliparous women. However, nulliparous women have smaller uterine cavities, and previous research has suggested that smaller copper IUDs may cause fewer side effects in nulliparous women compared to larger products. Newer products available in Europe have slightly less copper, alternative shapes or smaller sizes. Products that are more comfortable to use will increase satisfaction, retention, and protection from unintended pregnancy.
**STUDY DESIGN**	Randomized, single-blind, phase III controlled clinical trial
**PRIMARY OBJECTIVE**	To measure contraceptive efficacy of the test product (Mona Lisa NT Cu380 Mini)

**SECONDARY OBJECTIVES**	To compare between study products:<br>• continuation rates<br>• the incidence of failed IUD insertion, uterine perforation, and IUD expulsion (complete and partial)<br>• vaginal bleeding patterns<br>• pelvic pain and dysmenorrhea<br>• the incidence of other adverse effects<br>• pain with and shortly after insertion<br>• ease of IUD insertion<br>• overall product satisfaction
**NUMBER OF SUBJECTS**	Approximately 1100 women will be randomized in a 4:1 ratio.<br>Approximately 880 will receive the test product (Mona Lisa NT Cu380 Mini) and approximately 220 will receive the control product (ParaGard).
**POPULATION**	Women aged 16-40 with anticipated need for contraception of at least one year.<br>Planned recruitment will include approximately 80% nulliparous and 20% parous women. Women 36-40 years old will comprise only 10% of the study population. We will randomize approximately 1100 eligible participants in a 4:1 ratio to two different copper IUDs: approximately 880 to Mona Lisa NT Cu380 Mini and approximately 220 to ParaGard.
**TEST PRODUCT**<br>**Dose and Route of Administration**	Mona Lisa® NT Cu380 Mini containing 380mm^2 of copper surface inserted into the uterine cavity.
**CONTROL PRODUCT**<br>**Dose and Route of Administration**	ParaGard® CuT380A containing 380mm^2 of copper surface inserted into the uterine cavity.
**COMPLIANCE**	Compliance will be documented by verification that the IUD is in situ at each visit by pelvic exam or ultrasound examination (if needed).
**DURATION OF SUBJECT PARTICIPATION AND DURATION OF STUDY**	Participants will be in the study for up to 37 months or until the IUD is removed or expelled.<br>The total duration of the study is expected to be five years: 12 months for subject recruitment, 37 months for subject follow-up, and sites will be engaged for an additional year for pre- and post-trial activities. At the Exit visit, 37 months from insertion, any subject randomized to the ParaGard treatment arm may elect to continue use of the device under

	care of the woman's primary provider. The Mona Lisa NT Cu380 Mini is to be removed at the Exit visit or 37 months following insertion.
**DOSAGE AND REGIME**	Eligible subjects will be randomly assigned to Mona Lisa NT Cu380 Mini or ParaGard in a 4:1 ratio. The IUD will be inserted by site clinician and left in place for up to 37 months (study Exit).
**SAFETY PARAMETERS**	• Occurrence of adverse events (AEs) and serious adverse events (SAEs)
**PRIMARY ENDPOINT**	• Pregnancy
**SECONDARY ENDPOINTS**	• IUD continuation at 12, 24 and 36 months after insertion<br>• Failed IUD insertion, uterine perforation, or IUD expulsion (complete and partial)<br>• Vaginal bleeding patterns<br>• Pelvic pain and dysmenorrhea<br>• Other side effects<br>• Pain with and shortly after insertion<br>• Ease of IUD insertion<br>• Overall product satisfaction
**STATISTICAL ANALYSIS**	Below is the original estimate for the 95% upper bound of the Pearl Index for the Mona Lisa Cu IUD arm if we had randomized 800 subjects to that treatment. Since this protocol has been amended to randomize approximately 880 Mona Lisa Cu IUD subjects, the estimated 95% upper bound of the Pearl Index would be slightly lower due to the larger sample size.<br><br>Randomizing 800 women to the Mona Lisa Cu IUD will provide approximately 17,700 cycles for the primary efficacy analysis among women under 36 years old at the time of enrollment, assuming 13 cycles of use per year with 84.3% evaluable cycles, an exponential discontinuation rate of 0.2 (i.e., 20% per year), and 37 months of prescribed follow-up per woman.[1] Based on this enrollment total and follow-up rate, the 95% upper bound of the Pearl Index (pregnancies per 100 women-years of follow-up) would be ~1.0 if 6 pregnancies were observed.
**PLANNED DSMB REVIEWS**	A Data and Safety Monitoring Board (DSMB) will conduct periodic reviews of subject safety and pregnancy for the study. A DSMB

[1] Based on a recent IUD study and assuming the same yearly discontinuation rate throughout 37 months, we would expect to observe approximately 7,100 cycles in year 1, 5,700 cycles in year 2 and 4,900 in year 3.

	charter will be established prior to the first DSMB meeting in order to detail the frequency, timing and scope of the reviews.<br><br>In addition, the DSMB will review an interim analysis.

NICHD/FHI 360

Clinical Research Protocol

A multi-center, single-blind, randomized clinical trial to compare two copper IUDs: Mona Lisa NT Cu380 Mini and ParaGard

1 BACKGROUND AND RATIONALE

The only copper intrauterine device (IUD) currently marketed in the United States is ParaGard®.[1] It was approved for use in 1984 and first marketed in 1988; millions of US women have relied on its high safety and high efficacy for pregnancy protection. Copper IUDs are unique for having three key attributes: being non-hormonal, long-acting, and reversible. For many women, these features make it an indispensable option.

Unfortunately, copper IUD users can experience increased bleeding and cramping; these side effects can lead up to 20% of women to discontinue use within the first year.[2-5] Nulliparous women typically experience higher rates of side effects compared to parous women.[6] Recent findings from the largest contemporary cohort of copper IUD users in the USA confirm that nulliparous women have a higher incidence of removals for side effects compared to parous women[7]. However, the current product still provides tremendous benefit to a majority of nulliparous users.

Higher side effect and removal rates among nulliparous women may be due to discrepancies between the size/shape of the current product and the uterine cavity. ParaGard is the largest T-shaped product in the world (measuring 32mm x 36 mm width and length, respectively). On average, nulliparous women have uterine cavities that are smaller than those of parous women. A recent study estimated a mean cavity volume of 57cm^3 for nulliparous women versus 82cm^3 and 105cm^3 for women with one and more than one live birth, respectively.[8] Width of the cavity averaged 27mm among nulliparous women to 32mm among women with more than one live birth.

Previous research in nulliparous populations suggests that smaller copper IUDs may be more acceptable than similarly-shaped, standard-sized products.[6,9,10] The only randomized trial (of nulliparous women) comparing a ParaGard-sized product to a smaller, T-shaped product (measuring 23mm x 29mm), found that the smaller product had continuation rates that were more than two times higher than the larger product.[11]

The American Academy of Pediatrics[12] and the American College of Obstetricians and Gynecologists[13] state that IUDs should be a first-line option for nulliparous women. Newer and smaller copper IUDs used in Europe may be as effective as ParaGard and may cause fewer side effects. This study aims to evaluate the performance of a smaller, European-approved product compared with ParaGard in a predominately nulliparous population.

The National Institute of Child Health and Human Development (NICHD) has a mission to develop safe and effective contraceptives for women, including obese women. Obesity is the number one public health issue facing the US population and is an independent risk for venous

thromboembolism (VTE). Therefore, there is a public health need to develop effective estrogen-free contraception that does not increase the risk of VTE. A copper IUD that contains no hormonal contraceptives may be safer than a hormonal method, and therefore could have a direct benefit to obese subjects.

2 STUDY PRODUCTS

2.1 Brief Product Descriptions – Structure and Regulatory Status

This study involves two different copper IUDs: ParaGard (the control product) and Mona Lisa NT Cu380 Mini (test product). Brief descriptions of each are provided.

2.1.1 ParaGard (control)

The ParaGard T 380A Intrauterine Copper Contraceptive (NDA 18-680/S-060) was approved by the US Food and Drug Administration (FDA) in 1984 and was first marketed in the US in 1988. ParaGard is approved for intrauterine contraception for up to 10 years. The pregnancy rate in clinical studies has been less than 1 pregnancy per 100 women each year.[12] The T-framed product measures 32mm across the arms by 36mm in height, and is made of polyethylene with barium sulfate to aid in radiologic detection of the device. Copper is added to the plastic frame to enhance efficacy. A total of 380mm^2 of copper surface is on the product (copper collars on each arm and a copper wire wound around the vertical stem of the T-frame). This product is currently marketed in the USA by Teva Pharmaceuticals.

2.1.2 Mona Lisa NT Cu380 Mini (test product)

The Mona Lisa NT Cu380 Mini IUD is a T-shaped product that measures 24 mm (arm span) x 30 mm in height (about 20% smaller than the T 380A). Like the T380A, the Mona Lisa NT Cu380 Mini is made of polyethylene, has 380 mm^2 of copper surface and contains barium sulfate. The Mona Lisa NT Cu380 Mini was first approved in Belgium in 2014 and is indicated for contraception for a 5-year duration. It is currently marketed in Canada, Germany, France, and six other European countries.

The Mona Lisa NT Cu380 Mini is manufactured by Mona Lisa N.V., Graaf de Theuxlaan 25, bus 2, 3550 Heusden-Zolder, Belgium. The company also manufactures the NT Cu380 Standard that is about the same size as the T 380A. The product insert states that the Mona Lisa NT Cu380 Mini is “designed for women with a uterine cavity depth of less than 7 cm”, while the larger version is “designed for women with a uterine cavity depth of 6-9 cm.”

Specific pharmacologic and toxicology studies have not been conducted on the NT Cu380 Mini; however, studies have been conducted for the larger NT Cu 380 Standard, which comprises identical materials and copper surface area. There are no clinical trial data for the Mona Lisa NT Cu380 Mini or for the NT Standard.

2.2 Overview of Non-Clinical Studies

In this section, only information on the test product is presented. Refer to the package insert (Appendix 4) for information on ParaGard.

2.2.1 Mona Lisa NT Cu380 Mini

The supporting nonclinical biocompatibility and toxicity studies were carried out on the NT Standard, which is the predecessor to the Mona Lisa NT Cu380 Mini. The NT Standard uses the same manufacturing materials and processes and has the same amount of copper, and is therefore equivalent in essential characteristics to the Mona Lisa NT Cu380 Mini. Since the NT Standard is larger than the Mini, the data may be considered "worst case" or equivalent in essential characteristics; the relevancy of all data will be supported by a risk analysis according to ISO 14971:2007 Medical devices—Application of risk management to medical devices.

The biocompatibility tests on the NT Standard were completed in 2004, however, no GLP certification was provided. The tests included cytotoxicity, sensitization, in vivo irritation in rabbits, pyrogenicity, and systemic acute toxicity. All of the tests were considered negative based on standards set by ISO 10993.

In 2013, a GLP lab did systemic injection tests, intracutaneous injection tests, Kligman maximization test, reverse mutation assay tests, and cytotoxicity tests with other Mona Lisa copper IUDs. The tested IUDs contained the same frame and copper materials as the Mona Lisa NT Cu380 Mini (the only difference in materials was polyethylene threads instead of nylon threads). All the tests were considered negative based on standards set by ISO 10993.

Genotoxicity testing was not done on the Mona Lisa NT products. However, tests were done on other copper IUD designs that use the same component materials as the Mona Lisa NT products. In tests on these other products, involving *S. typhimurium and E. coli*, no mutagenic concerns were discovered.

The summary of findings from nonclinical studies is presented in the separate Investigator's Brochure.

2.3 Overview of Clinical Studies

In this section, only information on the test product is presented. Refer to the prescribing information (Appendix 3 and 4) for information on ParaGard and Mona Lisa NT Cu380 Mini respectively.

No clinical studies on the Mona Lisa NT Cu380 Mini have been conducted. The Mona Lisa NT Cu380 Mini has a CE Mark certificate, having met regulatory requirements of the EU Medical Devices Directive (93/42/EEC) for a Class III product, and is marketed within the EU and European Free Trade Association for intrauterine contraception for up to 5 years.

Since 2014 and through August 2016, over 40,000 units of the Mona Lisa NT Cu380 Mini were sold in Europe with the vast majority (over 36,000 units) distributed to France and 1,200 units to Canada. During this time period, no reportable incidents have come to the European Commission's medical device vigilance voluntary reporting system. No advisories, warnings, or safety alerts have been issued on this product since it was first marketed.

3 STUDY OBJECTIVES

3.1 Primary Objective

To measure contraceptive efficacy of the test product (Mona Lisa NT Cu380 Mini)

3.2 Secondary Objectives

To compare between test products:
- continuation rates of the products
- the incidence of failed IUD insertion, uterine perforation, and IUD expulsion (complete and partial)
- vaginal bleeding patterns
- pelvic pain and dysmenorrhea
- the incidence of other adverse effects
- pain with and shortly after insertion
- ease of IUD insertion
- overall product satisfaction

4 STUDY DESIGN

4.1 Study Overview

This will be a multi-site, participant-blinded, randomized clinical trial. We will randomize approximately 1100 eligible participants in a 4:1 ratio to two different copper IUDs: approximately 880 to Mona Lisa NT Cu380 Mini and approximately 220 to ParaGard. Planned recruitment will include approximately 80% nulliparous and 20% parous women. A parous woman will be defined as having had a delivery at 6 months/24 weeks.

The study will assign subjects to the treatments in random order. Each woman will attend a baseline visit, followed by visits at 6 weeks, 3 months, 6 months, 12 months, 24 months and 37 months, with telephones calls at 9, 18 and 30 months. Each subject will participate for up to 37 months (or earlier if the product is removed or expelled). Subjects will use a home pregnancy test 17 days after Exit Visit procedures or any time she suspects that she may be pregnant, and report the results to their respective sites via telephone.

At the Exit visit, 37 months from insertion, subjects using ParaGard may elect to continue use under the care of the woman's primary provider. The Mona Lisa NT Cu380 Mini will be removed at the Exit visit or 37 months following insertion. The length of elective continued use for Mona Lisa NT Cu380 Mini may be adjusted to allow continued use for years 4 and 5 once incidence of key endpoints is evaluated through interim analysis of the currently planned study. A separate consent document and other study requirements would still be required if such a decision is made.

We will conduct this study according to CFR Title 21, CFR Title 45, Part 46, Good Clinical Practices (GCP) and International Council for Harmonisation (ICH) Guidelines under an IND Application. All principles of the 2013 revision of the Declaration of Helsinki will be followed.

4.2 Study Duration

The total duration of the study for each participant is expected to be approximately 39 months: including screening and enrollment (up to 60 days to meet enrollment criteria), 37 months of participation, and a follow up telephone call 17 days post-removal. After enrollment, subject visits occur at 6 weeks, 3 months, 6 months, 12 months, 24 months, with telephone calls at 9, 18, and 30 months and seen again at 37 months for their final visit. Subjects will use a home pregnancy test 17 days post-removal of the IUD or Exit Visit procedures, whichever occurs first, and will be called by the site for the result and for safety follow-up.

Subject recruitment began in Q2 (in the second quarter of) 2017 and is expected to continue through Q1 2019. However, if the enrollment rate declines or the target enrollment is not reached, the enrollment period may be extended beyond this date. If this enrollment timeline is met, all subjects should finish active treatment by approximately the end of Q1 2022. The total duration of the study will be approximately 57 months for each study site including pre- and post- trial activities. The end of the study will occur when the last subject to be enrolled has completed her post-removal pregnancy test telephone call.

Total duration of the project is expected to be approximately five and a half years. Preliminary results of the study are expected to be available Q3 of 2022 based on the current study plan.

5 PRINCIPAL INVESTIGATORS AND RESEARCH SITES

The National Institute of Child Health and Human Development (NICHD) will conduct the study using 11 sites from its Contraceptive Clinical Trials Network (CCTN), and FHI 360 will conduct the study at five additional sites. A complete list of Principal Investigators, Sub-Investigators and Coordinators will be maintained with the trial documentation. Listed here are the investigational research sites conducting this study.

5.1 FHI 360 Sites

Courtney Schreiber, MD, MPH: Perelman School of Medicine at the University of Pennsylvania

Beatrice A. Chen, MD, MPH: University of Pittsburgh

Anita Nelson, MD: Essential Access Health

Alisa Goldberg, MD: Planned Parenthood League of Massachusetts

Ila Dayananda, MD: Planned Parenthood New York City

5.2 NICHD Sites

David Archer, MD: Eastern Virginia Medical School

Anne Burke, MD: Johns Hopkins University

Paula Castaño, MD, MPH: Columbia University

Mitchell Creinin, MD: University of California, Davis

Philip Darney, MD, MSc: University of California, San Francisco

Jeffrey Jensen, MD, MPH: Oregon Health & Science University

Subsite: Bliss Kaneshiro, MD, MPH: University of Hawaii

Stephanie Teal, MD, MPH: University of Colorado Denver

Michael A. Thomas, MD: University of Cincinnati

David Turok, MD: University of Utah

Katharine White, MD, MPH: Boston Medical Center Corporation

6 CRITERIA FOR EVALUATION

6.1 Primary Endpoint

The primary endpoint for this study is pregnancy (contraceptive method failure). All pregnancies will be dated by a combination of available clinical data according to the American College of Obstetricians and Gynecologists criteria for pregnancy dating. We will review all pregnancies to estimate conception dates, and assess carefully whether pregnancies that are diagnosed soon after IUD insertion or removal should be classified as method failures.

6.2 Secondary Endpoints

- IUD continuation
- Failed IUD insertion, uterine perforation, or IUD expulsion (complete and partial)
- Vaginal bleeding patterns
- Pelvic pain and dysmenorrhea
- Other side effects
- Pain with and shortly after insertion
- Ease of IUD insertion
- Overall product satisfaction

6.3 Safety Evaluations

The study will not require routine laboratory monitoring for safety, given that these are non-hormonal products. At each study visit site staff will collect information on adverse events (AEs) and serious adverse events (SAEs).

7 SUBJECT SELECTION

7.1 Study Population

Approximately 16 clinical sites in the US will enroll reproductive-aged women participants from family planning and other clinics providing gynecologic care and reproductive health services. Referrals from previous studies are acceptable. This study will be conducted in the US by the National Institute of Child Health and Human Development (NICHD) in its Contraceptive Clinical Trials Network (CCTN) at 11 sites and by FHI 360 at 5 sites. This study will be listed on the trial registration website ClinicalTrials.gov. Subject selection will occur based on the following inclusion and exclusion criteria.

7.2 Inclusion Criteria

- 16-40 years
    - 16 and 17 year olds, where permissible by state regulations and local Institutional Review Board (IRB) approval
- Sexually active, anticipating at least one act of vaginal intercourse per menstrual cycle with a male partner and at risk for pregnancy
- seeking contraception, and willing to use the study IUD as the only contraception method
- willing to be randomized to one of the two copper IUDs
- has an intact uterus and at least one ovary
- has a history of regular menstrual cycles; defined as occurring every 21-35 days when not using hormones, and with a variation of typical cycle length of no more than 5 days
- able and willing to provide written informed consent
- agrees to follow all study requirements
- not currently pregnant or at risk for luteal phase pregnancy based on history of unprotected intercourse

7.3 Exclusion Criteria

- abnormal Pap requiring treatment after enrollment
- known human immunodeficiency virus / acquired immunodeficiency syndrome (HIV/AIDS) infection
- intending to become pregnant in the 37 months after enrollment
- known infertility
- history of allergy or sensitivity to copper
- previous tubal sterilization
- has received an injectable contraceptive in the last 9 months and has not resumed regular menstrual cycles (as evidenced by 2 spontaneous menses)
- within 30 days of administration of mifepristone and/or misoprostol for medical abortion or for miscarriage management
- within 30 days of first, second, or third trimester abortion or miscarriage (note: potential abortion/miscarriage participants can be screened and return after 30 days for randomization and IUD insertion)
- within 30 days of delivery (for parous population)
- breastfeeding or recently breastfeeding women unless two consecutive normal menstrual periods have occurred after delivery and prior to enrollment.
- wants to use a copper IUD for emergency contraception
- has previously participated in the study
- participated in another clinical trial involving an investigational product within the last 30 days (before screening) or planning to participate in another clinical trial involving an intervention or treatment during this study
- not living in the catchment area of the study site or planning to move from the area within the year (unless known to be moving to the catchment area of another study site)

- known or suspected current alcohol or drug abuse
- planning to undergo major surgery during study participation
- current need for use of exogenous hormones or therapeutic anticoagulants (Note: subjects who start a therapeutic anticoagulant after enrolment will be allowed to continue in the study.)
- at high risk for sexually-transmitted infections or pelvic infection
- anticipated need for regular condom use (refer to Section 8.1).
- has any condition (social or medical) which in the opinion of the Investigator would make study participation unsafe or complicate data interpretation
- Reported medical contraindications (Medical Eligibility Criteria category 3 or 4)[14] to copper IUDs, including:
    - suspicious unexplained vaginal bleeding
    - known cervical cancer
    - known endometrial cancer
    - known Wilson's disease
    - Confirmed gestational trophoblastic disease with persistently elevated beta-hCG levels or malignant disease, with evidence or suspicion of intrauterine disease
    - anatomic abnormalities with distorted uterine cavity
    - current pelvic inflammatory disease (PID)
    - pelvic tuberculosis
    - immediately post-septic abortion or puerperal sepsis
    - current known purulent cervicitis or chlamydial infection or gonorrhea; Note: to enroll, there must be no obvious signs of infection at the time of enrollment based on pelvic exam. If lab results come back for positive infection after enrollment, treatment should be provided but the IUD can be left in place.
    - complicated solid organ transplantation
    - systemic lupus erythematosus with severe thrombocytopenia

7.4 Allowable Criteria

- has been using oral contraceptives, vaginal ring, or patch and is discontinuing the product to be enrolled
- has been using a contraceptive implant or IUD, but wishes to have the device removed prior to enrollment

7.5 Number of Subjects

The study is expected to enroll approximately 1100 female subjects randomized to one of the two IUDs.

8 CONCURRENT MEDICATIONS AND TREATMENTS

8.1 Allowed Medications and Treatments

Recording of all concomitant treatments will occur on the subject's source and electronic case report forms (eCRF), including the name of the drug, start and stop dates and reason for use from the time the informed consent form (ICF) is signed through discontinuation from the trial. Any other investigational product should be stopped at least 30 days before the start of screening for this trial. Other medications for the treatment of inter-current medical conditions will be permitted and recorded as detailed above unless identified on the exclusionary list of medications.

Condoms are allowed for protection from sexually transmitted infection (STI). However, anticipated regular use of condoms is exclusionary. Regular use of condoms is defined as an anticipated need of at least one condom per month after enrollment.

If a subject suspects that her IUD is not in place, she should return to the site as soon as possible for confirmation of IUD placement. In the meantime, she should use condoms during intercourse until she is evaluated by the site.

8.2 Prohibited Medications and Treatments

Concomitant medications that should not be used during the study include:

- Exogenous hormones, including hormonal contraceptives, except if needed for medical reasons and used in a single cycle during the study
- Therapeutic anticoagulants (at enrollment only)

9 STUDY TREATMENTS

9.1 Method of Assigning Subjects to Treatment Groups

The study will randomly assign approximately 1100 eligible participants to Mona Lisa NT Cu380 Mini or ParaGard in a 4:1 ratio using a SAS-based computer-generated randomization scheme. Randomization will be stratified by parity (nulliparous and parous will be the stratification categories with a goal of enrolling 80% nulliparous). Enrollment will be tracked to ensure approximately 10% of women are 36-40 years old.

The randomization listing will be uploaded to the HD EDC system for proper stratified randomization. The IUDs for each site will be provided in bulk and clinicians will pull the product from the existing stock based on the product identified in the EDC system at time of randomization.

Health Decisions will hold the randomization listing in a secure limited access location for the duration of the study.

Sites will enter the kit and randomization number provided from the EDC system on their IP dispensing log for each subject. The subject identification number assigned to subjects during the screening visit will identify them for the duration of the study.

9.2 Blinding (participant only)

The identity of the assigned IUD will not be shared with participant from the time of insertion through the Exit/37-month study visit. However, the clinicians inserting the product and clinicians conducting follow-up exams will know which product was assigned. Clinic staff completing other assessments should remain blinded as much as possible.

Sites will ensure single-blind administration of study treatments using the following study procedures:

- Once the product assignment is determined, study staff will prepare the IUD and supplies outside the view of the participant
- The study staff will pull the IUD from the stock and place all identifying packing information out of the sight of the subject (Note: all packaging materials should be retained in a secure location for examination and accountability by the monitor)
- If necessary, an overlay will cover the tray (or insertion supplies when not used on a tray) to prevent inadvertent unblinding to the study participant.

When the participant returns for follow-up, the participant blinding is to be maintained. The site will break the blind for the study participant on completion of her study participation at the Exit visit after all IUD assessments have been performed. During the study, the participant blind may be broken **only** in emergencies when the patient or treating physician must know which product was received. When possible, the Investigator should discuss the emergency with the Medical Monitor prior to unblinding. As outlined in Section 12.1, subjects should be instructed to contact the study site to report any untoward medical occurrence that they experience. If health care is received elsewhere, for any reason, the participant should inform the attending clinician of their study participation request that no details of their IUD be shared.

9.3 Formulation of Test and Control Products

The products used in this study are the ones that are marketed in the USA and in Europe.

9.3.1 Formulation of Test and Control Products

Table 01. Formulation and the Test IUDs

	**Control Product**	**Test Product**
	ParaGard	Mona Lisa NT Cu380 Mini
Manufacturer	Teva Pharmaceutical, USA	Mona Lisa, N.V.<br>Belgium
Active ingredient	Copper<br>380mm^2 surface area	Copper<br>380mm^2 surface area
Copper purity	>99.95%	>99.95%
Frame	Polymer	Polymer
Free state shape	“T” shape	“T” shape

	Control Product	Test Product
Frame dimensions	32mm x 36mm	24mm x 30mm

9.3.2 Packaging and Labeling

All labeling of the study product will be prepared in accordance with 21CFR, Section 812.5. Study product will be supplied in the original manufacturer packaging containing a single use IUD with a study label applied on the original packaging for study purposes. The study label will provide the study specific information as follows:

- Protocol Identification: CCN016
- Sponsor Identification: NICHD/FHI 360
- Kit Number (preprinted/space to record)
- Subject ID (space to record)
- Subject Initials (space to record)
- Date Dispensed (space to record)
- FDA Caution Statement: CAUTION -- Investigational drug. Limited by Federal (or United States) law to investigational use.
- Distributor: FHI 360, 359 Blackwell Street, Suite 200, Durham, NC 27701
- Directions: Use as Directed in CCN016 Protocol.
- Storage: Controlled Room Temperature (15 to 30ºC (59 to 86ºF))
- Keep Out of Reach of Children

9.4 Provision of Study Product to Participants

At the study site, the Principal Investigator (or designee) will insert the IUD into the participant's uterus during the enrollment visit. If insertion is not successful on the first attempt, a second attempt with the same product may be made during the enrollment visit only. If second attempt is unsuccessful then the subject will be discontinued.

Once successfully inserted, the IUD will remain in place for up to 37 months for current study purposes unless product expulsion occurs, safety concerns arise or the subject chooses to have the IUD removed prior to completion of 37 months of use for any reason. No replacement of study IUDs will occur should product expulsion or accidental removal occur. If expulsion or accidental removal occurs prior to 37 months of continuous use, subjects should contact the study site immediately to be scheduled for an Exit Visit.

9.4.1 Dosage Regimen

Subjects will be enrolled and randomized to receive one of the copper IUDs being studied. The single IUD will be inserted in the uterus by delegated study staff at a subject's enrollment visit.

9.4.2 Dispensing

The Principal Investigators (or designees) have authority to retrieve the IUDs from the temperature controlled, secure limited access stored supply stock.

Prior to dispensing, the Principal Investigator or designee will record the site number, subject's identification number, initials, and date dispensed on the overlay packaging. Upon dispensing, the original manufacture packaging should be replaced within the overlay packaging for monitoring accountability purposes.

The Principal Investigator (or designee) will record the randomization number, subject identification number, subject initials, and date dispensed on the accountability log.

9.4.3 Administration Instructions

This is a single-blinded study; the participant should not see the assigned IUD or the manufacturer's product packaging. Precautions should be taken to prevent subjects from seeing the manufacturer's product packaging, including but not limited to covering the IUD and insertion supplies with a sterile overlay to prevent inadvertent unblinding.

Each IUD has an FDA- or European-approved product label that contains insertion instructions. Training will occur for Investigators on proper insertion technique for the test product. Investigators can also refer to the product label and Investigator's Brochure for additional information.

All pre-insertion clinical assessments are the same for all IUDs as described in Sections 11.1.1 Screening Procedures and 11.1.2 Enrollment Procedures.

9.5 Supply of Study Product at the Site

Health Decisions will oversee the shipment of Study Product to the investigational sites. Shipment of the initial study product will occur after site authorization (i.e., all required regulatory documentation has been received by the Sponsor, IRB approval has been received and a contract has been executed). Once a site request for resupply is received, shipment of subsequent study product will occur.

9.5.1 Storage

Study product should be stored in a dry place at the study site at controlled room temperature, 15 to 30ºC (59 to 86ºF) under controlled and secured conditions (in a locked storage area away from sunlight). A temperature log for the investigational product storage area must be maintained for the duration of the study. If the temperature of the study product storage area exceeds or falls below the allowable range, this should be reported to the Sponsor or designee and captured as a deviation.

9.6 Study Product Accountability

An accurate and current accounting of the dispensing, insertion, and removal of study product for each subject will be maintained on an ongoing basis by a member of the study site staff. Recording of information regarding study product disposition will occur on the Investigational Product Accountability Log by Investigator (or designee). The study monitor will verify these documents and product packaging on a regular basis throughout the course of the study. Refer to Section 9.4.2 for some of the information that dispensing staff will capture on the Accountability Log. After documented accountability has been completed for a subject by the monitor, the site may dispose of the used study product and/or product overlay packaging per Section 9.8.

9.7 Measures of Treatment Compliance

Compliance with treatment is a key endpoint of the study; this status is documented by verifying that the IUD is in situ. To verify the presence of the IUD, the clinician will check for the IUD string at each study visit (refer to **Appendix 2** for confirmation process). If the IUD string is not visible or palpable on examination, or if there is clinical suspicion of expulsion, clinicians will proceed with ultrasound and x-ray examination (if appropriate) to verify that the IUD is in position. Should IUD be removed outside of the site, the Investigators should request release of medical records by subject upon notification if removal was completed by a non-study medical provider or proceed with verification steps in Appendix 2 to confirm removal if completed by the subject. For this study, we will use the following definitions:

1. Complete expulsion: the IUD is completely outside of the uterus and cervix, as evidence by seeing the IUD outside the uterus or complete absence of the IUD on ultrasound examination with no evidence of an intraabdominal IUD by X-ray;
2. Partial expulsion: visualization or palpation of any portion of an IUD (other than strings) at the cervical os, or ultrasound identification of any part of the IUD in the cervical canal, regardless of symptoms.

9.8 Study Product Disposal/Return

At the end of the study, the monitor will complete a final accountability of all clinical supplies, including remaining unused product and used product overlay packaging. Remaining used (e.g., removed or expelled) study product destruction will occur on-site following site-specific destruction standard operating procedures once monitor accountability has been completed. Guidance for remaining unused study product return or disposal will be provided by the Sponsors at the end of the study.

10 STUDY PROCEDURES AND GUIDELINES

10.1 Investigator's Meeting and Protocol Training

Prior to initiating recruitment of participants, an Investigator's Meeting will be held for the following purposes:

- To train Investigators on proper insertion of the test product
- To train Investigators on the protocol specifics and study manuals
- To document training
- To train on other study specific requirements

10.2 Clinical Assessments and Procedures

A Schedule of Assessments representing the required testing procedures to be performed for the duration of the study is diagrammed in Appendix 1.

Prior to conducting any study-related activities, written informed consent and the Health Insurance Portability and Accountability Act (HIPAA) authorization must be signed and dated by the subject or subject's legal representative. If appropriate, assent must also be obtained prior to conducting any study-related activities.

10.2.1 Medical, Demographic, and Reproductive Health History

At the screening visit, study staff will ask women about their medical and gynecologic history, including specific questions about any medical problems or current medications that may be contraindications to the use of any of the study methods. Women will also be asked about their past use of contraceptive methods as relevant and details about their previous pregnancies, if any. General subject demographic information (i.e. race, ethnicity, level of education, etc.) will also be collected at screening.

10.2.2 Vital signs and Pelvic Exam

Trained research staff will collect vital signs on all potential participants at screening to evaluate for contraindications to the study methods. These exams will include measurement of blood pressure, height, and weight, and a pelvic exam, including speculum exam. A bimanual exam will be done to assess for the presence of any uterine or cervical anomalies, possible pelvic infection, or other abnormal findings such as an adnexal mass that would preclude IUD use. An assessment of uterine cavity length also will be completed by sound or pipelle while speculum is still in place after the subject is randomized but prior to IUD insertion. Any suspected reproductive tract infections (RTI) identified on physical exam will be diagnosed and treated according to local standards of care. Enrollment will be deferred pending treatment. In addition, treatment will be initiated and/or adjusted as appropriate based on laboratory results when available and include treatment of sexual partners when indicated. If an infection is suspected or confirmed, treatment according to the site’s standard of care is indicated. Once treatment is complete, the subject may return for continued screening and may be enrolled within a month of the start of screening if there is no obvious sign of infection. If no infection is suspected at time of screening, subjects may enroll the same day.

10.2.2.1 Suspected gonorrhoea, chlamydia, or Pelvic infection

Clinicians diagnosing suspected gonorrhoea, chlamydia or pelvic infection on the basis of exam will postpone subject enrollment and prescribe appropriate treatment. In addition, treatment will be initiated and/or adjusted as appropriate based on laboratory results when available and include treatment of sexual partners when indicated. Once treatment is complete, the subject may return for continued screening and may be enrolled within a month of the start of screening if there is no obvious sign of infection per clinician judgement.

10.2.3 Ultrasound examination

Ultrasound examinations pre or post-enrollment are not required and should not be standard. Clinician judgement should be used to determine whether, when, and what type of ultrasound examination is needed. Potential reasons include, but are not limited to:

- difficult insertion
- suspected perforation
- suspected expulsion
- pregnancy

Steps should be taken to ensure that the participant does not look at the ultrasound screen and that pictures are not shown to the subject to ensure subject blinding is maintained.

10.2.4 Pap Test

A screening Pap test will be completed for subjects at screening if potential participant is not in compliance with normal standard of care guidelines regarding Pap testing. ACOG guidelines should be used for current Pap test standard of care requirements. Participants will need to provide results of previous Pap to confirm results are within ACOG Pap test standard of care requirements for Pap testing not completed by the site. Participant pap test results, as required per ACOG guidelines, are required prior to the participant being enrolled and randomized into the study. When scheduling Screening visits, sites should request subject bring Pap results with them to the Screening visit. If additional testing or treatment is required based on Pap results, this should be completed prior to enrollment.

10.2.5 Laboratory Assessments

Laboratory evaluations:

See Visit Procedures section 11.

10.2.5.1 STI screening

During screening visits, at the time of the pelvic exam, all participants will be screened for gonorrhoea and chlamydia using nucleic acid amplification testing of urine, vaginal, or endocervical swabs. Asymptomatic participants do not need to wait for gonorrhoea and chlamydia results to receive their assigned IUD.

For a few asymptomatic women, IUD insertion might occur before results of a positive gonorrhoea or chlamydia test are available. For these participants, treatment will commence as soon as possible once results of a positive test are received. IUDs should not be removed unless severe pelvic infection is suspected.

10.2.5.2 Urine pregnancy testing

Participants will undergo urine pregnancy testing at screening and enrollment (if enrollment occurs on a separate day from screening). A negative test is required to proceed with possible IUD placement. In addition, urine pregnancy tests will be administered during follow-up visits. Each facility will use the test kits and specific laboratories normally used for routine services at the facility's local laboratory.

> Note: if urine pregnancy test is positive at screening, no further action is required and subject will become a screen failure. If positive urine pregnancy test report is based on home pregnancy test result post enrollment, subject should be scheduled to come in for an Unscheduled visit to have quantitative blood pregnancy test performed. If confirmatory quantitative blood pregnancy test comes back negative, then subject will be allowed to stay in the study.

10.2.6 Specimen collection and Processing

Each site will establish standard operating procedures, including specimen chain of custody and quality control procedures, for all protocol-specified laboratory tests before study initiation as

per local laboratory requirements for sample processing in accordance with standards of good clinical and laboratory practice.

10.3 Subject Assessments

10.3.1 Follow Up Form

Administration of a follow-up form will occur at Visit 2, Visit 3, Visit 4, Call 1, Visit 5, Call 2, Visit 6, Call 3 and Exit for subjects to answer questions about the desire to continue use of the IUD, IUD acceptability, last menstrual cycle, vaginal bleeding, pelvic pain, and use of alternative contraception since the preceding visit.

10.3.2 Daily Diary

For the first 12 months of use, participants will maintain a daily diary to record bleeding/spotting. Each form will collect one cycle of information consisting of 28 days of daily records. The form will also be used to record the use of any additional contraceptives (e.g. condoms) and coitus within that full 28-day cycle. Site staff will review responses carefully to ensure there are no questions about responses provided by the subject. The information from the daily diaries will be entered in the corresponding CRFs. Each site will retain originals of the completed diaries as source documents and certified copies will be uploaded to the study document repository or EDC at Health Decisions.

10.3.3 Other Contraceptive Use

In the second and third years, participants will record cycle-to-cycle use of any other contraceptive methods. The forms will be used to capture information on other contraceptives used for the six month period between site visits. The information from the forms will be entered in the corresponding CRFs during Visit 6, and Exit. At Calls 2 and Call 3 the site will ask information relative to other contraceptives used since the previous visit in order to prompt subjects to continue completing the forms; however, when the forms are returned at the clinic visits the form responses will be the primary source which will be used for data entry rather than information discussed during the calls that sites may have recorded in the subject's source. Each site will retain originals of the completed forms as source documents and certified copies will be uploaded to the study document repository or EDC at Health Decisions.

10.3.4 Post Placement Pain Forms

During the enrollment visit, a Clinic Post Placement Pain Form will be completed by the subject to record her baseline pain measurement after speculum placement and then pain measurements immediately and ten minutes after IUD placement. During the Exit Visit, the subject will complete a measurement of pain immediately after IUD removal on the bottom of the Clinic Post Placement Pain Form.

A separate Home Post Placement Pain Form will be sent home so subjects can record their maximum daily pain for 6 days following IUD placement. Also on this form, the subject will provide information on satisfaction with the IUD and use of pain medications. This form will be turned in at the 6 week visit.

11 EVALUATIONS BY VISIT

11.1 Screening and Enrollment/IUD Insertion

Visit 1 includes screening, enrollment, and IUD insertion. Screening and enrollment can occur on the same day provided the potential participant meets eligibility criteria at the time of screening, and is not at risk for luteal phase pregnancy based on history of unprotected intercourse within her current menstrual cycle. If the participant is at risk for pregnancy in the current cycle or is suspected to have gonorrhea, chlamydia, or pelvic infection, enrollment should be deferred. If the subject is under 21 years of age, no Pap is required per ACOG guidelines. If the potential participant is unable to provide results of previous Pap, defer enrollment until Pap results are available confirming treatment would not be required.

11.1.1 Screening Procedures

At the screening visit, study staff will conduct administrative and regulatory procedures (including obtaining written informed consent, providing contraceptive counselling, obtaining medical and reproductive health history, performing pelvic examinations, and collecting laboratory specimens for testing). Potential participants will provide written consent before any screening procedures are initiated. If needed, sites will refer women who are not eligible for the study for further care, if relevant services are not available at the research site or clinic. If a subject is a screen failure on her first study participation attempt, she may be rescreened one time per the Investigator’s discretion.

Table 02. Screening Visit Procedures

Component	Procedures
**Administrative and Regulatory**	• Obtain written informed consent, HIPAA Authorization and Assent (if applicable)<br>• Assign Subject Identification number<br>• Assess eligibility<br>• Collect participant’s contact information (e.g., cell phone, home phone, email, etc.). Note: If the subject has a cell phone, study staff should call the participant’s cell phone number at least one time prior to enrollment to ensure number correctly connects to the subject.<br>• File paper forms in a secure location<br>• Provide reimbursement<br>• Schedule next visit
**Contraceptive Counselling**	• Describe contraceptive options, study IUDs, and answer potential participant questions
**Clinical**	• Collect demographic information<br>• Obtain medical, gynecologic, menstrual, contraceptive, and reproductive history

		• Check vital signs and conduct pelvic examination<br>• Collect concomitant medication information<br>• Collect Pap test if subject is unable to provide results within ACOG standard of care guidelines
**Laboratory**	Urine	• Test urine for pregnancy
	STI	• Test endocervical or vaginal swabs or urine (per clinic testing standard) for *Neisseria gonorrhoeae* and *Chlamydia trachomatis* (GC/CT) to complete a Nucleic Acid Amplification Test (NAAT) (per clinic testing standard) and treat clinically suspected gonorrhoea, chlamydia, or pelvic infection per standard of care

11.1.2 Visit 1 (Enrollment Procedures and IUD Insertion)

Potential participants will usually proceed to enrollment procedures immediately after successful screening (i.e., screening and enrollment visits can occur on the same day).

The protocol allows same-day enrollment and IUD insertion in these potential participants:

- Women not using hormonal contraception who are in the first 7 days of the menstrual cycle,
- Women who have been correctly and consistently using a reliable hormonal method of contraception before screening (OCs, patch, ring, implants) once these methods are stopped/removed,
- Women who have been using any type of IUD (same-day removal and study enrollment is acceptable),
- Women who are ≥ 30 days post uncomplicated medical abortion who have been consistently using a reliable hormonal method of contraception before screening (OCs, patch, ring) or have not had vaginal intercourse since pregnancy termination,
- Women who are ≥ 30 days post first, second or third trimester surgical abortion who have been consistently using a reliable hormonal method of contraception before screening (OCs, patch, ring) or have not had vaginal intercourse since pregnancy termination,
- Non-breastfeeding women who are ≥ 30 days post-delivery who have been consistently using a reliable hormonal method of contraception before screening (OCs, patch, ring) or have not had vaginal intercourse since pregnancy termination.

If enrollment cannot proceed for any reason, including a contraindicated infection or immediately post abortion/delivery, the potential participant should return no later than 60 days from the start of screening to be enrolled. If the potential participant was treated for a sexually transmitted infection, treatment should be complete and there should be no signs of active infection prior to enrollment. If the potential participant appears to have been re-infected, she would be considered a screen failure. If the potential participant was screened less than 30 days post-abortion or delivery, confirmation of eligibility should be completed prior to randomization including review for any new history, review of concomitant medications and urine pregnancy check. Review of cases that require a screening period longer than 60 days will occur on a case by case basis through a submitted waiver. If the enrollment visit occurs separately from screening, sites will reconfirm study eligibility prior to enrollment, including pregnancy testing. Study staff will collect adverse events (AEs) and any new or changed concomitant medications. Sites will randomize eligible women (at which point a woman will be considered enrolled) and women will receive one of the two study IUDs. Once enrolled, reimbursement for the women's time and scheduling of their 6-week contraceptive follow-up visit will occur.

Table 03: Enrollment Visit Procedures

**Component**	**Procedures**
**Administrative and Regulatory**	• Randomize subject in EDC system and record required information on study product label<br>• Provide reimbursement<br>• Schedule next study visit (6 weeks out ± 5 days)<br>• Review diary cards, including proper completion instructions. Distribute 2 daily diary forms; each form covers a 1-month period

**Clinical**		• If visit is separate from screening, reassess eligibility, including infection status, if treated, and risk of pregnancy<br>• Assess for AEs and concomitant medication<br>• Complete assessment of uterine cavity length by sound or pipelle. If cavity length measures less than 5.5 cm, subject must be discontinued and insertion should not be completed.<br>• Collect information on subject's pain after speculum placement and prior to sounding of the uterus (baseline pain assessment)<br>• Insert assigned IUD<br>• Assess ease of insertion<br>• Collect information on subject's pain after insertion (immediately and 10 minutes after insertion)<br>• Distribute a home pregnancy test with instructions to complete the test if subject has any suspicion that she may be pregnant.<br>• Distribute Home Post Placement Pain Form for subjects to record their maximum daily pain for the 6 consecutive days following IUD placement, satisfaction with IUD and use of pain medications.
**Laboratory**	Urine	• Test urine for pregnancy
	STI	• Retest for STIs if needed

Note: If enrollment does not immediately follow the screening procedure, confirmation of eligibility must be performed using clinical judgement to decide whether repeat pregnancy, STI testing, or other assessments are needed and if contact information should be reviewed. In addition, collection of information on AEs and concomitant medication changes will occur.

Note: At the discretion of the investigator, IUD insertion may be attempted a second time on the day of enrollment using the same device if no contamination issues occurred. If contamination occurred with the initial IUD on the day of enrollment, a second product may be obtained for insertion on same day of enrollment only. Procedures for obtaining second IUD will be outlined in the Health Decisions Study Manual. Reattempts using a different device or on a different date after enrollment are not allowed. Reinsertion post enrollment insertion is also not allowed.

Note: An Ultrasound does not need to be performed to confirm IUD placement.

Note: No anaesthesia may be used on first insertion attempt. Cervical anaesthesia only may be used if indicated per Investigator discretion for second insertion attempt on same day. Misoprostol is not allowed. Tenaculum site should not be anesthetized.

11.2 Clinic-Based Follow-up Visits

At 6-weeks, 3-months, 6-months, 12-months, 24-months, and 37-months post-insertion, all participants will return for follow-up visits (Table 04). During these visits, collection of information on AEs, concomitant medications, IUD acceptability, and potential concerns or side effects related to the study product will occur. To verify the presence of the IUD, the clinician will check for the IUD strings using a visual inspection, digital palpation and/or sweep for strings using a cytobrush. If the IUD strings are not visible or palpable, or if there is clinical suspicion of expulsion, clinicians will proceed with an ultrasound examination to verify that the IUD is in position (Section 9.7, Appendix 2). Participants will be scheduled for their next follow-up visit or call. Staff will also encourage participants to return to the clinic for adverse events or any other concerns at any time. If at any visit the Investigator determines that a participant should be discontinued, follow Early Discontinuation Visit procedures (Section 11.5).

11.2.1 Visit 2 (6-weeks post-insertion ± 5 days)

Study staff will collect AEs and new or changed concomitant medications, collect and review diaries with the participants, ask IUD acceptability questions, and complete the follow-up assessment forms. Clinicians will check for IUD strings as above. Assess (and treat as applicable) for STIs/RTIs, as well as check for pregnancy. Participants will be reimbursed and be scheduled for their 3-month follow-up visit. Reconfirm participant's contact information.

11.2.2 Visit 3 (3 months post-insertion ± 14 days)

Study staff will collect AEs and new or changed concomitant medications, collect and review diaries with the subjects, ask IUD acceptability questions, and complete the follow-up assessment forms. Clinicians will check for IUD strings as above. Assess (and treat as applicable) for STIs/RTIs, as well as check for pregnancy. Participants will be reimbursed and be scheduled for their 6-month follow-up visit. Reconfirm participant's contact information.

11.2.3 Visit 4 (6 months post-insertion ± 14 days)

Study staff will collect AEs and new or changed concomitant medications, collect and review diaries with the subjects, ask IUD acceptability questions, and will complete the follow-up assessment form. Clinicians will check for IUD strings as above. Assess (and treat as applicable) for STIs/RTIs, as well as check for pregnancy. Participants will be reimbursed and be scheduled for their 9-month phone call. Reconfirm subject's contact information.

11.2.4 Visit 5 (12 months post-insertion ± 14 days)

Study staff will collect AEs and new or changed concomitant medications, collect and review diaries with the subjects, ask IUD acceptability questions, and will complete the follow-up assessment form. Clinicians will check for IUD strings as above. Assess (and treat as applicable) for STIs/RTIs, as well as check for pregnancy. Participants will be reimbursed and be scheduled for their 18-month phone call. Reconfirm subject's contact information.

11.2.5 Visit 6 (24 months post-insertion ± 14 days)

Study staff will collect AEs and new or changed concomitant medications, collect and review other contraceptive use forms with the subjects, ask IUD acceptability questions, and will complete the follow-up assessment form. Clinicians will check for IUD strings as above. Assess

(and treat as applicable) for STIs/RTIs, as well as check for pregnancy. Participants will be reimbursed and be scheduled for their 30-month phone call. Reconfirm participant's contact information.

11.2.6 Unscheduled visits (any time the participant returns for services outside the schedule)

Unscheduled visits can occur any time that an Investigator feels additional assessment is warranted. During unscheduled visits, the Investigator will perform any study assessments as necessary to provide appropriate treatment. Participants should be encouraged to contact the site for any adverse events that occur to determine if an unscheduled visit is warranted. For serious events, participants should always obtain appropriate immediate medical care and the site should request release of medical records by subject upon notification of the event.

Based on the results of any unscheduled visit assessments, if the Investigator determines that the subject should be discontinued, completion of the Early Discontinuation Visit procedures (as detailed in Section 11.6) will occur and the subject status will change to discontinued from the trial.

Table 04: Clinic-Based Follow-up Visit Procedures (Visits 2, 3, 4, 5, and 6; Unscheduled Assessments as indicated)

Component	Procedures
**Administrative and Regulatory**	• Review/update contact information<br>• Provide reimbursement<br>• Schedule next study visit/call<br>• File any paperwork<br>• For Visits 2, 3, 4 and 5, collect and review daily diaries with subject (Visit 2 collect 2 forms, Visit 3 collect 2 forms, Visit 4 collect 4 forms and Visit 5 collect 7 forms). Review proper completion instructions, if necessary.<br>• For Visit 2, 3, and 4, distribute daily diary forms; each form covers a 1-month period (Visit 2 distribute 2 forms, Visit 3 distribute 4 forms and Visit 4 distribute 7 forms)<br>• For Visit 5 and 6, distribute 2 Other Contraceptive Use forms; each form covers a 6-month period<br>• For Visit 6, collect and review Other Contraceptive Use forms (2 forms should be collected)

**Clinical**		• Ask about IUD expulsion or removal<br>• Confirm the IUD is still in place (Section 9.7, Appendix 2)<br>• Complete follow-up form<br>• Assess for AEs and concomitant medications<br>• Collect information related to previous full menstrual cycle, including subject's pain and bleeding<br>• Ask IUD acceptability questions<br>• Evaluate for STIs/pelvic infection based on clinical judgement and treat per standard of care<br>• Remind subject to complete the diary<br>• Distribute additional home pregnancy tests as needed with instructions to complete the test if participant has any suspicion that they may be pregnant prior to their next visit.
**Laboratory**	Urine	• Test urine for pregnancy (subject will become a screen failure if initial urine pregnancy test comes back positive)
**Note:** If clinically indicated and per standard of care, conduct a pelvic exam, ultrasound, and/or test for chlamydia/gonorrhea. If the subject returns for an unscheduled visit, but not all assessments are required, perform assessments based on need and purpose of visit.		

11.3 Telephone Call 1, 2, and 3 (9 months, 18 months, and 30 months post-insertion ± 14 days)

The site staff will call the participant at 9-months, 18-months and 30-months post-insertion to administer the follow-up form, collect AEs, and to schedule the next clinic visit (or to schedule an earlier visit per clinician discretion).

Table 05: Phone Call Procedure at 9-months, 18-months, and 30-months Post-Insertion

Component	Procedures
**Administrative and Regulatory**	• Review/update contact information<br>• Schedule next study visit<br>• File any paperwork

**Clinical**	• Ask the subject if she has noticed any evidence of the IUD falling out or if she thinks she may be pregnant<br>• Ask about IUD removal<br>• Collect information related to previous full menstrual cycle, including subject's pain and bleeding<br>• Remind subject to complete the diary form at Call 1 and other contraceptive use form at Calls 2 & 3<br>• At Calls 2 & 3, collect information from 1 completed other contraceptive use form<br>• Ask IUD acceptability questions<br>• Assess for AEs<br>• Complete Telephone Call Form

11.4 Exit (37 months post-insertion ± 14 days) or Early Discontinuation Visit

At 37 months or at any time a participant discontinues the study, she will complete Exit Visit procedures as defined in Table 06.

During the Exit visit, collection of information on AEs, concomitant medications, IUD acceptability, and potential concerns will occur. Collection and review of diary or other contraceptive use forms (whichever form type was dispensed at previous visit) will occur with the subjects and IUD acceptability questions will be asked. Study clinicians will verify the presence of the IUD strings; if strings are not present, follow the procedures outlined in Appendix 2.

After all IUD assessments are complete, the clinician will disclose which IUD the subject was using. Those assigned to ParaGard may continue to use that product under the care of primary providers. Those assigned to the test IUD will have the product removed.

All study participants wishing to continue using a different form of contraception after the study will be referred for ongoing contraceptive care within the routine health services, including those continuing use of ParaGard. Options for continued protection from unintended pregnancies should be discussed. A short acting bridge contraceptive (condoms, 1 month of birth control pills, or 1 depo provera shot) should be provided if requested by the subject for use until referral to prevent any pregnancies occurring prior to Telephone Call 4.

Site staff will provide women with a home pregnancy test and instructions to perform the test 17 days (+ 3 days) post-removal and will be scheduled for the pregnancy test confirmation call the same day as testing. During the phone call, follow up regarding ongoing AEs, if applicable, will also be performed. If there are study related AE or new SAEs identified during the phone call, they should be followed until resolution or stabilization.

Table 06: Clinic-Based Exit Visit and Early Discontinuation Visit Procedures

Component	Procedures
**Administrative and Regulatory**	• Review/update contact information (Note: Study staff should call the participant's main contact phone number prior to visit end to ensure number correctly connects to the subject.)<br>• Provide reimbursement<br>• Schedule telephone follow-up call to be made 17 days (+ 3 days) after Exit Visit to document the pregnancy test results (completed at home after removal)<br>• Complete final subject status form<br>• File any paperwork
**Clinical**	• Ask about IUD expulsion or removal<br>• Confirm the IUD is still in place as per steps in Appendix 2<br>• Complete follow-up assessment form<br>• Assess for AEs and concomitant medications since previous visit<br>• Collect and review daily diary or other contraceptive use forms as applicable with subject (Collect all forms dispensed at previous visit)<br>• Collect information related to previous full menstrual cycle, including subject's pain and bleeding<br>• Ask IUD acceptability questions, including information on subject's desire to continue IUD use, prior to unblinding the subject<br>• Evaluate for STIs/pelvic infection as needed based on clinical judgement and treat per standard of care<br>• Disclose the IUD the woman is using<br>• If the subject is using ParaGard, ask if she wishes to continue. If not, remove the IUD. If the subject chooses to continue using ParaGard, she should be referred to her primary provider for continued IUD follow-up.<br>• If the subject is using the Mona Lisa product, remove IUD<br>• Document IUD continuation/removal information.

		• Provide contraceptive counselling and care referral if applicable:<br>○ Options for continued protection from unintended pregnancy<br>○ Provide short acting bridge contraceptives as desired (e.g., until referral)<br>• Distribute a home pregnancy test with instructions to complete the test 17 days (+ 3 days) after removal
**Laboratory**	Urine	• Test urine for pregnancy (confirmatory quantitative blood pregnancy test should be done if initial urine pregnancy test comes back positive) prior to disclosing IUD

Note: All labs and clinical IUD assessments must be done before IUD disclosure.

11.5 Telephone Call 4 for Pregnancy Test Result (17-days post-removal + 3 days)

The site staff will call the participant 17-days post-Exit visit to obtain the results of the home pregnancy test that was to be done that day and to obtain AE information.

Table 08: Phone Call Procedure at 17-days Post-Removal

Component	Procedures
**Administrative and Regulatory**	• File any paperwork
**Clinical**	• Record the result of the pregnancy test<br>○ If positive, the subject should return to the site for evaluation, including confirmatory pregnancy testing and ultrasound<br>• Assess for resolution of ongoing related AEs and all serious adverse experiences (SAEs), if applicable<br>• Collect information on new related AEs and all SAEs and follow until resolution

11.6 Early Discontinuation Visit

At any time a subject decides to discontinue her participation in the study (i.e., to remove the IUD) or is discontinued from the study for any other reason (e.g., product expulsion, pregnancy), she will be advised to return to the research site or clinic to have the Exit visit procedures completed as an early discontinuation visit. If the subject is willing, she also should be encouraged to complete the telephone call #4 (17 days after the exit visit) to provide the results of the home pregnancy test that was to be done that day (provided subject is not already known to be pregnant) and provide any AE information.

12 SAFETY

12.1 Adverse Events

An adverse event (AE) is any unfavorable and unintended sign (including an abnormal laboratory finding), symptom or disease temporally associated with the administration of an investigational product, whether or not related to that investigational product. An unexpected AE is one type that is not *a priori* identified in nature, severity, or frequency in the current Investigator's Brochure or of greater severity or frequency than expected based on the information in the Investigator's Brochure.

The Investigator (or designee) will review, via direct discussion with the subject, her history since her previous visit in order to ascertain the occurrence of any AEs since the previous visit and record the information in the site's source documents. The history should include asking how she felt since her last visit or call as well as directly and indirectly asking if she has experienced any AEs. A review of the subject's diary with her should be completed at Visits 2, 3, 4 and 5 to correct errors and inconsistencies and to identify any possible AEs reported in the diary.

The reporting period for AEs is the period immediately following the subject signing the informed consent through Call 4/Follow-up. All AEs must be recorded in the subject's electronic CRFs and must include the following information (when applicable):

- Specific condition or event.
- Indication of whether the condition was preexisting and if so, whether it has worsened in severity or frequency in which case it is reported as an AE. Conditions present prior to study entry (screening) that do not worsen in severity are considered medical history, not AEs. Conditions identified and treated at screening, i.e. laboratory abnormalities, UTIs, gynecologic infections including Chlamydia, gonorrhea, candida, bacterial vaginosis (BV) are included in medical history and are not reported as AEs.
- Date of occurrence.
- Date of resolution. If the event has not resolved at the end of the study-reporting period, it will be documented as still present on the case report form.
- Severity: AEs that change in intensity are recorded at the intensity level that is the most severe reported by the subject over consecutive days. If the intensity category changes over a non-consecutive period of time, then these changes should be recorded separately (with distinct onset dates).
- Relationship to study medication as evaluated by the Investigator (causality assessment).
- AE outcome (recovered, ongoing, etc.)
- Seriousness according to the approved regulatory classification outlined in Section 12.2.

Severity

The Investigator is responsible for assessing the severity of each AE using the following definitions for severity criteria:

- Mild – events require minimal or no treatment and do not interfere with the subject's daily activities.
- Moderate – events result in a low level of inconvenience or concern with the therapeutic measures. Moderate events may cause some interference with functioning.
- Severe – events interrupt a subject's usual daily activity and may require systemic drug therapy or other treatment. Severe events are usually incapacitating.

Relationship to Investigational Product

The Investigator is responsible for assessing the relationship between the AE and the investigational product. The Investigator must determine whether there is a reasonable possibility that the investigational product caused or contributed to an AE. The relationship assessment, based on clinical judgment, often relies on the following:

- A temporal relationship between the event and administration of investigational product;
- A plausible biological mechanism for the investigational product to cause the AE;
- Another possible etiology of the AE; or
- Previous report of similar AEs associated with the investigational product or other agents in the same class.

The terms used to assess the relationship of an event to the investigational product are:

**Causality Assessment**	**Criteria for Assessment (note that re-challenge does not apply in this study)**
Definitely Related	The experience occurs immediately following investigational product administration, related pharmacologically (not related to underlying condition/concurrent disease or other drugs or chemicals)
Probably Related	The experience follows a reasonable temporal sequence from the time of drug administration and follows a known response pattern to the suspected investigational product and cannot be reasonably explained by other factors such as the participant's clinical state, therapeutic intervention or concomitant therapy.
Possibly Related	The experience follows a reasonable temporal sequence from the time of drug administration and/or follows a known response pattern to the investigational product but could have been produced by other factors such as the participant's clinical state, therapeutic intervention or concomitant therapy.
Unlikely Related	Improbable temporal relationship. The experience was most probably produced by other factors such as the participant's clinical state, therapeutic intervention or concomitant therapy and

	does not follow a known response pattern to the investigational product.
Not Related	There is not a reasonable possibility that the AE is related to the investigational product; when an AE is assessed as not related to the investigational product, an alternative etiology, diagnosis or explanation for the AE should be provided. If new information becomes available, the relationship of any AE should be reviewed again and updated as required.
Insufficient data to assess	Selection of this rating should usually not occur in a clinical study because the Investigators have an obligation to obtain and provide this information. In exceptional instances, this rating may be used in order to avoid delay in initial reporting of fatal or life-threatening SAEs from clinical studies. Such cases should include documentation in the comments section of the CRFs/database with rationale for why an assessment could not be made.

Participants will be provided instructions for contacting the study site to report any untoward medical occurrences they may experience, except for possible life-threatening events, for which they will be instructed to seek immediate emergency care. Participants will be able to seek evaluation at the study site, where feasible and medically appropriate. With permission of the participant, and whenever possible, records from all non-study medical providers related to untoward medical occurrences will be obtained for review. All SAEs and AEs resulting in method discontinuation will be recorded on study CRFs. Trained MedDRA coders will code AE data centrally. Participants who have on-going SAEs or related AEs at their last study visit will be followed until resolution or stabilization, or referred for additional care.

12.2 Serious Adverse Experiences (SAE)

An event is considered an SAE if in the view of either the Investigator or sponsor, it meets the criteria as outlined in 21 CFR 312.32 (a) as per the following:

> "any adverse drug experience occurring at any dose that results in any of the following outcomes: death, a life threatening adverse drug experience, inpatient hospitalization or prolongation of existing hospitalization, a persistent or significant disability/incapacity or a congenital anomaly/birth defect. Important medical events that may not result in death, be life-threatening or require hospitalization may be considered a serious adverse drug experience when, based upon appropriate medical judgment, they may jeopardize the patient or subject and may require medical or surgical intervention to prevent one of the outcomes listed in this definition. Examples of such medical events include allergic bronchospasm requiring intensive treatment in an emergency room or at home, blood dyscrasias or convulsions that do not result in inpatient hospitalization or development of drug dependency or drug abuse."

In addition, cancer and drug overdose are included in this classification of an SAE.

12.2.1 Serious Adverse Experience Reporting

Study sites will document all SAEs that occur (whether or not related to study product). The collection period for new SAEs will begin after informed consent is obtained and end at the time Follow-Up/Call 4 occurs. SAEs must be followed until resolution, even if this extends beyond the study-reporting period. Resolution of a SAE is defined as the return to baseline status or stabilization of the condition with the expectation that it will remain chronic.

All SAEs will be reported promptly in accordance with the FDA and NICHD regulations and recorded on the appropriate forms. As the holder of this IND, FHI 360 is responsible for complying with the reporting requirements of SAEs to the FDA in accordance with 21 CFR 812.40. Health Decisions will serve as the third-party monitoring body for SAEs.

When any SAE, regardless of causality or relationship to the study product, is encountered during this clinical trial at an Investigator's site, the Investigator, in accordance with 21 CFR 812.150(a1), will notify NICHD, FHI 360, and Health Decisions within 24 hours of the identification/awareness of the SAE by reporting the event as specified in this protocol and via the electronic data capture system. The Investigator (or designee) should complete the SAE eCRF within the EDC system which will send the appropriate notifications to the Medical Monitors and Program Directors at both sponsors as well as the appropriate Health Decisions staff. In the event of a system outage or other complication, notification may be made to the FHI 360 Medical Monitor at: 919-544-7040, ext. 11507. Immediately following phone communication, the Investigator is to follow up with the SAE Report submission by fax or email to both FHI 360 and Health Decisions: FHI 360 at 919-544-7261 or knanda@fhi360.org, and Health Decisions at 1-919-967-1145 or nichd@healthdec.com. Health Decisions will then notify the NICHD Medical Monitor by facsimile or email. Once the outage or complication has been resolved, submission of the event should promptly be done through the EDC system.

Any additional SAE supporting documentation should be provided whenever possible (with subject identification information redacted) to verify the medical diagnosis. This includes hospital discharge summaries, lab report, death certificates/autopsy reports (where applicable), surgical procedure summaries, histology reports, and imaging reports. These supporting documents should be uploaded to the study document repository page or EDC system within 3 days of receipt at the site.

NOTE: Investigators should not wait to collect the additional information needed to fully document the event before submission of an SAE. An SAE report should be completed for any SAE that is experienced after the subject has signed the ICF, even if investigational product has not yet been administered.

FHI 360 will be responsible for the appropriate review and compliance with regulatory reporting requirements to the FDA of the SAE, while Health Decisions will work with sites to ensure the SAE is properly recorded and will notify the Medical Monitors accordingly. If warranted, the study Medical Monitors will recommend if it is required to convene the DSMB or if the trial should be stopped prematurely, undergo modification, or be placed on hold. Health Decisions may also recommend to the Medical Monitors if any of these actions are warranted based on the SAE reports.

In accordance with the standard operating procedures and policies of the IRB, the Principal Investigator (or designee) will report SAEs to the IRB. This responsibility may be handled by Health Decisions in the case of central IRB reporting, if allowed.

12.3 Medical Monitor

Both FHI 360 and NICHD will maintain a Medical Monitor and backup Medical Monitor(s), as needed.

NICHD:
Jill Long, MD, MPH, MHS (Contraception Research Branch, 6710B Rockledge Dr., Room 2335, Bethesda, MD 20817, Telephone: 301-496-1662; email: jill.long@nih.gov), is the NICHD Medical Monitor for the protocol. Dr. Long has experience as a Medical Monitor on NIH clinical protocols. She is board certified in Obstetrics and Gynecology.

FHI 360:
Kavita Nanda, MD, MHS at FHI 360 (359 Blackwell Street, Suite 200, Durham, NC 27701, Office Telephone: 919 544 7040, ext. 11507, Fax 919-544-7261; email: knanda@fhi360.org) is the FHI 360 Medical Monitor for the protocol. Dr. Nanda is board certified in Obstetrics and Gynecology and has served as a Medical Monitor on FHI 360 protocols for more than 15 years.

Each Medical Monitor will be responsible for evaluating requests for waivers and for safety monitoring and evaluation at their respective sites. A detailed medical monitoring plan will be written before the study begins. Together the Medical Monitors will review protocol safety information and provide a final recommendation based on those from the DSMB, FHI 360, or Health Decisions.

13 PREGNANCY DETERMINATION AND FOLLOW UP

The incidence of pregnancy will be assessed. We will be testing study participants at study visits using urine pregnancy tests and a home pregnancy test 17 days (+ 3 days) after IUD removal. **Sites will record information regarding pregnancy on all subjects becoming pregnant during the treatment phase of the study through 17 days after last study product use.**

If a subject becomes pregnant during the trial, the Principal Investigator (or designee) must inform Health Decisions of the pregnancy within 24 hours of determination. The subject must be discontinued from the trial, and all procedures scheduled for the Early Discontinuation Visit as detailed in Section 11.6 should be performed. The site will submit a Pregnancy Notification form and End of Study eCRF to Health Decisions within 24 hours of determination. Health Decisions will notify NICHD and FHI 360. Additional pregnancy data will be submitted as it becomes available.

The Investigator will estimate the date of conception based upon results of the following criteria. If there is uncertainty about the dating it will be reviewed with the Medical Monitors.

- Transvaginal ultrasound examination
- Quantitative β-hCG determination
- Last menstrual period (per daily diary information through first year and patient report after first year)
- Pelvic and/or abdominal examination

- Pregnancy outcome
- Investigator estimation in the absence of above criteria

Sites will counsel subjects who become pregnant during the study regarding pregnancy options and refer them for appropriate care. All pregnancies will be monitored until outcome (i.e. 9 months follow-up or until outcome information is obtained through medical records). Information from medical records will also be collected on pregnancy outcome data including maternal or fetal complications, spontaneous abortions, ectopic pregnancies, stillbirths, and live preterm and full-term births.

Pregnancy itself is not a SAE; however, pregnancies associated with outcomes outlined in Section 12.2 will be considered serious adverse events (SAEs). Pregnancy outcome data will be collected for analysis. Reports of pregnancy outcome and examinations during the pregnancy will be requested from the attending physician/outside clinic, if applicable. Pregnancy outcome data will include the rates of spontaneous abortion, stillbirth and live preterm and full-term births. In addition, congenital malformations and anomalies will be recorded and summarized. If a subject continues with her pregnancy through childbirth, every effort will be made to maintain contact with her so that health of the baby can be evaluated at six and twelve months and appropriate medical records obtained if necessary.

14 MONITORING

14.1 Clinical Site monitoring

The Principal Investigator and sub-Investigators will allow representatives from Health Decisions, FHI 360 and NICHD direct access to all eCRFs, source documents, and corresponding portions of the medical records for each participant at mutually convenient times for periodic review before, during, and after the study has been completed. The monitoring visits provide the monitor with the opportunity to:

- Initiate the research site.
- Evaluate the progress of the study.
- Verify the accuracy and completeness of the eCRFs.
- Ensure that all protocol requirements, applicable FDA regulations, and Investigators' obligations are being fulfilled.
- Resolve any inconsistencies in the study records.
- Close out the trial at the research site.

Before the study begins a detailed clinical monitoring plan will be developed. Briefly, the study clinical monitors will:

- Review ICFs, procedures, and documentation
- Assess compliance with the study protocol, Good Clinical Practices (GCP) guidelines, and applicable regulatory requirements
- Verify all SAEs in source documents have been reported on CRFs and in accordance with regulatory guidelines

- Perform source document verification to ensure the accuracy and completeness of study data
- Verify proper biological specimens collection
- Verify proper storage, dispensing, and accountability of study products
- Assess implementation and documentation of internal site quality management procedures
- Verify that current license/certification is available on site for study staff listed on the current Statement of Investigator Form, and Delegation of Responsibilities Log/Form.

The Principal Investigator or designee will allow study monitors to inspect study facilities and documentation (e.g., ICFs, clinic and laboratory records, other source documents, CRFs), as well as observe the performance of study procedures. Investigators will also allow inspection of all study-related documentation by authorised representatives of OHRP and local and US regulatory authorities. Sites will maintain a site visit log to document all visits.

14.1.1 Quality control and quality assurance

In addition to routine monitoring, the Sponsors or its designee may, at its discretion, perform site audits/quality control visits. The purpose of such visits will be to evaluate site trial conduct and compliance with the protocol, SOPs, GCP, the applicable regulatory requirements and discuss operational issues or problems. If an audit is performed, a site must provide the auditors with direct access to all relevant records and documentation related to the study. All participant records and other study documentation must be filed and accessible on short notice (three to five days) during the study and subsequent retention period. Documents should be adequate to reconstruct the course of study events. Refer to Section 20 for requirements for data retention.

The Investigators and sites may also be subject to a field audit by the regulatory agencies. Such an audit could occur while the study is in progress, after the study is completed, or when the data are under review by a regulatory agency. All participant records and study documentation should be filed and made accessible as noted above for any regulatory agency review. The site should notify NICHD, FHI 360 and Health Decisions as soon as they become aware of an audit scheduled by any regulatory agency.

14.1.2 Source documents and access to source data/documents

Sites will maintain appropriate source documents, as detailed in the study monitoring plan. All investigative site records (source documents and other subject records) will be kept in a secure and hazard free storage area. Access will be restricted to study personnel authorized to handle research documents. These records are to be retained for at least 2 years after approval of the marketing application or at least 2 years have elapsed since formal discontinuation of clinical development for this investigational product. NICHD and FHI 360 should be notified before destruction of any site records.

14.2 Regulatory

Investigator will maintain and store in a secure manner complete, accurate, and current study records throughout the study. The Investigator will retain all study records as authorized by the operations team in compliance with local regulations. Study records include administrative documentation including protocol registration documents and all reports and correspondence relating to the study as well as documentation related to each participant screened for and/or enrolled in the study including ICFs, locator forms, case report forms, notations of all contacts with the participant, and all other source documents.

The trial will be conducted in compliance with the protocol, GCP, and the applicable regulatory requirement(s) of the US and study site countries. Health Decisions is responsible for assuring that the essential documents maintained in the trial master file at each research site for each Sponsor are accurate and complete. In addition, Health Decisions will be responsible for maintaining central documentation to demonstrate GCP compliance. Files will be maintained according to written SOPs.

15 DISCONTINUATION AND REPLACEMENT OF SUBJECTS

A subject will be considered to have completed the clinical trial after she has completed 37 months of using the study product and after the final procedures have been performed.

15.1 Early Discontinuation of Study Product

Subjects are free to request IUD removal at any time and for any reason specified or unspecified and without prejudice.

Additionally, the IUD may be removed at any time if the subject, the Investigator, or the Sponsor feels that it is not in the subject’s best interest to continue. The following is a list of reasons that IUD removal may be indicated:

- Subject withdrawal of consent (or assent) (e.g. due to personal reasons)
- Subject is not compliant with study procedures in the opinion of the Sponsor or Investigator
- Adverse event that in the opinion of the Investigator would be in the best interest of the subject to have the IUD removed
- Inter-current illness or other safety concern that, in the judgment of the Investigator, should result in IUD removal.
- Protocol deviation requiring IUD removal
- Early termination of study
- Use of prohibited medications, including any outlined in the Inclusion/Exclusion criteria (see Sections 7.2, 7.3, and 8.2)

Subjects **must be** discontinued for the following reason:

- Pregnancy
- IUD expulsion
- IUD removal
- Failed IUD insertion (after 2 attempts on day of Enrollment)

- Uterine perforation

If an Investigator determines that a subject's IUD should be removed due to an AE, the Investigator or other care provider will provide appropriate treatment until the AE has resolved or stabilized after IUD removal.

The Investigator will document the reason for IUD removal in the subject's source documents and in the final status form. If withdrawal from the study is due to an adverse event, this information will also be recorded on the AE form.

Subjects who discontinue study treatment should come in for an early discontinuation visit as soon as possible as outlined in Section 11.6 regardless of the reason for discontinuation with the exception of those subjects who have lost contact with the research site.

15.1.1 Lost to Follow-Up

A subject cannot be considered withdrawn due to becoming lost to follow-up (LTFU) until the research site performs and documents at least three (3) attempts to contact her. Documentation must include at least one letter sent via a method that allows a "return receipt" to be requested with instructions provided to the subject to call the research site. Every effort must be made to follow-up with subjects who terminate with product-related adverse experiences, in order to determine the final outcome.

15.2 Withdrawal of Subjects from the Study

A subject may be withdrawn from the study at any time if the subject, the Investigator, or the Sponsor feels that it is not in the subject's best interest to continue, including non-compliance or for safety concerns.

All subjects are free to withdraw from participation at any time, for any reason, specified or unspecified, and without prejudice.

Reasonable attempts will be made by the Investigator to provide a reason for subject withdrawals. The reason for the subject's withdrawal from the study will be specified in the subject's source documents and the final status form.

All efforts should be made to contact the woman when she decides to discontinue so that appropriate discontinuation safety procedures can be performed and data can be obtained. Such contact will never be used to dissuade any subject who wishes to terminate the study.

Women who unexpectedly become pregnant during the study will be removed from further participation. However, their pregnancy outcome results will be ascertained as outlined in Section 13 and documented on the Pregnancy Outcome CRF.

15.3 Replacement of Subjects

Subjects who withdraw from the study treatment or withdraw from the study will not be replaced, regardless of the reason for discontinuation.

15.4 Study Product Errors

Study product will be stored according to standard procedure at each of the participating study sites. When a subject is randomized, the IUD will be obtained for the subject based on the

product identified in the EDC system (refer to Section 9.1). Product errors are unlikely as stock product packaging will be different between the two IUDs; however, if one occurs, the site should inform Health Decisions immediately upon determination.

16 PROTOCOL DEVIATIONS

A protocol deviation occurs when the subject, Investigator, or Sponsor fails to adhere to significant protocol requirements affecting the inclusion, exclusion, subject safety and primary endpoint criteria. Protocol deviations for this study include, but are not limited to, the following:

- Failure to meet inclusion/exclusion criteria
- Enrolling a participant into the study without securing proper consent
- Deviating from the randomization procedures
- Informing the participant of which IUD she received prior to the exit visit

Failure to comply with Good Clinical Practice (GCP) guidelines will also result in a protocol deviation. The Sponsor will determine if a protocol deviation will result in withdrawal of a subject.

The Investigator should not deviate from the protocol without prior approval issued as a protocol waiver approved by the Medical Monitor, with the exception of minor visit window deviations as outlined for each visit/call in Section 11. In addition, all protocol deviations will be submitted within the EDC CRFs for the study.

Certain protocol deviations may require the subject to be terminated early from the study. The Medical Monitor will review if subject termination will be required based on protocol deviation information at the time that the waiver is reviewed. It is the responsibility of the research sites to report all protocol waivers and protocol deviations to their IRB according to IRB policy.

17 AMENDMENTS TO THE PROTOCOL

Any change of the clinical trial must be written and filed as an amendment to this protocol. Such amendments will be made jointly by the Sponsors and the Investigators with final approval by the Sponsors. The Principal Investigators (or designee) must submit the protocol amendment for review by the IRB and shall obtain the approval of the IRB before it is implemented.

In cases of emergency, when the protocol change or deviation is necessary in order to eliminate or reduce an immediate hazard or risk to human subjects, the amendment may be implemented before review or approval by their IRB. In such cases, the Principal Investigator (or designee) shall notify their IRB of the change or deviation in writing within 10 working days after implementation. Any protocol-related issues that pose an immediate or significant hazard to subjects must be reported to the Sponsors immediately. If the protocol amendment is an administrative change, it will be sent to their IRB for information (updating of file).

All modifications of the clinical trial will be written and filed with FDA as an amendment to this protocol, maintaining original section identification. Such modifications will be made jointly by the Sponsors, Health Decisions, and the Investigators with the approval of all the IRBs.

18 DATA SAFETY AND EFFICACY MONITORING

18.1 Data and Safety Monitoring Board (DSMB)

An independent, autonomous Data and Safety Monitoring Board (DSMB) has been established by the NIH (NICHD). The DSMB will conduct periodic reviews of study progress indicators, subject safety and pregnancy. It is expected that three (3) such reviews occur. The Project Directors at NICHD and FHI 360 may also request additional DSMB reviews, (e.g. should any other findings/issues pertaining to safety or efficacy emerge requiring DSMB review outside of the planned periodic meeting dates).

The DSMB charter will be prepared prior to the first DSMB meeting then modified as needed before the second meeting or subsequent meetings. The Charter will address the timing of each DSMB meeting, stopping rules relative to items including SAEs, pregnancy rates, expulsion rate, etc., as well as detailed blinding and unblinding procedures. Additional information about the DSMB composition and review procedures will be contained in the charter. As the primary objective of the study is non-comparative, no adjustment to confidence interval coverage error rates will be made to account for interim analyses when reporting for pregnancy rate data. Similarly, no multiplicity adjustment will be made for secondary objectives.

The DSMB will maintain the confidentiality of its closed internal discussions and activities. Recommendations from the DSMB will be forwarded by the Project Directors, to the Central IRB, and the Principal Investigators in this Protocol for inclusion into the study TMF along with any DSMB open session discussion meeting minutes.

The primary responsibilities of the DMSB are to 1) periodically review and evaluate the accumulated study data for participant safety, study conduct and progress, and 2) make recommendations to the study Investigators and the sponsor concerning the continuation, modification, or termination of the trial. At each review, the DSMB may recommend that the study proceed as designed, proceed with design modifications, or be discontinued.

Summary results of interim data which are pooled across treatment groups may be provided to study sites for their information and for submission to the IRBs.

19 STATISTICAL METHODS AND CONSIDERATIONS

19.1 Sample Size

Below is the original estimate for the 95% upper bound of the Pearl Index for the Mona Lisa Cu IUD arm if we had randomized 800 subjects to that treatment. Since this protocol has been amended to randomize approximately 880 Mona Lisa Cu IUD subjects, the estimated 95% upper bound of the Pearl Index would be slightly lower due to the larger sample size. In addition, the power to detect differences in 12-month IUD continuations rates will be slightly higher than the original power presented below.

Randomizing 800 women to the Mona Lisa Cu IUD will provide approximately 17,700 cycles for the primary efficacy analysis among women under 36 years old at time of enrollment,

assuming 13 cycles of use per year with 84.3% evaluable cycles, an exponential discontinuation rate of 0.2 (i.e., approximately 20% per year), and 37 months of prescribed follow-up per woman. Based on this enrollment total and follow-up rate, the upper bound of the Pearl Index (pregnancies per 100 women-years of follow-up) would be approximately 1.0 if 6 pregnancies were observed (see table).

Table 09: Expected Pearl Index (pregnancies per 100 women-years of follow-up) among women under 36 years old at time of enrollment and 95% CI for Pearl Index as a function of observed number of pregnancies, assuming 800 women are randomized to Mona Lisa Cu IUD and 51.2% method continuation at month 37

Observed pregnancies	Pearl Index	95% CI
0	0.0	0.0-0.3
1	0.1	0.0-0.4
2	0.1	0.0-0.5
3	0.2	0.0-0.6
4	0.3	0.1-0.8
5	0.4	0.1-0.9
6	0.4	0.2-1.0

In terms of estimating and comparing standard 12-month IUD continuation rates, the study will achieve approximately 87% power to detect a 10% greater 12-month continuation rate for the Mona Lisa Cu IUD using a two-sided $\alpha=0.05$ level log-rank test, assuming the 12-month probability of continuation for ParaGard is 70% and allowing for up to 5% of women lost to follow-up.

19.2 Statistical analyses

A detailed statistical analysis plan (SAP) will be developed. The following is a summary of the planned analyses. Any deviations made from this summary plan will be documented in the detailed SAP.

19.2.1 Primary analysis

The primary analysis will estimate the pregnancy rate in women 35 and younger at time of enrollment based on the Pearl Index (pregnancies per 100 woman-years of follow-up) and its exact 95% confidence interval derived using a Poisson distribution assumption, after excluding cycles (28-day equivalents) in which women report using other contraceptive methods. Analysis will be done for each year of use and cumulatively through year three. Life-table methods will also be used to summarize the cumulative probability of pregnancy through the study follow-up period.

19.2.2 Analysis of secondary endpoints

The standard 12-month continuation probability and its corresponding asymptotic 95% confidence interval for each study group will be estimated using the Kaplan-Meier method. In addition, a log-rank test will be used to compare early discontinuation rates between groups at the two-sided 0.05 significance level. Similar summaries and comparisons will also be made to assesses the probabilities of discontinuation by reason (e.g., IUD expulsion, failed IUD insertion, uterine perforation, pregnancy, personal/non-medical reasons, and other medically-related reasons), as well as self-reported desire to continue IUD use at the study exit but prior to unblinding the participant.

We will analyze bleeding diary data using the methods described by Mishell, et al[17]. Statistics will include the mean, median, and range of the total number of heavy, moderate, light, or spotting days per 28-day cycle equivalent as defined on the daily diary (refer to Appendix 3). We will also report the per-subject mean and median number of cycles and the proportion of cycles that include any heavy bleeding, a prolonged bleeding episode (e.g., greater than 14 days), amenorrhea, or bleeding in more than 1/3 of days in the cycle. Details of the bleeding diary analysis will be included in the SAP, including procedures and definitions used to define individual cycles and to account for missing data.

In addition to diary data, summaries of bleeding and pelvic pain experienced between study visits (and reported on CRFs) will be described in contingency tables and displayed graphically in bar charts, by study visit and overall. Chi-squared tests for ordinal repose data (e.g., less than normal, normal, or more than normal pelvic pain) will be used to compare treatment groups at each visit. Evidence of informative censoring that is differential between groups will be explored using competing risk analysis; in the event such effects exist then analysis methods which account for informative censoring will be employed when comparing secondary outcomes between groups over time.

The proportions of subjects experiencing any other side effects will be calculated with corresponding exact confidence intervals for each group, and compared the difference between groups by Fisher’s exact tests or the Cochran-Mantel-Haenszel tests. Generalized linear model approaches for repeated occurrence side effect outcomes might be used to further assess the differences in the incidence of adverse events between study groups.

Overall satisfaction with the method will be summarized and compared between groups at the final study visit using two-sided Mantel-Haenszel chi-square tests. Similarly, the same statistical analysis methods will be used to describe ease of product insertion between groups. Changes in pain with and shortly after insertion from baseline will be described by the summary statistics (mean, median, inter-quartile, etc.) of visual analog scale measurements. The Mann-Whitney test or the analysis of variance method will be used to compare the changes in pain between groups.

For all secondary objectives, comparisons between groups will be made using two-sided 0.05 level significance tests, without adjustment for multiple comparisons.

20 DATA COLLECTION, RETENTION AND MONITORING

20.1 Electronic Data Capture System

All requested information should be entered in the Electronic Data Capture (EDC) system. Prior to the start of the clinical trial, the Principal Investigator will complete an authorized signature sheet showing the signatures and handwritten initials of all individuals who are authorized to maintain study records and submit data using the EDC system. More detailed instructions regarding the EDC system will be provided by Health Decisions in training and the instructions documents.

20.2 Data Handling and Processing

The study will use Electronic Data Capture (EDC) to create, modify, and maintain data generated for the clinical trial. The research sites will record clinical trial data in an electronic web-based CRF (eCRF). Health Decisions Data Management will follow written standard operating procedures for processing EDC data. Archiving of the EDC data will be done by the EDC vendor.

20.3 Confidentiality and Reporting of Results

Subject names will not be entered into the EDC system; instead, unique subject identifiers will be assigned. Medical records will be kept at the research site and will be available to study staff and the NICHD, Health Decisions, FHI 360, independent auditors contracted by the Sponsors, IRBs, local regulatory authorities or the FDA only. All data and information generated by the participating site as part of the trial will be kept confidential by the site principal investigator and other study personnel to the extent permitted by law. This information and data will not be used by the site principal investigator or other study personnel for any purpose other than conducting the trial.

20.4 Archival of Data.

For archiving purposes, all eCRF information will be stored via an electronic medium at completion of the trial. Audit trail information will be included. eCRFs will be available for inspection by authorized representatives from the Sponsors, from Regulatory Authorities and/or IRBs. Copies of each site's eCRF information will be supplied to each site at the completion of the trial.

20.5 Availability and Retention of Investigational Records

The Principal Investigator will maintain and store in a secure manner complete, accurate, and current study records throughout the study. All records are to be retained by the Principal Investigator for a period of at least 2 years after the latter of the following two dates: The date after approval of the marketing application or at least 2 years have elapsed since formal discontinuation of clinical development for this investigational product (per FDA CFR 812.140). These documents should be retained for a longer period (i.e., but never a shorter period), however, if required by the applicable regulatory or site-specific requirements or by an agreement with the sponsor. It is the responsibility of the sponsor to inform the Investigator/institution as to when these documents no longer need to be retained. NICHD

should be notified before destruction of any study records by their sites. For FHI 360 sites, study documents need to be retained for a minimum of 5 years until authorized in writing by the FHI 360 operations team, in accordance with FHI 360 SOPs.

Study records include administrative documentation, including protocol registration documents and all reports and correspondence relating to the study as well as documentation related to each participant screened for and/or enrolled in the study, including ICFs, locator forms, medical records, laboratory reports, drug disposition records, safety reports, case report forms, notations of all contacts with the participant, information regarding subjects who discontinued and all other source documents of pertinent data.

The trial will be conducted in compliance with the protocol, GCP, and the applicable regulatory requirement(s) of the US. In addition, FHI 360 and Health Decisions will be responsible for maintaining central documentation to demonstrate GCP compliance for the respective Sponsor sites.

20.6 Subject Confidentiality

Information on individual subjects arising from this study will be considered confidential and transmitted to the Sponsor and Clinical Research Organization (CRO) in a form that will not permit identification of individuals. Subject confidentiality will be maintained by the use of coded subject ID numbers. All study records will be kept in a secure storage area with limited access. Coded subject numbers will identify all laboratory specimens and evaluation forms in order to maintain subject confidentiality. Clinical information will not be released without the written permission of the participant, except as necessary for monitoring by the FDA, FHI 360, NICHD, Health Decisions, independent auditors contracted by the Sponsors, IRBs or local regulatory authorities.

21 ADMINISTRATIVE, ETHICAL, REGULATORY CONSIDERATIONS

21.1 Institutional Review Boards and Independent Ethics Committees

The protocol and any amendments will be reviewed by the FDA and the designated Central IRB. Documented approval will be obtained from the Central IRB, which will be Chesapeake IRB for this study. Each participating site will also seek approval from the appropriate local IRBs unless oversight is delegated to the central IRB. Copies of the submissions and approvals should be retained in the site's regulatory file and the main trial master file.

Prior to implementation of this protocol, and any subsequent full version amendments, each site must have the protocol and the protocol consent forms approved, as appropriate, by their local IRB and any other applicable regulatory entity unless delegated to the central IRB. Investigators/institutions will permit trial related monitoring, audits, IRB review and regulatory inspection(s) providing direct access to source data/documents.

21.2 Protocol Registration

The study team will register this trial on ClinicalTrials.gov, a registry database of clinical studies of human participants conducted around the world, and updated at regular, specified intervals.

21.3 Risks and Benefits

21.3.1 Risks

Investigators will make efforts to minimise risks to participants. It is not expected that study procedures will expose human subjects to unreasonable risk. Pelvic examination and procedures may cause mild discomfort and/or vaginal bleeding or spotting. Women who learn they have a reproductive tract infection, in the course of the trial may experience worry, sadness or depression. Women who exhibit symptoms suggestive of clinical depression will be referred for counselling. Participation in clinical research includes the risks of loss of confidentiality and discomfort with the personal nature of questions when discussing sexual behaviours.

Participants at sites requiring partner notification in response to diagnosed STI infection could have problems in their relationships with their sexual partners. Participants also could have problems in their partner relationships associated with the use of a contraceptive method.

Site staff will make every effort to protect participant privacy while in the study. Although study sites make every effort to protect participant privacy and confidentiality, it is possible that participants' involvement in the study could become known to others, and that social harms may result.

The risks of using the ParaGard IUD are low, but do exist. IUD users are at risk of uterine perforation (including embedment) (1/1000)[15], pregnancy, PID, or expulsion of the device (5% in the first year of use); all of these complications are rare. If perforation or IUD embedment occurs, it is possible that surgery will be needed to remove the product. Approximately 1% of IUD users experience PID in the three weeks after IUD insertion, then the risks decrease considerably thereafter.[16] Approximately 50% of users experience increases in menstrual flow and number of days of bleeding. Approximately 20% experience intermenstrual spotting/bleeding, and about 35% experience painful menses.[14] In addition, IUD insertion is uncomfortable for most women, although generally symptoms resolve within ten to fifteen minutes; symptoms are usually completely resolved within one day after insertion.

The risks of using the test product has all the general risks of any IUD. However, the test product is not well-studied, thus it has uncertain levels of the above complications and perhaps new risks. Though the test product is approved and marketed in Europe, it is a novel entity from a regulatory perspective in the U.S.

Contraceptive failure is possible with any of the IUDs; ParaGard has an estimated failure rate of less than 1% in the first year of use. If pregnancy occurs, the IUD should be removed to reduce the risks of spontaneous abortion, premature delivery, pelvic infection, sepsis, and other rare events. Failure rates for the test product are largely unknown, since they are not estimated from appropriately sized and regulated clinical studies in Europe. The contraceptive-related risks anticipated to be experienced by participants in the study are no different from those that would be anticipated among contraceptive users in Europe who have access to both products.

21.3.2 Benefits

Participants benefit from the use of a highly effective form of contraception: one of two types of copper IUDs, one of which is approved for use in some countries in Europe and in Canada, and one of which is approved for use in the United States.

Others may benefit in the future from information learned from this study. Specifically, information learned in this study may lead to improved understanding about the benefits of smaller IUDs. Participants may appreciate the opportunity to contribute to the field of reproductive health and contraceptive research.

21.4 Informed Consent

Under informed consent, the subject shall understand that she is authorizing access to medical records as required for monitors, auditors, IRBs and regulatory authorities. Subjects who agree to participate in the study must sign the ICF prior to study-specific procedures. The principles of informed consent will be implemented according to the 2013 revision of the Declaration of Helsinki and current NIH, DHHS, and FDA regulations. The ICF will be HIPAA compliant.

It is the Principal Investigator's responsibility to assure that each subject is provided an explanation of the details contained in the informed consent statement and other locally required documents prior to the individual signing the ICF. This certifies voluntary participation in the trial and is completed prior to study participation. Under informed consent, the subject shall understand that she is authorizing access to medical records as required for monitors, auditors, IRBs and regulatory authorities.

The subjects will be informed of their right to privacy and the fact that personal information will be treated as strictly confidential and will not be publicly available in accordance with HIPAA regulations. They will also be informed that the NICHD, Health Decisions, FHI 360, and the FDA have the right to inspect and possibly photocopy their medical records to verify the accuracy and completeness of the clinical trial results. The subject is to receive a copy of the signed consent form.

Prior to study participation, all study candidates will:

- Be informed of the nature and purpose of the study.
- Be given an explanation of the procedures to be followed in the study.
- Be given a description of any attendant discomforts and risks reasonably to be expected from the study, as well as from the study product.
- Be given an opportunity to ask any questions concerning the study.
- Be instructed that consent to participate in the study may be withdrawn at any time; and that the participant may discontinue participation in the study without prejudice.
- Be given a copy of the ICF.
- Be given the opportunity to decide to consent or not to consent to the study without coercion.
- Be informed of alternative contraceptive methods available.
- Be informed about their right to use Emergency Contraception (EC) during the study.

- Be given information about whom to contact if there are questions about the research, participant rights, or to report research-related injury.

21.5 Conflicts of Interest

Investigators will be affiliated with their study sites. They will receive support for this clinical trial from the NICHD/NIH or FHI 360, but will not profit from results, either positive or negative, with regard to the product being evaluated. FHI 360, the IND holder, could profit from the successful development of this product.

21.6 Confidentiality

The information on individual subjects arising from this study is to be considered confidential and transmitted to the sponsor only in a form that will not permit identification of the individual. The information obtained from the subjects that can be identified with the subject will remain confidential within the research team. Regulatory and sponsoring agencies may request access to the study records and related medical records of each participating subject; the subject's identity will remain confidential to the extent permitted by the applicable laws and regulations. The results of the research will be released to public agencies including regulatory agencies, Investigators, and research organizations without reference to items identifiable to a particular subject. The results will be published such that the identity of the subjects will not be disclosed and cannot be ascertained. National and international agencies and sponsoring agencies may request access to the medical records of each participating subject, and if requested, the subject's identity will remain confidential. All records will be kept in a secure storage area with limited access.

21.7 Publications

A Publications Committee will be formed to write a publication policy for the trial. Three levels of publications will be addressed in this policy:

- Primary publications: Those publications specifically reporting the results of the primary, secondary study, and tertiary objectives stated in the study protocol
- Secondary publications: Publications reporting on the study results using data from multiple study sites but not specifically addressing the stated objectives of the study
- Tertiary publications: Publications based on data from a unique study site and drafted by the study team at that site.

Authorship roles will be assigned primarily according to the contributions of individual members as well as the likelihood that the authors will be able to complete data analysis and manuscript preparation in a timely manner. All Investigators will be encouraged to participate in discussions regarding authorship of primary and secondary publications. Abstracts and publications resulting from this study must be approved by the Publications Committee prior to submission to scientific meetings or journals.

21.7.1 Publication Policy

Data on the use of the study product and results of all clinical and laboratory studies are considered private and confidential. NICHD and FHI 360 will encourage publication of the results of the study. Any publications or presentations that result from this study will maintain participant confidentiality. The IND and proprietary information about the investigational drug are under the supervision of the NICHD and FHI 360. The Principal Investigator agrees to submit all proposed publications, papers, abstracts, manuscripts, posters or other written materials which include data relating to the Study or the use of the Product supplied under this Agreement, and all outlines of any proposed oral presentations with respect thereto, to NICHD and FHI 360 at least thirty (30) days prior to either (a) submission of such written materials for publication or (b) any proposed oral disclosure to a third party outside of the NICHD CCTN and FHI 360 study sites. NICHD and FHI 360 shall have the right to comment on such written material or outline within (30) days of receipt, and such comments shall be considered in good faith by the Principal Investigators in determining the final form of disclosure. NICHD and FHI 360 shall also have the right to eliminate any reference to confidential or proprietary information provided to Institutions and/or the Principal Investigators or the Staff pursuant to this Agreement.

21.7.2 Authorship eligibility

Eligibility for authorship will follow the International Committee of Medical Journal Editors recommendations for determining substantive contribution to a manuscript developed for publication.

22 REFERENCES

1. ParaGard® T380A INTRAUTERINE COPPER CONTRACEPTIVE US Prescribing Information, June 2013. . http://www.accessdata.fda.gov/drugsatfda_docs/label/2013/018680s066lbl.pdf. (accessed January 10 2016).
2. Sivin I, Stern J. Long-acting, more effective copper T IUDs: a summary of U.S. experience, 1970-75. *Studies in family planning* 1979; **10**(10): 263-81.
3. Sivin I, Tatum HJ. Four years of experience with the TCu 380A intrauterine contraceptive device. *Fertility and sterility* 1981; **36**(2): 159-63.
4. Sivin I, Stern J, Coutinho E, et al. Prolonged intrauterine contraception: a seven-year randomized study of the levonorgestrel 20 mcg/day (LNg 20) and the Copper T380 Ag IUDS. *Contraception* 1991; **44**(5): 473-80.
5. The TCu380A, TCu220C, multiload 250 and Nova T IUDS at 3,5 and 7 years of use--results from three randomized multicentre trials. World Health Organization. Special Programme of Research, Development and Research Training in Human Reproduction: Task Force on the Safety and Efficacy of Fertility Regulating Methods. *Contraception* 1990; **42**(2): 141-58.
6. Hubacher D. Copper intrauterine device use by nulliparous women: review of side effects. *Contraception* 2007; **75**(6 Suppl): S8-11.
7. Abraham M, Zhao Q, Peipert JF. Young Age, Nulliparity, and Continuation of Long-Acting Reversible Contraceptive Methods. *Obstetrics and gynecology* 2015; **126**(4): 823-9.
8. Benacerraf BR, Shipp TD, Lyons JG, Bromley B. Width of the normal uterine cavity in premenopausal women and effect of parity. *Obstetrics and gynecology* 2010; **116**(2 Pt 1): 305-10.
9. Koch P, Reinhardt P, Soyka E. Intrauterine contraception using the Copper Mini-Gravigard 7 IUD: summary of 328 case histories. *Contracept Deliv Syst* 1981; **2**(1): 171-6.
10. Petersen KR, Brooks L, Jacobsen N, Skoby SO. Clinical performance of intrauterine devices in nulligravidae: is the length of the endometrial cavity of significance? *Acta Eur Fertil* 1991; **22**(4): 225-8.
11. Otero-Flores JB, Guerrero-Carreno FJ, Vazquez-Estrada LA. A comparative randomized study of three different IUDs in nulliparous Mexican women. *Contraception* 2003; **67**(4): 273-6.
12. Ott MA, Sucato GS. Contraception for adolescents. *Pediatrics* 2014; **134**(4): e1257-81.
13. Committee on Adolescent Health Care Long-Acting Reversible Contraception Working Group The American College of Obstetricians Gynecologists Committee opinion no. 539: adolescents and long-acting reversible contraception: implants and intrauterine devices. *Obstetrics and gynecology* 2012; **120**(4): 983-8.
14. Curtis KM, Tepper NK, Jatlaoui TC, et al. U.S. Medical Eligibility Criteria for Contraceptive Use, 2016. *MMWR Recommendations and reports : Morbidity and mortality weekly report Recommendations and reports / Centers for Disease Control* 2016; **65**(3): 1-103.
15. Heinemann K, Reed S, Moehner S, Minh TD. Risk of uterine perforation with levonorgestrel-releasing and copper intrauterine devices in the European Active Surveillance Study on Intrauterine Devices. *Contraception* 2015; **91**(4): 274-9.
16. Farley TM, Rosenberg MJ, Rowe PJ, Chen JH, Meirik O. Intrauterine devices and pelvic inflammatory disease: an international perspective. *Lancet* 1992; **339**(8796): 785-8.
17. Mishell DR Jr, Guillebaud J, Westhoff C, Nelson AL, Kaunitz AM, Trussell J, Davis AJ. Recommendations for standardization of data collection and analysis of bleeding in combined hormone contraceptive trials. *Contraception* 2007; **75**(1): 11-1.

23 INVESTIGATOR OBLIGATIONS

Clinical research studies are subject to the regulations of the FDA. Prior to beginning the study, the Principal Investigator will be asked to demonstrate compliance with ICH E6, 8.2 and 21 CFR 312 by providing the signed essential documents required for the clinical trial. The responsibilities imposed upon Principal Investigators by the FDA are summarized in the "Statement of Investigator Form" (Form FDA-1572) which documents that he or she will follow the FDA regulations with respect to this study.

The Principal Investigator agrees to assume the responsibilities stated on the Form FDA-1572, and signifies his or her agreement by signing the Form FDA-1572. These responsibilities include:

- To conduct the study in accordance with the Study Protocol, Investigator Brochure, GCPs, ICH, and Declaration of Helsinki.
- To secure prior approval of the study by an appropriate IRB. This board should be constituted in conformity with FDA regulations.
- To report on the progress of the study to the IRB and to submit a final report within three (3) months of the conclusion of data collection.
- To maintain current records of the receipt, dispensing, and disposition of study product and to return all unused product to the sponsor or the sponsor's designated agent.
- To obtain a valid, fully informed, freely given written consent from each subject who participates in the study.
- To maintain adequate study documentation, hospital records, laboratory results and lab records, and to store these case histories for a minimum of two years following notification by the sponsor that all investigations have been discontinued or that the FDA has approved the study product application, or according to site practice only if longer than specified here.
- To identify all sub-Investigators who will also supervise study product administration.
- To report all Adverse Events to the sponsor or designated agent promptly.
- To allow inspection or copying by the FDA, sponsor, or sponsor's designated agent of case histories and records of study product distribution.

Investigator Documentation required:

Prior to beginning the study, the Principal Investigator will be asked to comply with ICH E6, 8.2 and 21 CFR 312 by providing the following essential documents, including but not limited to:

1. An original signed Investigator Agreement page of the protocol.
2. An original signed acknowledgement of receipt of the Investigator's Brochure.
3. An IRB-approved Informed Consent (as described in section 21.4) in the local language.
4. IRB approval of protocol and informed consent.
5. Form FDA 1572, fully executed, and all updates on a new fully executed Form FDA 1572.
6. Current Curriculum Vitae (CV) for the Principal Investigator and each sub-Investigator listed on Form FDA 1572.

24 APPENDIX 1: SCHEDULE OF ASSESSMENTS

**Study Procedures**	**Screening**	**Treatment Period**											**Follow Up**	**Unscheduled**
Visit Number	**SCRN**	**V1**	**V2**	**V3**	**V4**	**C1**	**V5**	**C2**	**V6**	**C3**		**EXIT[9]**	**C4**	**UNS^**
**Treatment Period***	**N/A**	**Day 1**	**6 Wks**	**3 Mos**	**6 Mos**	**9 Mos**	**12 Mos**	**18 Mos**	**24 Mos**	**30 Mos**		**37 Mos**	**17 Days**	**VAR**
Informed consent, HIPAA Authorization, Assent (if applicable)	X													X
Assign Subject Number	X													
Obtain/Update Contact Information	X	X*	X	X	X	X	X	X	X	X		X		X
Determine Eligibility (Inclusion/ Exclusion)	X	X*												
Contraceptive Counselling	X											X		X
Demographics	X													
Medical, contraceptive, gynecological, menstrual, reproductive history	X	X*												
Vital signs[1]	X	X*												X
Pelvic exam[2, 5]	X[12]	X*												X
Assess (and treat as applicable) for STIs/RTIs	X	X*	X	X	X		X		X			X		X
Pregnancy test (urine)[8]	X	X*	X	X	X		X		X			X	X	X
Endocervical, vaginal or urine specimen collection for GC/CT NAAT[5,7]	X	X*												X
Randomize subject		X												
Provide reimbursement	X	X	X	X	X		X		X			X		X
Schedule next visit	X	X	X	X	X[3]	X	X[3]	X	X[3]	X		X[6]		X
Insert IUD		X												
Assess ease of insertion		X												
Collect subject insertion pain level[21]		X												
Dispense Home Post Placement Pain Form		X												
Collect Home Post Placement Pain Form			X											
Collect IUD continuation/removal information[20]			X	X	X	X	X	X	X	X		X		X
Collect previous menstrual cycle, bleeding, pain information[18, 20]			X	X	X	X	X	X	X	X		X		X
Ask IUD Acceptability Questions[20]			X	X	X	X	X	X	X	X		X		X
Confirm IUD placement[4]			X	X	X	X	X	X	X	X		X		X
Inquire on desire for continued IUD use[20]												X		X

**Study Procedures**	**Screening**	**Treatment Period**											**Follow Up**	**Unscheduled**
Visit Number	**SCRN**	**V1**	**V2**	**V3**	**V4**	**C1**	**V5**	**C2**	**V6**	**C3**		**EXIT[9]**	**C4**	**UNS^**
**Treatment Period***	**N/A**	**Day 1**	**6 Wks**	**3 Mos**	**6 Mos**	**9 Mos**	**12 Mos**	**18 Mos**	**24 Mos**	**30 Mos**		**37 Mos**	**17 Days**	**VAR**
Unblind IUD to subject												X		
Continue/Remove IUD[10]												X		
Provide bridge contraception, as desired												X		
Complete final subject status form													X	
Diary cards dispensed (n)[14]		X	X	X	X									X
Diary cards reviewed/discussed			X	X	X		X							X
Diary cards collected (n)[15]			X	X	X		X							X
Other contraceptive use forms dispensed (n)[16]							X		X					X
Other contraceptive use forms reviewed								X	X	X		X		X
Other contraceptive use data captured (n)[17]								X	X	X		X		X
Prior & concomitant medications	X	X*	X	X	X		X		X			X		X
Adverse events		X	X	X	X	X	X	X	X	X		X	X	X
Home pregnancy test dispensed[19]		X	X	X	X		X		X			X[6]		X
Blood Pregnancy Test [11]														X

V = Visit; C = call; SCRN = Screening; UNS = Unscheduled; Wks = Weeks; Mos = Months; VAR = Varies;

* If enrollment does not immediately follow the screening procedure, confirmation of eligibility must be performed and clinical judgment used to decide whether repeat pregnancy or other tests are needed and if contact information should be reviewed.

* Visit Windows: If enrollment does not occur on same day as screening, subject should return no later than 60 days from start of screening to be enrolled. Post insertion: Visit 2= 6 wks ± 5 days, Visit 3= 3 mos ± 14 days, Visit 4= 6 mos ± 14 days, Call 1 = 9 mos ± 14 days, Visit 5 = 12 mos ± 14 days, Call 2 = 18 mos ± 14 days, Visit 6 = 24 mos ± 14 days, Call 3 = 30 mos ± 14 days, Exit = 37 mos ± 14 days). Call 4 = 17 days (+3) post removal.

^ During unscheduled visits, the Investigator will perform any study assessments as necessary to provide appropriate treatment.

[1] Vital signs to include measurements of blood pressure, height, and weight.

[2] Pelvic exam at screening will assess for the presence of any uterine or cervical anomalies that would preclude IUD use.

[3] Next visit to be scheduled will be a phone call instead of an in-person office visit.

[4] Confirmation of IUD placement should be done initially by visual then palpable inspection to confirm presence of the IUD string. If the string is not present, or if there is a clinical suspicion of expulsion, a transvaginal ultrasound should be performed to confirm presence of IUD (Section 9.7, Appendix 2). Confirmation will be done by verbal patient report for the phone calls.

[5] If medically indicated, a pelvic exam, ultrasound and/or testing for chlamydia/gonorrhea can be performed at all visits. Refer to IUD Check Process outlined in Appendix 2

[6] Next visit to be scheduled during the Exit visit will be a phone call (to obtain results of in-home pregnancy test and follow up on AEs) instead of an in-person office visit.

[7] Endocervical, vaginal, or urine specimen collection is for Nucleic Acid Amplification Test (NAAT) for *Neisseria gonorrhoeae* and *Chlamydia trachomatis* (GC/CT). All subjects will be tested at screening.

[8] Urine pregnancy test will be completed in clinic at Screening and during Enrollment prior to the subject being randomized to confirm subject is not pregnant prior to IUD placement if Enrollment occurs on a separate day from Screening.
[9] During the Exit visit, the clinician will disclose which IUD the subject was using only after all IUD assessments are complete.
[10] If the subject is using ParaGard, ask if she wishes to continue. If not, remove IUD. If the subject is using the test product, remove IUD.
[11] Confirmatory quantitative blood pregnancy test should be done if initial urine pregnancy test comes back positive
[12] A screening Pap test will be completed for subjects at screening if potential subject is not in compliance with normal standard of care guidelines regarding Pap testing. If additional testing or treatment is required based on Pap results, this should be completed prior to enrollment.
[14] Visit 1 distribute 2 diary forms, Visit 2 distribute 2 diary forms, Visit 3 distribute 4 diary forms, and Visit 4 distribute 7 diary forms
[15] Visit 2 collect 2 diary forms, Visit 3 collect 2 diary forms, Visit 4 collect 4 diary forms and Visit 5 collect 7 diary forms
[16] Visit 5 and Visit 6 distribute 2 Other Contraceptive Use forms
[17] Call 2, Visit 6, Call 3, and Exit collect data from Other Contraceptive Use forms
[18] Includes subject’s levels for pain and bleeding
[19] Home pregnancy tests should be provided to subjects at Enrollment and then as needed at other visits with instructions for the subject to complete the test if there is any suspicion that they may be pregnant in between their study clinic visits.
[20] Included as part of the Follow-Up Form assessments. At Call 1, Call 2 and Call 3, the follow up form is also referred to as the Telephone Follow Up Form since the Follow Up form information is collected by telephone.
[21] Pain will be assessed immediately and 10 minutes after insertion

25 APPENDIX 2: IUD CONFIRMATION PROCESSES

In order to confirm IUD presence and placement, the following order of assessments should be completed until confirmation is completed. Once IUD placement is confirmed, no further assessment is needed regardless of which assessment confirmed placement (i.e. stop with first assessment if IUD placement is confirmed):

1. Visual inspection of cervix for presence of IUD strings or inspection with speculum
2. Digital palpation
3. Sweep for strings using cytobrush in endocervical canal
4. Transvaginal ultrasound (TVUS) examination
5. Abdominal X-ray to assess for location

Note: If IUD presence confirmation is only made using TVUS, TVUS should be performed at each subsequent study visit unless the string becomes visible again.

Also Note: These steps are meant to confirm IUD location at any time (not necessarily at placement). Additionally, either step 1 or 2 may be performed first. Visual inspection/ inspection with speculum does not have to be performed prior to digital palpation and vice versa. They can be performed interchangeably and not specifically in that order.

26 APPENDIX 3: DAILY DIARY

Note: The diary includes bleeding definitions to ensure consistent assessment of bleeding amongst study subjects. However, the definitions are based upon the use of tampons or pads. Subjects using menstrual cups should be instructed to use their best judgment to assess their level of bleeding each day since the bleeding definitions within the diary will not apply.

CCN016 IUD Daily Diary Start Date of 28 Day Version 01.00 2017 0509
Subject Number: interval: MMM-DD-YYYY

Bleeding	Date	MMM-DD	MMM-DD	MMM-DD	MMM-DD	MMM-DD	MMM-DD	MMM-DD	MMM-DD	MMM-DD	MMM-DD	MMM-DD	MMM-DD	MMM-DD	MMM-DD
	None	□	□	□	□	□	□	□	□	□	□	□	□	□	□
	Spotting	□	□	□	□	□	□	□	□	□	□	□	□	□	□
	Light bleeding	□	□	□	□	□	□	□	□	□	□	□	□	□	□
	Moderate bleeding	□	□	□	□	□	□	□	□	□	□	□	□	□	□
	Heavy bleeding	□	□	□	□	□	□	□	□	□	□	□	□	□	□
	**Diary Day**	**1**	**2**	**3**	**4**	**5**	**6**	**7**	**8**	**9**	**10**	**11**	**12**	**13**	**14**

Bleeding	Date	MMM-DD	MMM-DD	MMM-DD	MMM-DD	MMM-DD	MMM-DD	MMM-DD	MMM-DD	MMM-DD	MMM-DD	MMM-DD	MMM-DD	MMM-DD	MMM-DD
	None	□	□	□	□	□	□	□	□	□	□	□	□	□	□
	Spotting	□	□	□	□	□	□	□	□	□	□	□	□	□	□
	Light bleeding	□	□	□	□	□	□	□	□	□	□	□	□	□	□
	Moderate bleeding	□	□	□	□	□	□	□	□	□	□	□	□	□	□
	Heavy bleeding	□	□	□	□	□	□	□	□	□	□	□	□	□	□
	**Diary Day**	**15**	**16**	**17**	**18**	**19**	**20**	**21**	**22**	**23**	**24**	**25**	**26**	**27**	**28**

Did you have vaginal intercourse in this 28 day interval?	**No** □	**Yes** □
Did you use any other contraceptive method in this 28 day interval?	**No** □	**Yes** □
If any other contraceptive method was used, what method was used?	**Condom** □	**Other** □
If not a condom, specify method: ______________________________		

Dates will be prepopulated for you by the study staff. Complete bleeding information every day between your study visits. Provide responses on intercourse, contraceptive method and pain experienced for each full interval prior to finishing each diary form. Remember to bring the completed diary form back to your next visit. If you have any questions on how to complete the form, please ask the clinic staff.

Did you have any pelvic pain during this 28 day interval?	**No** □	**Yes** □	
If yes, was this pain during your period, outside of your period, or both during and outside your period?	**During period** □	**Outside of period** □	**Both during and outside of period** □

Bleeding definitions:
None – no bleeding or spotting
Spotting –if you have a minimal amount of vaginal bleeding and you used one panty liner all day or did not use any protection (panty liner, pad, tampon).
Light Bleeding – if you have vaginal bleeding and you have to use one or two pads or tampons, or more than one panty liner, throughout the day.
Moderate Bleeding – if you have vaginal bleeding and you have to use three or four pads or tampons throughout the day.
Heavy Bleeding – if you have a large amount of vaginal bleeding and you have to use many pads or tampons (more than 4) throughout the day
Note: If spotting and bleeding occur on the same day, record the heaviest bleeding choice applicable for that day.

27 APPENDIX 4: OTHER CONTRACEPTIVE USE FORM

CCN016 IUD - Other Contracpetive Use Form Version 01.00 2017 0509

Subject Number:

Interval dates will be prepopulated for you by the study staff. Provide respones to the questions during each period accordingly between your study visits. Remember to bring the completed form back to your next visit.

Start Date of 28 Day Interval:	DD-MMM-YYYY	
End Date of 28 Day Interval:	DD-MMM-YYYY	
Did you have vaginal intercourse in this 28 day interval?	**No** ☐	**Yes** ☐
Did you use any other contraceptive method in this 28 day interval?	**No** ☐	**Yes** ☐
If any other contraceptive method was used, what method was used?	**Condom** ☐	**Other** ☐
If not a condom, specify method:________________________________		

Start Date of 28 Day Interval:	DD-MMM-YYYY	
End Date of 28 Day Interval:	DD-MMM-YYYY	
Did you have vaginal intercourse in this 28 day interval?	**No** ☐	**Yes** ☐
Did you use any other contraceptive method in this 28 day interval?	**No** ☐	**Yes** ☐
If any other contraceptive method was used, what method was used?	**Condom** ☐	**Other** ☐
If not a condom, specify method:________________________________		

Start Date of 28 Day Interval:	DD-MMM-YYYY	
End Date of 28 Day Interval:	DD-MMM-YYYY	
Did you have vaginal intercourse in this 28 day interval?	**No** ☐	**Yes** ☐
Did you use any other contraceptive method in this 28 day interval?	**No** ☐	**Yes** ☐
If any other contraceptive method was used, what method was used?	**Condom** ☐	**Other** ☐
If not a condom, specify method:________________________________		

Start Date of 28 Day Interval:	DD-MMM-YYYY	
End Date of 28 Day Interval:	DD-MMM-YYYY	
Did you have vaginal intercourse in this 28 day interval?	**No** ☐	**Yes** ☐
Did you use any other contraceptive method in this 28 day interval?	**No** ☐	**Yes** ☐
If any other contraceptive method was used, what method was used?	**Condom** ☐	**Other** ☐
If not a condom, specify method:________________________________		

28 APPENDIX 5: POST PLACEMENT PAIN FORM – CLINIC

CCN016 Post Placement Pain Form – Clinic

Subject Number: ___________________ Date:____________________ (MMM-DD-YYYY)

Complete responses as instructed by clinic staff on day of IUD placement prior to leaving the visit.

**Baseline Measurement After Speculum Placement**	**Time:** _____________ AM □ PM □
Place a vertical mark on the line below that indicates how bad your pain was after speculum placement, but prior to IUD placement.	
No pain ____ Worst imaginable pain	

**IUD Placement:**	**Time:** _____________ AM □ PM □

**Immediately Post IUD Placement Measurement**	**Time:** _____________ AM □ PM □
Place a vertical mark on the line below that indicates how bad your pain was immediately after IUD placement.	
No pain ____ Worst imaginable pain	

**10 Minutes Post IUD Placement Measurement**	**Time:** _____________ AM □ PM □
Place a vertical mark on the line below that indicates how bad your pain was 10 minutes after IUD placement.	
No pain ____ Worst imaginable pain	

Date:____________________ (MMM-DD-YYYY)

Complete responses as instructed by clinic staff on day of IUD removal prior to leaving the visit.

**Immediately Post IUD Removal Measurement**	**Time:** _____________ AM □ PM □
Place a vertical mark on the line below that indicates how bad your pain was immediately after IUD removal.	
No pain ____ Worst imaginable pain	

Version 01.00 2017 0509

29 APPENDIX 6: POST PLACEMENT PAIN FORM – HOME

CCN016 Post Placement Pain Form – Home Subject Number: ___________________

Complete responses each day prior to going to bed for the 6 consecutive days after your IUD was placed.

**1 Day Post Placement**	**Date:** ____________________(MMM-DD-YYYY)
Place a vertical mark on the line below that indicates the worst level of pain that you experienced today.	
No pain ____ Worst imaginable pain	

**2 Days Post Placement**	**Date:** ____________________(MMM-DD-YYYY)
Place a vertical mark on the line below that indicates the worst level of pain that you experienced today.	
No pain ____ Worst imaginable pain	

**3 Days Post Placement**	**Date:** ____________________(MMM-DD-YYYY)
Place a vertical mark on the line below that indicates the worst level of pain that you experienced today.	
No pain ____ Worst imaginable pain	

**4 Days Post Placement**	**Date:** ____________________(MMM-DD-YYYY)
Place a vertical mark on the line below that indicates the worst level of pain that you experienced today.	
No pain ____ Worst imaginable pain	

**5 Days Post Placement**	**Date:** ____________________(MMM-DD-YYYY)
Place a vertical mark on the line below that indicates the worst level of pain that you experienced today.	
No pain ____ Worst imaginable pain	

**6 Days Post Placement**	**Date:** ____________________(MMM-DD-YYYY)
Place a vertical mark on the line below that indicates the worst level of pain that you experienced today.	
No pain ____ Worst imaginable pain	
How satisfied are you with the IUD? Highly Satisfied □ Satisfied □ Dissatisfied □ Highly Dissatisfied □	
Would you recommend this IUD to others? No □ Yes □	
For how many days after your IUD placement did you take pain medications for the specific purpose of controlling pain related to the IUD placement? 0 □ 1-2 □ 3-5 □ 6 or more □	

Version 01.00 2017 0509

Confidential

30 APPENDIX 7: PARAGARD PRESCRIBING INFORMATION

ParaGard® T 380A
INTRAUTERINE COPPER CONTRACEPTIVE

P/N 1017005
Rev. 9/2014

Rx only

PRESCRIBING INFORMATION

ParaGard® T 380A Intrauterine Copper Contraceptive

Patients should be counseled that this product does not protect against HIV infection (AIDS) and other sexually transmitted diseases.

ParaGard® T 380A Intrauterine Copper Contraceptive should be placed and removed only by healthcare professionals who are experienced with these procedures.

DESCRIPTION

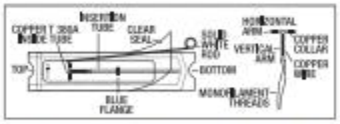

ParaGard® T 380A Intrauterine Copper Contraceptive (ParaGard®) is a T-shaped intrauterine device (IUD), measuring 32 mm horizontally and 36 mm vertically, with a 3 mm diameter bulb at the tip of the vertical stem. A monofilament polyethylene thread is tied through the tip, resulting in two white threads, each at least 10.5 cm in length, to aid in detection and removal of the device. The T-frame is made of polyethylene with barium sulfate to aid in detecting the device under x-ray. ParaGard® also contains copper: approximately 176 mg of wire coiled along the vertical stem and a 68.7 mg collar on each side of the horizontal arm. The total exposed copper surface area is 380 ± 23 mm². One ParaGard® weighs less than one (1) gram. No component of ParaGard® or its packaging contains latex.
ParaGard® is packaged together with an insertion tube and solid white rod in a Tyvek® polyethylene pouch that is then sterilized. A moveable flange on the insertion tube aids in gauging the depth of insertion through the cervical canal and into the uterine cavity.

CLINICAL PHARMACOLOGY
The contraceptive effectiveness of ParaGard® is enhanced by copper continuously released into the uterine cavity. Mechanism(s) by which copper enhances contraceptive efficacy include interference with sperm transport and fertilization of an egg, and possibly prevention of implantation.

INDICATIONS AND USAGE
ParaGard® is indicated for intrauterine contraception for up to 10 years. The pregnancy rate in clinical studies has been less than 1 pregnancy per 100 women each year.

Table 1: Percentage of women experiencing an unintended pregnancy during the first year of typical use and first year of perfect use of contraception and the percentage continuing use at the end of the first year: United States

	% of Women Experiencing an Accidental Pregnancy within the First Year of Use		% of Women Continuing Use at One Year[3]
**Method (1)**	**Typical Use[1] (2)**	**Perfect Use[2] (3)**	**(4)**
Chance[4]	85	85	
Spermicides[5]	26	6	40
Periodic Abstinence<br>Calendar<br>Ovulation Method<br>Sympto-thermal[6]<br>Post-ovulation	25	 9<br>3<br>2<br>1	63
Cap[7]<br>Parous women<br>Nulliparous women	 40<br>20	 26<br>9	 42<br>56
Sponge<br>Parous women<br>Nulliparous women	 40<br>20	 20<br>9	 42<br>56
Diaphragm[7]	20	6	56
Withdrawal	19	4	
Condom[8]<br>Female (Reality)<br>Male	 21<br>14	 5<br>3	 56<br>61
Pill<br>Progestin only<br>Combined	5	 0.5<br>0.1	71
IUD<br>Progesterone T<br>Copper T 380A<br>LNg 20	 2.0<br>0.8<br>0.1	 1.5<br>0.6<br>0.1	 81<br>78<br>81
Depo Provera	0.3	0.3	70
Norplant<br>and Norplant-2	0.05	0.05	88
Female sterilization	0.5	0.5	100
Male sterilization	0.15	0.10	100

1

ParaGard® T 380A Intrauterine Copper Contraceptive

Emergency Contraceptive Pills: Treatment initiated within 72 hours after unprotected intercourse reduces the risk of pregnancy by at least 75%.[9]

Lactational Amenorrhea Method: LAM is a highly effective temporary method of contraception.[10]

Footnotes to Table 1
Source: Trussell J, Contraceptive efficacy. In Hatcher RA, Trussell J, Stewart F, Cates W, Stewart GK, Kowal D, Guest F, Contraceptive Technology: Seventeenth Revised Edition. New York NY: Irvington Publishers, 1998.

1. Among typical couples who initiate use of a method (not necessarily for the first time), the percentage who experience an accidental pregnancy during the first year if they do not stop use for any other reason.
2. Among couples who initiate use of a method (not necessarily for the first time) and who use it perfectly (both consistently and correctly), the percentage who experience an accidental pregnancy during the first year if they do not stop use for any reason.
3. Among couples attempting to avoid pregnancy, the percentage who continue to use a method for one year.
4. The percents becoming pregnant in columns (2) and (3) are based on data from populations where contraception is not used and from women who cease using contraception in order to become pregnant. Among such populations, about 89% become pregnant within one year. This estimate was lowered slightly (to 85%) to represent the percentage who would become pregnant within one year among women now relying on reversible methods of contraception if they abandoned contraception altogether.
5. Foams, creams, gels, vaginal suppositories, and vaginal film.
6. Cervical mucus (ovulation) method supplemented by calendar in the pre-ovulatory and basal body temperature in the post-ovulatory phases.
7. With spermicidal cream or jelly.
8. Without spermicides.
9. The treatment schedule is one dose within 72 hours after unprotected intercourse, and a second dose 12 hours after the first dose. Preven is the only dedicated product specifically marketed for emergency contraception. The Food and Drug Administration has also declared the following brands of oral contraceptive to be safe and effective for emergency contraception: Ovral (1 dose is 2 white pills), Alesse (1 dose is 5 pink pills), Nordette or Levlen (1 dose is 4 light-orange pills), Lo/Ovral (1 dose is 4 white pills), Triphasil or Tri-Levlen (1 dose is 4 yellow pills).
10. However, to maintain effective protection against pregnancy, another method of contraception must be used as soon as menstruation resumes, the frequency or duration of breastfeeds is reduced, bottle feeds are introduced or the baby reaches 6 months of age.

CONTRAINDICATIONS
ParaGard® should not be placed when one or more of the following conditions exist:
1. Pregnancy or suspicion of pregnancy
2. Abnormalities of the uterus resulting in distortion of the uterine cavity
3. Acute pelvic inflammatory disease, or current behavior suggesting a high risk for pelvic inflammatory disease
4. Postpartum endometritis or postabortal endometritis in the past 3 months
5. Known or suspected uterine or cervical malignancy
6. Genital bleeding of unknown etiology
7. Mucopurulent cervicitis
8. Wilson's disease
9. Allergy to any component of ParaGard®
10. A previously placed IUD that has not been removed

WARNINGS
1. Intrauterine Pregnancy
If intrauterine pregnancy occurs with ParaGard® in place and the string is visible, ParaGard® should be removed because of the risk of spontaneous abortion, premature delivery, sepsis, septic shock, and, rarely, death. Removal may be followed by pregnancy loss.
If the string is not visible, and the woman decides to continue her pregnancy, check if the ParaGard® is in her uterus (for example, by ultrasound). If ParaGard® is in her uterus, warn her that there is an increased risk of spontaneous abortion and sepsis, septic shock, and rarely, death.[1] In addition, the risk of premature labor and delivery is increased.[1]
Human data about risk of birth defects from copper exposure are limited. However, studies have not detected a pattern of abnormalities, and published reports do not suggest a risk that is higher than the baseline risk for birth defects.

2. Ectopic Pregnancy
Women who become pregnant while using ParaGard® should be evaluated for ectopic pregnancy. A pregnancy that occurs with ParaGard® in place is more likely to be ectopic than a pregnancy in the general population. However, because ParaGard® prevents most pregnancies, women who use ParaGard® have a lower risk of an ectopic pregnancy than sexually active women who do not use any contraception.[2-3]

3. Pelvic Infection
Although pelvic inflammatory disease (PID) in women using IUDs is uncommon, IUDs may be associated with an increased relative risk of PID compared to other forms of contraception and to no contraception. The highest incidence of PID occurs within 20 days following insertion. Therefore, the visit following the first post-insertion menstrual period is an opportunity to assess the patient for infection, as well as to check that the IUD is in place. (See **INSTRUCTIONS FOR USE, Continuing Care.**) Since pelvic infection is most frequently associated with sexually transmitted organisms, IUDs are not recommended for women at high risk for sexual infection. Prophylactic antibiotics at the time of insertion do not appear to lower the incidence of PID.[4]

PID can have serious consequences, such as tubal damage (leading to ectopic pregnancy or infertility), hysterectomy, sepsis, and, rarely, death. It is therefore important to promptly assess and treat any woman who develops signs or symptoms of PID. Guidelines for treatment of PID are available from the Centers for Disease Control and Prevention (CDC), Atlanta, Georgia at www.cdc.gov or 1-800-311-3435. Antibiotics are the mainstay of therapy. Most healthcare professionals also remove the IUD.

The significance of actinomyces-like organisms on Papanicolaou smear in an asymptomatic IUD user is unknown,[5-6] and so this finding alone does not always require IUD removal and treatment. However, because pelvic actinomycosis is a serious infection, a woman who has *symptoms* of pelvic infection possibly due to actinomyces should be treated and have her IUD removed.

4. Immunocompromise
Women with AIDS should not have IUDs inserted unless they are clinically stable on antiretroviral therapy. Limited data suggest that asymptomatic women infected with human immunodeficiency virus may use intrauterine devices. Little is known about the use of IUDs in women who have illnesses causing serious immunocompromise. Therefore these women should be carefully monitored for infection if they choose to use an IUD. The risk of pregnancy should be weighed against the theoretical risk of infection.

5. Embedment
Partial penetration or embedment of ParaGard® in the myometrium can make removal difficult. In some cases, surgical removal may be necessary.

6. Perforation
Partial or total perforation of the uterine wall or cervix may occur rarely during placement, although it may not be detected until later. Spontaneous migration has also been reported. If perforation does occur, remove ParaGard® promptly, since the copper can lead to intraperitoneal adhesions. Intestinal penetration, intestinal obstruction, and/or damage to adjacent organs may result if an IUD is left in the peritoneal cavity. Pre-operative imaging followed by laparoscopy or laparotomy is often required to remove an IUD from the peritoneal cavity.

7. Expulsion
Expulsion can occur, usually during the menses and usually in the first few months after insertion. There is an increased risk of expulsion in the nulliparous patient. If unnoticed, an unintended pregnancy could occur.

8. Wilson's Disease
Theoretically, ParaGard® can exacerbate Wilson's disease, a rare genetic disease affecting copper excretion.

PRECAUTIONS

Patients should be counseled that this product does not protect against HIV infection (AIDS) and other sexually transmitted diseases.

1. Information for patients
Before inserting ParaGard® discuss the Patient Package Insert with the patient, and give her time to read the information. Discuss any questions she may have concerning ParaGard® as well as other methods of contraception. Instruct her to promptly report symptoms of infection, pregnancy, or missing strings.

2. Insertion precautions, continuing care, and removal.
(See **INSTRUCTIONS FOR USE**.)

3. Vaginal bleeding
In the 2 largest clinical trials with ParaGard® (see ADVERSE REACTIONS, Table 2), menstrual changes were the most common medical reason for discontinuation of ParaGard®. Discontinuation rates for pain and bleeding combined are highest in the first year of use and diminish thereafter. The percentage of women who discontinued ParaGard® because of bleeding problems or pain during these studies ranged from 11.9% in the first year to 2.2 % in year 9. Women complaining of heavy vaginal bleeding should be evaluated and treated, and may need to discontinue ParaGard®. (See **ADVERSE REACTIONS**.)

4. Vasovagal reactions, including fainting
Some women have vasovagal reactions immediately after insertion. Hence, patients should remain supine until feeling well and should be cautious when getting up.

5. Expulsion following placement after a birth or abortion
ParaGard® has been placed immediately after delivery, although risk of expulsion may be higher than when ParaGard® is placed at times unrelated to delivery.[7] However, unless done immediately postpartum, insertion should be delayed to the second postpartum month because insertion during the first postpartum month (except for immediately after delivery) has been associated with increased risk of perforation.[8]

ParaGard® can be placed immediately after abortion, although immediate placement has a slightly higher risk of expulsion than placement at other times.[9] Placement after second trimester abortion is associated with a higher risk of expulsion than placement after the first trimester abortion.[9]

6. Magnetic resonance imaging (MRI)
Limited data suggest that MRI at the level of 1.5 Tesla is acceptable in women using ParaGard®. One study examined the effect of MRI on the CU-7® Intrauterine Copper Contraceptive and Lippes Loop™ intrauterine devices. Neither device moved under the influence of the magnetic field or heated during the spin-echo sequences usually employed for pelvic imaging.[10] An in vitro study did not detect movement or temperature change when ParaGard® was subjected to MRI.[11]

7. Medical diathermy
Theoretically, medical (non-surgical) diathermy (short-wave and microwave heat therapy) in a patient with a metal-containing IUD may cause heat injury to the surrounding tissue. However, a small study of eight women did not detect a significant elevation of intrauterine temperature when diathermy was performed in the presence of a copper IUD.[12]

8. Pregnancy
ParaGard® is contraindicated during pregnancy. (See CONTRAINDICATIONS and WARNINGS.)

9. Nursing mothers
Nursing mothers may use ParaGard®. No difference has been detected in concentration of copper in human milk before and after insertion of copper IUDs. The literature is conflicting, but limited data suggest that there may be an increased risk of perforation and expulsion if a woman is lactating.[13]

10. Pediatric use
ParaGard® is not indicated before menarche. Safety and efficacy have been established in women over 16 years old.

ADVERSE REACTIONS

The most serious adverse events associated with intrauterine contraception are discussed in **WARNINGS** and **PRECAUTIONS**. These include:

Intrauterine pregnancy	Pelvic infection
Septic abortion	Perforation
Ectopic pregnancy	Embedment

Table 2 shows discontinuation rates from two clinical studies by adverse event and year.

Table 2. Summary of Rates (No. per 100 Subjects) by Year for Adverse Events Causing Discontinuation

Adverse Event	Year									
	1	2	3	4	5	6	7	8	9	10
**Pregnancy**	0.7	0.3	0.6	0.2	0.3	0.2	0.0	0.4	0.0	0.0
**Expulsion**	5.7	2.5	1.6	1.2	0.3	0.0	0.6	1.7	0.2	0.4
**Bleeding/Pain**	11.9	9.8	7.0	3.5	3.7	2.7	3.0	2.5	2.2	3.7
**Other Medical Event**	2.5	2.1	1.6	1.7	0.1	0.3	1.0	0.4	0.7	0.3
**No. of Women at Start of Year**	4932	3149	2018	1121	872	621	563	483	423	325

*Rates were calculated by weighting the annual rates by the number of subjects starting each year for each of the Population Council (3,536 subjects) and the World Health Organization (1,396 subjects) trials.

The following adverse events have also been observed. These are listed alphabetically and not by order of frequency or severity.

Anemia	Menstrual flow, prolonged
Backache	Menstrual spotting
Dysmenorrhea	Pain and cramping
Dyspareunia	Urticarial allergic skin reaction
Expulsion, complete or partial	Vaginitis
Leukorrhea	

INSTRUCTIONS FOR USE

The placement technique for ParaGard® is different from that used for other IUDs. Therefore, the clinician should be familiar with the following instructions.

ParaGard® may be placed at any time during the cycle when the clinician is reasonably certain the patient is not pregnant. For information about timing of postpartum and postabortion insertions, see **PRECAUTIONS**.

A single ParaGard® should be placed at the fundus of the uterine cavity. ParaGard® should be removed on or before 10 years from the date of insertion.

Before Placement:

1. Make sure that the patient is an appropriate candidate for ParaGard® and that she has read the Patient Package Insert.
2. Use of an analgesic before insertion is at the discretion of the patient and the clinician.
3. Establish the size and position of the uterus by pelvic examination.
4. Insert a speculum and cleanse the vagina and cervix with an antiseptic solution.
5. Apply a tenaculum to the cervix and use gentle traction to align the cervical canal with the uterine cavity.
6. Gently insert a sterile sound to measure the depth of the uterine cavity.

7. The uterus should sound to a depth of 6 to 9 cm except when inserting ParaGard® immediately post-abortion or post-partum. Insertion of ParaGard® into a uterine cavity measuring less than 6 cm may increase the incidence of expulsion, bleeding, pain, and perforation. If you encounter cervical stenosis, avoid undue force. Dilators may be helpful in this situation.

How to Load and Place ParaGard®:
Do not bend the arms of ParaGard® earlier than 5 minutes before it is to be placed in the uterus. Use aseptic technique when handling ParaGard® and the part of the insertion tube that will enter the uterus.

STEP 1
Load ParaGard® into the insertion tube by folding the two horizontal arms of ParaGard® against the stem and push the tips of the arms securely into the inserter tube.
If you do not have sterile gloves, you can do STEPS 1 and 2 while ParaGard® is in the sterile package. First, place the package face up on a clean surface. Next, open at the bottom end (where arrow says OPEN). Pull the solid white rod partially from the package so it will not interfere with assembly. Place thumb and index finger on top of package on ends of the horizontal arms. Use other hand to push insertion tube against arms of ParaGard® (shown by arrow in Fig. 1). This will start bending the T arms.

Fig. 1

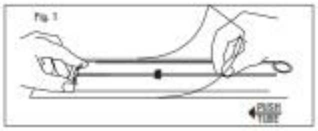

STEP 2
Bring the thumb and index finger closer together to continue bending the arms until they are alongside the stem. Use the other hand to withdraw the insertion tube just enough so that the insertion tube can be pushed and rotated onto the tips of the arms. Your goal is to secure the tips of the arms inside the tube (Fig. 2). Insert the arms no further than necessary to insure retention. Introduce the solid white rod into the insertion tube from the bottom, alongside the threads, **until it touches the bottom of the ParaGard®**.

Fig. 2

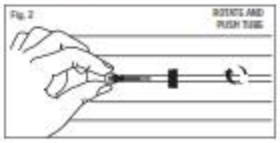

STEP 3
Grasp the insertion tube at the open end of the package; adjust the blue flange so that the distance from the top of the ParaGard® (where it protrudes from the inserter) to the blue flange is the same as the uterine depth that you measured with the sound. Rotate the insertion tube so that the horizontal arms of the T and the long axis of the blue flange lie in the same horizontal plane (Fig. 3). Now pass the loaded insertion tube through the cervical canal until ParaGard® just touches the fundus of the uterus. The blue flange should be at the cervix in the horizontal plane.

Fig. 3

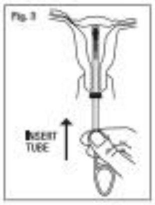

STEP 4
To release the arms of ParaGard®, hold the solid white rod steady and withdraw the insertion tube no more than one centimeter. This releases the arms of ParaGard® high in the uterine fundus (Fig. 4).

Fig. 4

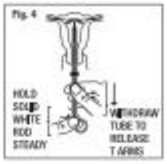

ParaGard® T 380A Intrauterine Copper Contraceptive

STEP 5
Gently and carefully move the insertion tube upward toward the top of the uterus, until slight resistance is felt. This will ensure placement of the T at the highest possible position within the uterus (Fig. 5).

Fig. 5

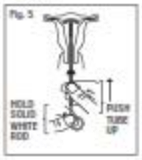

STEP 6
Hold the insertion tube steady and withdraw the solid white rod (Fig. 6).

Fig. 6

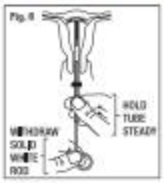

STEP 7
Gently and slowly withdraw the insertion tube from the cervical canal. Only the threads should be visible protruding from the cervix. (Fig. 7). Trim the threads so that 3 to 4 cm protrude into the vagina. Note the length of the threads in the patient's records.

Fig. 7

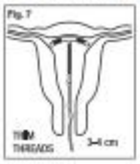

If you suspect that ParaGard® is not in the correct position, check placement (with ultrasound, if necessary). If ParaGard® is not positioned completely within the uterus, remove it and replace it with a new ParaGard®. Do not reinsert an expelled or partially expelled ParaGard®.

CAUTION
Instrumentation of the cervical os may result in vasovagal reactions, including fainting. Have the patient remain supine until she feels well, and have her get up with caution.

Continuing Care:
Following placement, examine the patient after her first menses to confirm that ParaGard® is still in place. **You should be able to see or feel only the threads.** If ParaGard® has been partially or completely expelled, remove it. You can place a new ParaGard® if the patient desires and if she is not pregnant. Do not reinsert a used ParaGard®.
Evaluate the patient promptly if she complains of any of the following:
- Abdominal or pelvic pain, cramping, or tenderness; malodorous discharge; bleeding; fever
- A missed period

(See **WARNINGS, Pelvic Infection, Intrauterine Pregnancy** and **Ectopic Pregnancy**.)
The length of the visible threads may change with time. However, no action is needed unless you suspect partial expulsion, perforation, or pregnancy.
If you cannot find the threads in the vagina, check that ParaGard® is still in the uterus. The threads can retract into the uterus or break, or ParaGard® can break, perforate the uterus, or be expelled. Gentle probing of the cavity, radiography, or sonography may be required to locate the IUD.
If there is evidence of partial expulsion, perforation, or breakage, remove ParaGard®.

How to Remove ParaGard®
Remove ParaGard® with forceps, pulling gently on the exposed threads. The arms of ParaGard® will fold upwards as it is withdrawn from the uterus. You may immediately insert a new ParaGard® if the patient requests it and has no contraindications.
Embedment or breakage of ParaGard® in the myometrium can make removal difficult. Analgesia, paracervical anesthesia, and cervical dilation may assist in removing an embedded ParaGard®. An alligator forceps or other grasping instrument may be helpful. Hysteroscopy may also be helpful.

HOW SUPPLIED
ParaGard® is available in cartons of 1 (one) sterile unit (NDC 51285-204-01). Each ParaGard® is packaged together with an insertion tube and solid white rod in a Tyvek® polyethylene pouch.

REFERENCES

1. Tatum HJ, Schmidt FH, Jain AK. Management and outcome of pregnancies associated with the Copper T intrauterine contraceptive device. *Am J Obstet Gynecol.* 1976;126:869-879.
2. Sivin I. Dose- and age-dependent ectopic pregnancy risks with intrauterine contraception. *Obstet Gynecol.* 1991;78:291-298.
3. Franks AL, Beral V, Cates W Jr, Hogue CJR. Contraception and ectopic pregnancy risk. *Am J Obstet Gynecol.* 1990;163:1120-1123.
4. Grimes DA, Schulz KF. Prophylactic antibiotics for intrauterine device insertion: a meta-analysis of the randomized controlled trials. *Contraception.* 1999;60:57-63.
5. Lippes J. Pelvic actinomycosis: a review and preliminary look at prevalence. *Am J Obstet Gynecol.* 1999;180:265-269.
6. Petitti DB, Yamamoto D, Morgenstern N. Factors associated with actinomyces-like organisms on Papanicolaou smear in users of intrauterine contraceptive devices. *Am J Obstet Gynecol.* 1983;145:338-341.
7. Grimes D, Schulz K, van Vliet H, Stanwood N. Immediate post-partum insertion of intrauterine devices: a Cochrane review. *Hum Reprod.* 2002;17:549-554.
8. Cole LP, Edelman DA, Potts DM, Wheeler RG, Laufe LE. Postpartum insertion of modified intrauterine devices. *J Reprod Med.* 1984;29:677-682.
9. Grimes DA, Schulz KF, Stanwood N. Immediate post-abortal insertion of intrauterine devices. (Cochrane Review). In: *The Cochrane Library,* Issue 2, 2003. Oxford: Update Software.
10. Hess T, Stepanow B, Knopp MV. Magnetic resonance imaging: safety of intrauterine contraceptive devices during MR imaging. *Eur Radiol.* 1996;6:66-68.
11. Mark AS, Hricak H. Intrauterine contraceptive devices: MR imaging. *Radiology.* 1987;162:311-314.
12. Heick A., Espersen T., Pedersen HL, Raahauge J: Is diathermy safe in women with copper-bearing IUDs? *Acta Obstet Gynecol Scand.* 1991;70(2):153-5.
13. Rodrigues da Cunha AC, Dorea JG, Cantuaria AA. Intrauterine device and maternal copper metabolism during lactation. Contraception 2001;63:37-9.

INFORMATION FOR PATIENTS

ParaGard® T 380A Intrauterine Copper Contraceptive

ParaGard® T 380A Intrauterine Copper Contraceptive is used to prevent pregnancy. It does not protect against HIV infection (AIDS) and other sexually transmitted diseases.

It is important for you to understand this brochure and discuss it with your healthcare provider before choosing ParaGard® T 380A Intrauterine Copper Contraceptive (ParaGard®). You should also learn about other birth control methods that may be an option for you.

What is ParaGard®?

ParaGard® is a copper-releasing device that is placed in your uterus to prevent pregnancy for up to 10 years.

ParaGard® is made of white plastic in the shape of a "T." Copper is wrapped around the stem and arms of the "T". Two white threads are attached to the stem of the "T". The threads are the only part of ParaGard® that you can feel when ParaGard® is in your uterus. ParaGard® and its components do not contain latex.

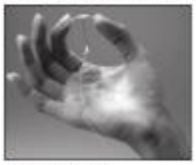

How long can I keep ParaGard® in place?

You can keep ParaGard® in your uterus for up to 10 years. After 10 years, you should have ParaGard® removed by your healthcare provider. If you wish and if it is still right for you, you may get a new ParaGard® during the same visit.

What if I change my mind and want to become pregnant?

Your healthcare provider can remove ParaGard® at any time. After discontinuation of ParaGard®, its contraceptive effect is reversed.

How does ParaGard® work?

Ideas about how ParaGard® works include preventing sperm from reaching the egg, preventing sperm from fertilizing the egg, and possibly preventing the egg from attaching (implanting) in the uterus. ParaGard® does not stop your ovaries from making an egg (ovulating) each month.

How well does ParaGard® work?

Fewer than 1 in 100 women become pregnant each year while using ParaGard®.

The table below shows the chance of getting pregnant using different types of birth control. The numbers show *typical* use, which includes people who don't always use birth control correctly.

ParaGard® T 380A Intrauterine Copper Contraceptive

Number of women out of 100 women who are likely to get pregnant over one year

Method of birth control	Pregnancies per 100 women over one year
No Method	85
Spermicides	26
Periodic abstinence	25
Cap with Spermicides	20
Vaginal Sponge	20 to 40
Diaphragm with Spermicides	20
Withdrawal	19
Condom without spermicides (female)	21
Condom without spermicides (male)	14
Oral Contraceptives	5
IUDs, Depo-Provera, implants, sterilization	less than 1

Who might use ParaGard®?

You might choose ParaGard® if you

- need birth control that is very effective
- need birth control that stops working when you stop using it
- need birth control that is easy to use

Who should not use ParaGard®?

You should not use ParaGard® if you

- Might be pregnant
- Have a uterus that is abnormally shaped inside
- Have a pelvic infection called pelvic inflammatory disease (PID) or have current behavior that puts you at high risk of PID (for example, because you are having sex with several men, or your partner is having sex with other women)
- Have had an infection in your uterus after a pregnancy or abortion in the past 3 months
- Have cancer of the uterus or cervix
- Have unexplained bleeding from your vagina
- Have an infection in your cervix
- Have Wilson's disease (a disorder in how the body handles copper)
- Are allergic to anything in ParaGard®
- Already have an intrauterine contraceptive in your uterus

How is ParaGard® placed in the uterus?

ParaGard® is placed in your uterus during an office visit. Your healthcare provider first examines you to find the position of your uterus. Next, he or she will cleanse your vagina and cervix, measure your uterus, and then slide a plastic tube containing ParaGard® into your uterus. The tube is removed, leaving ParaGard® inside your uterus. Two white threads extend into your vagina. The threads are trimmed so they are just long enough for you to feel with your fingers when doing a self-check. As ParaGard® goes in, you may feel cramping or pinching. Some women feel faint, nauseated, or dizzy for a few minutes afterwards. Your healthcare provider may ask you to lie down for a while and to get up slowly.

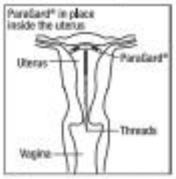

How do I check that ParaGard® is in my uterus?

Visit your healthcare provider for a check-up about one month after placement to make sure ParaGard® is still in your uterus.

You can also check to make sure that ParaGard® is still in your uterus by reaching up to the top of your vagina with clean fingers to feel the two threads. Do not pull on the threads.

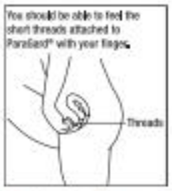

If you cannot feel the threads, ask your healthcare provider to check if ParaGard® is in the right place. If you can feel more of ParaGard® than just the threads, ParaGard® is *not* in the right place. If you can't see your healthcare provider right away, use an additional birth control method. If ParaGard® is in the wrong place, your chances of getting pregnant are increased. It is a good habit for you to check that ParaGard® is in place once a month.

You may use tampons when you are using ParaGard®.

What if I become pregnant while using ParaGard®?

If you think you are pregnant, contact your healthcare professional *right away*. If you are pregnant and ParaGard® is in your uterus, you may get a severe infection or shock, have a miscarriage or premature labor and delivery, or even die. Because of these risks, your healthcare provider will recommend that you have ParaGard® removed, even though removal may cause miscarriage.

If you continue a pregnancy with ParaGard® in place, see your healthcare provider regularly. Contact your healthcare provider right away if you get fever, chills, cramping, pain, bleeding, flu-like symptoms, or an unusual, bad smelling vaginal discharge. A pregnancy with ParaGard® in place has a greater than usual chance of being ectopic (outside your uterus). Ectopic pregnancy is an emergency that may require surgery. An ectopic pregnancy can cause internal bleeding, infertility, and death. Unusual vaginal bleeding or abdominal pain may be signs of an ectopic pregnancy. Copper in ParaGard® does not seem to cause birth defects.

What side effects can I expect with ParaGard®?

The most common side effects of ParaGard® are heavier, longer periods and spotting between periods; most of these side effects diminish after 2-3 months. However, if your menstrual flow continues to be heavy or long, or spotting continues, contact your healthcare provider.

Infrequently, serious side effects may occur:

- Pelvic inflammatory disease (PID): Uncommonly, ParaGard® and other IUDs are associated with PID. PID is an infection of the uterus, tubes, and nearby organs. PID is most likely to occur in the first 20 days after placement. You have a higher chance of getting PID if you or your partner have sex with more than one person. PID is treated with antibiotics. However, PID can cause serious problems such as infertility, ectopic pregnancy, and chronic pelvic pain. Rarely, PID may even cause death. More serious cases of PID require surgery or a hysterectomy (removal of the uterus). Contact your healthcare provider right away if you have any of the signs of PID: abdominal or pelvic pain, painful sex, unusual or bad smelling vaginal discharge, chills, heavy bleeding, or fever.
- Difficult removals: Occasionally ParaGard® may be hard to remove because it is stuck in the uterus. Surgery may sometimes be needed to remove ParaGard®.
- Perforation: Rarely, ParaGard® goes through the wall of the uterus, especially during placement. This is called perforation. If ParaGard® perforates the uterus, it should be removed. Surgery may be needed. Perforation can cause infection, scarring, or damage to other organs. If ParaGard® perforates the uterus, you are not protected from pregnancy.
- Expulsion: ParaGard® may partially or completely fall out of the uterus. This is called expulsion. Women who have never been pregnant may be more likely to expel ParaGard® than women who have been pregnant before. If you think that ParaGard® has partly or completely fallen out, use an additional birth control method, such as a condom and call your healthcare provider.

You may have other side effects with ParaGard®. For example, you may have anemia (low blood count), backache, pain during sex, menstrual cramps, allergic reaction, vaginal infection, vaginal discharge, faintness, or pain. This is not a complete list of possible side effects. If you have questions about a side effect, check with your healthcare provider.

When should I call my healthcare provider?

Call your healthcare provider if you have any concerns about ParaGard®. Be sure to call if you

- Think you are pregnant
- Have pelvic pain or pain during sex
- Have unusual vaginal discharge or genital sores
- Have unexplained fever
- Might be exposed to sexually transmitted diseases (STDs)
- Cannot feel ParaGard®'s threads or can feel the threads are much longer
- Can feel any other part of the ParaGard® besides the threads
- Become HIV positive or your partner becomes HIV positive
- Have severe or prolonged vaginal bleeding
- Miss a menstrual period

General advice about prescription medicines

This brochure summarizes the most important information about ParaGard®. If you would like more information, talk with your healthcare provider. You can ask your healthcare provider for information about ParaGard® that is written for healthcare professionals.

Checklist

This checklist will help you and your healthcare provider discuss the pros and cons of ParaGard® for you. Do you have any of the following conditions?

	Yes	No	Don't know
Abnormal Pap smear	☐	☐	☐
Abnormalities of the uterus	☐	☐	☐
Allergy to copper	☐	☐	☐
Anemia or blood clotting problems	☐	☐	☐
Bleeding between periods	☐	☐	☐
Cancer of the uterus or cervix	☐	☐	☐
Fainting attacks	☐	☐	☐

ParaGard® T 380A Intrauterine Copper Contraceptive

continued	Yes	No	Don't know
Genital sores	☐	☐	☐
Heavy menstrual flow	☐	☐	☐
HIV or AIDS	☐	☐	☐
Infection of the uterus or cervix	☐	☐	☐
IUD in place now or in the past	☐	☐	☐
More than one sexual partner	☐	☐	☐
Pelvic infection (PID)	☐	☐	☐
Possible pregnancy	☐	☐	☐
Repeated episodes of pelvic infection (PID)	☐	☐	☐
Serious infection following a pregnancy or abortion in the past 3 months	☐	☐	☐
Severe menstrual cramps	☐	☐	☐
Sexual partner who has more than one sexual partner	☐	☐	☐
Sexually transmitted disease (STD) such as gonorrhea or chlamydia	☐	☐	☐
Wilson's disease	☐	☐	☐

Teva Women's Health, Inc.
A Subsidiary of Teva Pharmaceuticals USA, Inc.
North Wales, PA 19454

Rev. 9/2014

P/N 1017005

PAR-40817

31 APPENDIX 8: MONA LISA NT CU380 MINI PRESCRIBING INFORMATION

Neo-*Safe*® T CU 380 / CU 380 Mini

Intrauterine Contraceptive Device

(EN)

Neo-*Safe*® T CU 380
Neo-*Safe*® T CU 380 Mini

PHYSICIAN INSTRUCTIONS

Presentation

The Neo-Safe® T CU 380 / Neo-Safe® T CU 380 Mini Intrauterine Device (IUD) is made of polyethylene and barium sulphate. Copper wire is wound around the vertical arm. The copper surface is 380 mm².

Dimension:

The Neo-Safe® T CU 380 is 31,8 mm ± 0,2 mm wide and 31,9 mm ± 0,2 mm long.

The Neo-Safe® T CU 380 Mini is 24,0 mm ± 0,2 mm wide and 30,0 mm ± 0,2 mm long.

The polyethylene frame and the wire are radio-opaque.

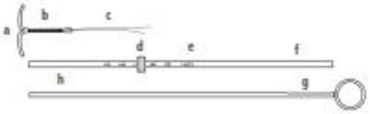

a) side arms
b) vertical arm
c) nylon threads
d) blue flange
e) centimetre scale
f) insertion rod
g) marked part on the plunger
h) plunger

Characteristics

Neo-Safe® T CU 380 / Neo-Safe® T CU 380 Mini IUD offers almost complete protection against pregnancy. Correctly inserted, the IUD is safe for women at low risk of sexually transmitted disease.

The Neo-Safe® T CU 380 / Neo-Safe® T CU 380 Mini is an intrauterine contraceptive device made of polyethylene and barium sulphate. The bended side arms are flexible and shaped in such a way as to keep the IUD adjacent to the fundus, even in case of uterine contractions, without stretching the uterine cavity or touching the entrance of the fallopian tubes.

Neo-Safe® T CU 380 / Neo-Safe® T CU 380 Mini IUD prevents pregnancy by blocking fertilization.

The contraceptive effect is likely due to a pronounced sterile inflammatory reaction, which takes place as a result of a foreign body response in the uterus. The concentration of various types of white blood cells, prostaglandins and enzymes in uterine and tubal fluids increases markedly. These changes interfere with the movement of sperm in the genital tract, reducing their potential fertilizing capacity, so that fertilization is not possible.

In the fallopian tubes, where fertilization is thought to take place, fewer sperm are found in copper IUD users than in non-users. Thus, the primary action is most likely altering the function or survival of sperm and ova before they can meet.

Indication

Female contraception (long lasting).

Contraindications (absolute)

1. Malignant diseases of the genital tract
2. Vaginal bleeding
3. Pregnancy
4. Past history of ectopic pregnancy or predisposing factors
5. Infections of the genital tract
6. Sexually transmitted diseases during the last 12 months (except bacterial vaginitis, repeated herpes infection, hepatitis B)
7. Abortion with infection during the last 3 months, pelvic inflammatory disease (PID)
8. Uterine malformations (congenital or acquired)
9. Allergy to copper

Contraindications (relative)

1. Valvular heart disease
2. Anaemia
3. Coagulation disorders
4. Anti-inflammatory treatment
5. Wilson's disease
6. Multiple sexual partners
7. Nulliparity

Warnings and precautions

Before inserting the IUD, a thorough medical history and an examination of the pelvic and abdominal cavity as well as a cervical smear are mandatory. Pregnancy, genital infections or sexually transmitted diseases should be excluded. The position of the uterus and the size of the uterine cavity must be determined to ensure correct insertion of the IUD. Neo-Safe® T CU 380 is designed for women with a uterine cavity depth of 6 - 9 cm. Neo-Safe® T CU 380 Mini is designed for women with a uterine cavity depth of less than 7 cm.

One month after the insertion of an IUD the woman must be re-examined to determine whether the IUD is properly placed and if there are signs of infection. Subsequent follow-up examinations are to be performed annually or more frequently if clinically indicated.

Pelvic inflammatory disease during IUD use should be treated without delay. For this reason the user must be instructed to report to her physician if there are suspicious signs such as vaginal discharge, pelvic pain and fever. This can be ascertained by gynaecological examination and/or ultrasound (if available). If a treatment with antibiotics is not effective after 48 hours and signs of PID persist, the IUD must be removed immediately.

Excessive bleeding or dysmenorrhoea during the first cycle after insertion should also be carefully assessed to ascertain if this is caused by the IUD, in which case it might have to be removed.

The possibility of perforation of the uterus during the insertion should always be considered, especially if the nylon threads are invisible or cannot be drawn out of the cervical canal. If there are any doubts about the position of the IUD (e.g. if the IUD is extremely difficult or painful to insert) the appropriate diagnostic techniques should be used (flat x-ray of the pelvis, ultrasonography, hysteroscopy, laparoscopy).

If the nylon threads appear to be longer than at insertion, an ultrasound should be carried out to determine if the IUD has been displaced, which might decrease its contraceptive efficacy.

If the threads are not visible at follow-up examination, pregnancy must be excluded. The threads may have been drawn up into the cervical canal or the uterus and usually reappear during the next menstruation. In case of doubt the IUD can be identified by feeling carefully with a suitable instrument, by ultrasound or by an x-ray after excluding pregnancy.

In case of accidental pregnancy with the IUD in situ, it is mandatory to determine (by ultrasound) whether the pregnancy is intrauterine or ectopic. Up to the end of the first trimester, if the threads are visible, the IUD should be carefully removed. After that the patient should be offered the option of elective abortion as soon as possible, since there is an increased risk of PID and many other obstetric problems, e.g. premature labour, placenta praevia and abruptio placentae.

However, if the woman wishes to continue her pregnancy, close monitoring is mandatory. She should be informed about the risks of keeping the IUD in situ. There are no contraindications to breastfeeding with an IUD in situ.

Perforation

Perforation or penetration of the uterine corpus or cervix by the IUD may occur, usually during insertion. The risk is increased during the postpartum period and slightly increased if the woman is fully breastfeeding. The device must be removed as soon as possible if this occurs.

Interactions

The available experience indicates that, in general, drug interference with the contraceptive efficacy of Neo-Safe® T CU 380 / Neo-Safe® T CU 380 Mini is highly unlikely. However, published reports appear to show diminished efficacy with long term use of non-steroidal anti-inflammatory drugs (especially acetyl salicylic acid) and of corticosteroids. Short term use in the treatment of dysmenorrhoea with non-steroidal anti-inflammatory drugs does not appear to reduce contraceptive efficacy.

Do not perform diathermy (short wave and microwave) of the sacral or abdominal region since heating may cause heat injury to the surrounding tissue.

Side effects

During use of IUDs heavier menstrual bleeding, slight intermenstrual bleeding, anaemia, dysmenorrhoea, pain in the lower abdomen or sacral area as well as pelvic infection may occur. The risk of an ectopic pregnancy is increased. In rare cases, the IUD may penetrate into the wall of the uterus or perforate it. Allergic skin reactions may occur.

Physician instructions to IUD users

Users should be informed in detail as to the advantages and disadvantages of IUD contraception, not only so that they understand how it works, but above all so that any complications can be detected early.

The user must learn how to feel the threads emerging from the cervical canal.

Insertion

First eliminate the possibility of pregnancy!

The best time for insertion is during menstruation to prevent insertion during non-diagnosed pregnancy. At this time the external and internal cervical os are physiologically dilated. This facilitates the insertion of the IUD without the need to dilate the canal in most instances.

Alternatively, the IUD may be introduced within 5 days of unprotected coitus or within 15 minutes of delivery of the placenta or abortion in the first trimester. In these last two cases a higher expulsion rate is expected. Insertion immediately after unprotected coitus can increase the risk of PID.

If the IUD cannot be inserted immediately after delivery of the placenta or abortion, insertion should be delayed for at least six weeks. In case of caesarean section insertion should be delayed for 12 weeks after delivery, although this is controversial.

Prior to insertion the vagina, cervix and cervical canal should be cleansed with an antiseptic solution. It is essential to determine the exact position of the uterus by bimanual pelvic palpation so that the Neo-Safe® T CU 380 / Neo-Safe® T CU 380 Mini can be inserted along its longitudinal axis. This can be accomplished by grasping the anterior or posterior lip of the cervix, depending on whether the uterus is anteverted or retroverted.

A local anaesthetic may be applied as a gel or can be injected in and around the cervix prior to insertion.
Hysterometry should be carried out and the ascertained depth of the uterus be marked on the Neo-Safe® T CU 380 / Neo-Safe® T CU 380 Mini inserter tube with the aid of the movable flange.

Step-by-Step loading instructions
The IUD may be used by trained medical staff only. In order to minimize the risk of contamination we recommend using sterile gloves.

1. Open the sterile packaging of the Neo-Safe® T CU 380 / Neo-Safe® T CU 380 Mini halfway. Grasp the nylon threads and pull the device gently into the insertion tube (fig. 1) until the knobs at the end of the side arms cover the opening of the tube. The knobs should not be pulled into the tube.

1

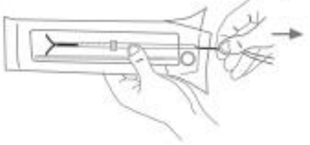

Note: The ends of the side arms of the Neo-Safe® T CU 380 / Neo-Safe® T CU 380 Mini must not remain bended for over five minutes within the insertion tube; otherwise they may not bend back.

2. Steadying the blue flange with one hand, pull the insertion tube until the lower rim of the flange indicates the previously sounded length of the uterus on the scale (fig. 2).

2

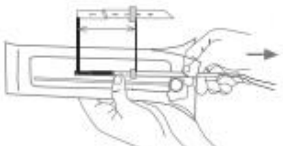

Holding the threads straight in the tube, place the plunger into the insertion tube. This prevents that the threads will be disarranged by inserting the plunger.
Prior to insertion the tube can be bent to conform to the position of the uterus. The bending must be performed whilst the device remains in the sterile package after placing the plunger into the insertion tube.

3. Ensure that the blue flange indicates the direction in which the side arms of the IUD will open in the uterus.
Remove the loaded insertion tube from the package and insert it through the cervical canal into the uterus until the blue flange touches the cervical os (fig. 3).

3

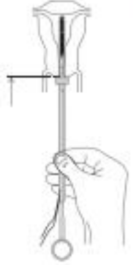

4. Hold the plunger tight and pull the insertion tube downwards until the lower rim of the tube reaches the marked part of the plunger. The horizontal arms of the IUD are now released in the uterus (fig. 4).
Observe that the distance between the flange and the cervical os is now about 1,5 cm.

4

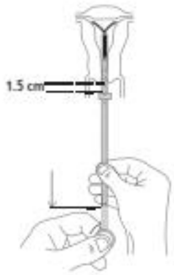

5. Holding the tube and the plunger together, gently push both as far as the blue flange again touches the cervical os (fig. 5). The IUD is now in the correct position.

5

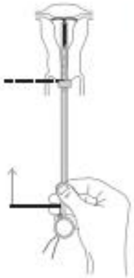

6. Holding the plunger steady, pull the tube down to the ring of the plunger. The IUD is now entirely released in the uterus (fig. 6).
To prevent pulling the device from the fundal position, first remove the plunger while keeping the insertion tube steady and only then remove the insertion tube.

6

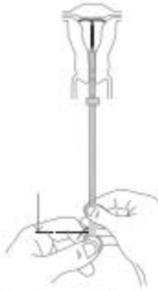

Cut the threads leaving 2-3 cm visible outside the cervix.

Removal
Neo-Safe® T CU 380 / Neo-Safe® T CU 380 Mini should be replaced after 5 years.

Again, the most appropriate time to remove the IUD is during menstruation since both the internal and external cervical os are fully dilated. Grasp the threads of the IUD with a tenaculum and pull it along the longitudinal axis of the uterus. Try to insert the forceps at the entrance to the cervical canal in order to grasp the vertical arm of the IUD as soon as it has passed the internal cervical os. This avoids excessive tension on the threads, which could cause them to break. While doing this, distract the patient's attention by asking her to cough, and then remove the Neo-Safe® T CU 380 / Neo-Safe® T CU 380 Mini with one firm tug.
Insertion and removal of the IUD could cause slight pain and bleeding or may precipitate a vasovagal attack or an epileptic seizure.
After removal, inspect the Neo-Safe® T CU 380 / Neo-Safe® T CU 380 Mini to see that none of its arms have been left in the uterine cavity.

In case of a lost IUD or lost parts of an IUD in the uterine cavity either hysteroscopy or ultrasound or x-rays should be used to determine its location: curettage may be advisable. In very rare cases of uterine perforation laparoscopy may be needed.

Packaging
1 x 1 sterile Neo-Safe® T CU 380 / Neo-Safe® T CU 380 Mini
10 x 1 sterile Neo-Safe® T CU 380 / Neo-Safe® T CU 380 Mini

Pharmaceutical information
Each IUD is sterilized with ethylene oxide and is intended for single use only. Do not resterilize. Do not use if package is damaged or open. Do not use after the expiry date printed on the sterile package. After removal the Neo-Safe® T CU 380 / Neo-Safe® T CU 380 Mini is to be disposed of in accordance with the guidelines in force for dealing with potentially infectious material.

Shelf-life: Four years.

Storage: Store in a dry place, below 35°C. Protect from direct sunlight and moisture.

Content: Copper, Polyethylene, Barium Sulphate, Polyamide 6.

Incompatibilities: No known incompatibilities.

Nature and contents of container: The device with accessories has been packed in a heat sealed sterilised pouch made of Tyvek + PET/PE.

Date of first authorization: 25.05.2003
Date of last renewal: 31.07.2014

Date of revision of the text: 11.2014

Sterile

Neo-*Safe*® T CU 380
Neo-*Safe*® T CU 380 Mini

CE
0120

Manufactured
Mona Lisa N.V.
Graaf de Theuxlaan 25, bus 2
3550 Heusden-Zolder
Belgium

(EN)

Neo-*Safe*® T CU 380

INFORMATION FOR THE USER

Design and dimensions

The Neo-Safe® T CU 380 Intrauterine Device (IUD) is made of a T-shaped frame of polyethylene and barium sulphate. The vertical arm is wound with copper wire. The copper surface is 380 mm². Two nylon threads are attached to the lower end of the vertical arm. Dimension: 31,8 mm ± 0,2 mm wide, 31,9 mm ± 0,2 mm long.
The polyethylene frame and the wire are radio-opaque.

Mode of action and possible effects

There are different types of intrauterine contraceptive devices. Wide-ranging international studies still verify that Copper-IUDs are not only safe but also effective over long periods. In addition, it is also generally recognized that Neo-Safe® T CU 380 IUDs are one of the most effective means of contraception. Neo-Safe® T CU 380 is shaped in a way to prevent irritation of the uterus. It is usually inserted during menstruation. The insertion is normally painless. The opening of the uterus is examined, the length of the uterine cavity is determined and the Neo-Safe® T CU 380 is carefully inserted into the uterine cavity, until it is in the correct position and touches the fundus. As soon as the Neo-Safe® T CU 380 has been inserted, you are protected against pregnancy.

Neo-Safe® T CU 380 is effective for five years.
IUDs are highly effective and can only be compared with the oral contraceptive pill for the prevention of pregnancy. No contraceptive method offers 100% safety. However, in practical terms, with Neo-Safe® T CU 380 you are protected.

It is not fully known how IUDs work. However, nowadays it is assumed that the most probable effect is that it disrupts the normal function of the male gametes (sperm), which become incapable of fertilizing the female egg. It is also assumed that copper ions influence the development of the egg so that fertilization does not take place: IUDs are no longer considered a method of abortion.

Contraindications

Neo-Safe® T CU 380 should not be used in case of:

1. Malignant diseases of the genital tract
2. Vaginal bleeding
3. Pregnancy
4. Past history of ectopic pregnancy or predisposing factors
5. Infections of the genital tract
6. Sexually transmitted diseases during the last 12 months (except bacterial vaginitis, repeated herpes infection, hepatitis B)
7. Abortion with infection during the last 3 months, pelvic inflammatory disease (PID)
8. Uterine malformations (congenital or acquired)
9. Allergy to copper

The application should be reconsidered at:

1. Valvular heart disease
2. Anaemia
3. Coagulation disorders
4. Anti-inflammatory treatment
5. Wilson's disease
6. Multiple sexual partners
7. Nulliparity

Possible complications and precautions

The first three cycles following insertion are usually different from your normal cycles. Periods can be heavier, cramps and spotting may occur both before and afterwards. Three to four cycles after insertion of the IUD menstruation normally resumes as before insertion.
Sometimes these changes are experienced between the menstrual periods during the cycle.
You should contact your doctor if:

- you can not feel the threads of the IUD,
- you or your partner are able to feel the lower end of the IUD,
- you think you are pregnant,
- you experience abdominal pain, fever or unusual vaginal discharge,
- you or your partner feel pain or discomfort during sexual intercourse,
- your menstruation is delayed, since rarely a pregnancy outside the uterine cavity, e.g. in a fallopian tube (ectopic pregnancy) may occur,
- you wish to have the IUD removed and / or want to become pregnant.

Insertion and removal

Before the insertion, you must be informed on the efficacy, risks and side effects of Neo-Safe® T CU 380. A gynaecological examination including pelvic examination and a cervical smear should be performed. Pregnancy, genital infections and sexually transmitted diseases should be excluded. The position of the uterus and the size of the uterine cavity should be determined.

The instructions for insertion should be carefully taken into account and the patient card be filled out together with the attending doctor. You should return for check-up 4 to 12 weeks after insertion and once a year thereafter, or more frequently if clinically indicated.

Insertion is recommended during or shortly following menstruation. If pregnancy is excluded, Neo-Safe® T CU 380 may be inserted at any time of the cycle. It can be also inserted immediately after first trimester abortion. After having a baby, insertion should be postponed until six weeks after delivery, as this is associated with greater rates of perforation and expulsion. There are no problems about breast feeding with an IUD.

Neo-Safe® T CU 380 is removed by gently pulling on the threads. If the threads are not visible, and the device is in the uterine cavity, removal should be postponed until after the next menstrual bleeding since the threads usually become visible immediately after menstruation. If they are still not visible, the device may be removed using a narrow tenaculum. This may require dilatation of the cervical canal. After removal, pregnancy is immediately possible.
If you wish to continue using the method, a new device can be inserted at the same time. If pregnancy is not desired, the removal should be carried out during the menstruation.

Insertion and removal may be associated with some pain and bleedings. Occasionally, the procedure may precipitate fainting or a seizure, if you suffer from epilepsy.

It is recommended that you visit your doctor for a check-up one month after Neo-Safe® T CU 380 was inserted to make sure the IUD is still in your uterus and is positioned correctly. An annual visit is recommended thereafter. You should also check to make sure that Neo-Safe® T CU 380 is still in your uterus by reaching up to the top of your vagina, while in a squatting position, with clean fingers to feel the threads. Do not pull on the threads.
If you cannot feel the threads or you can feel more of the Neo-Safe® T CU 380 than just the threads, consult your doctor immediately. This suggests that your Neo-Safe® T CU 380 is not in the uterus or is incorrectly positioned which would increase your chances of getting pregnant. It is recommended that you use an additional birth control method until you have seen your doctor. It is good practice to check that Neo-Safe® T CU 380 is in place once a month.

Interactions

The available experience indicates that, in general, drugs do not interfere with the contraceptive efficacy of Neo-Safe® T CU 380 IUDs. However, there are published reports which appear to show diminished efficacy with long-term use of non-steroidal anti-inflammatory drugs (especially acetylsalicylic acid) and corticosteroids. Short-term use in the treatment of dysmenorrhoea with non-steroidal anti-inflammatory drugs does not appear to reduce contraceptive efficacy.

Side effects

In rare cases pain or dizziness may occur after insertion. If these complaints don't abate within half an hour of resting, the position of the IUD should be checked and the IUD may have to be removed. The IUD may rarely cause allergic skin reactions.

Patient card

Please complete carefully together with your physician and keep in a safe place.

The IUD is sterile and is intended for one-off use. Do not use it more than once.

Date of revision of the text: 11.2013

Sterile

Neo-*Safe*® T
CU 380

CE
0120

Manufacturer:
Mona Lisa N.V.
Graaf de Theuxlaan 25, bus 2
3550 Heusden-Zolder
Belgium
