# Supplementary material for: Continuation rates of two different-sized copper intrauterine devices among nulliparous women: Interim 12-month results of a single-blind, randomised, multicentre trial
Source: eClinicalMedicine. 2022 Jul 16;51:101554. doi: 10.1016/j.eclinm.2022.101554 (PMC9294241; doi:10.1016/j.eclinm.2022.101554)
Supplement: Supplementary file 2 [file mmc2.pdf]

This document contains two supplementary files

1. **Inclusion/Exclusion Criteria**
2. **Adverse event table**

Inclusion/Exclusion Criteria

Inclusion Criteria

- 16-40 years
  - 16 and 17 year olds, where permissible by state regulations and local Institutional Review Board (IRB) approval
- Sexually active, anticipating at least one act of vaginal intercourse per menstrual cycle with a male partner and at risk for pregnancy
- seeking contraception, and willing to use the study IUD as the only contraception method
- willing to be randomized to one of the two copper IUDs
- has an intact uterus and at least one ovary
- has a history of regular menstrual cycles; defined as occurring every 21-35 days when not using hormones, and with a variation of typical cycle length of no more than 5 days
- able and willing to provide written informed consent
- agrees to follow all study requirements
- not currently pregnant or at risk for luteal phase pregnancy based on history of unprotected intercourse

Exclusion Criteria

- abnormal Pap requiring treatment after enrollment
- known human immunodeficiency virus / acquired immunodeficiency syndrome (HIV/AIDS) infection
- intending to become pregnant in the 37 months after enrollment
- known infertility
- history of allergy or sensitivity to copper
- previous tubal sterilization
- has received an injectable contraceptive in the last 9 months and has not resumed regular menstrual cycles (as evidenced by 2 spontaneous menses)
- within 30 days of administration of mifepristone and/or misoprostol for medical abortion or for miscarriage management
- within 30 days of first, second, or third trimester abortion or miscarriage (note: potential abortion/miscarriage participants can be screened and return after 30 days for randomization and IUD insertion)
- within 30 days of delivery (for parous population)
- breastfeeding or recently breastfeeding women unless two consecutive normal menstrual periods have occurred after delivery and prior to enrollment.
- wants to use a copper IUD for emergency contraception
- has previously participated in the study
- participated in another clinical trial involving an investigational product within the last 30 days (before screening) or planning to participate in another clinical trial involving an intervention or treatment during this study
- not living in the catchment area of the study site or planning to move from the area within the year (unless known to be moving to the catchment area of another study site)
- known or suspected current alcohol or drug abuse
- planning to undergo major surgery during study participation
- current need for use of exogenous hormones or therapeutic anticoagulants (Note: subjects who start a therapeutic anticoagulant after enrolment will be allowed to continue in the study.)

- at high risk for sexually-transmitted infections or pelvic infection
- anticipated need for regular condom use (refer to Section 8.1).
- has any condition (social or medical) which in the opinion of the Investigator would make study participation unsafe or complicate data interpretation
- Reported medical contraindications (Medical Eligibility Criteria category 3 or 4)[14] to copper IUDs, including:
    - suspicious unexplained vaginal bleeding
    - known cervical cancer
    - known endometrial cancer
    - known Wilson's disease
    - Confirmed gestational trophoblastic disease with persistently elevated beta-hCG levels or malignant disease, with evidence or suspicion of intrauterine disease
    - anatomic abnormalities with distorted uterine cavity
    - current pelvic inflammatory disease (PID)
    - pelvic tuberculosis
    - immediately post-septic abortion or puerperal sepsis
    - current known purulent cervicitis or chlamydial infection or gonorrhea; Note: to enroll, there must be no obvious signs of infection at the time of enrollment based on pelvic exam. If lab results come back for positive infection after enrollment, treatment should be provided but the IUD can be left in place.
    - complicated solid organ transplantation
    - systemic lupus erythematosus with severe thrombocytopenia

Allowable Criteria

- has been using oral contraceptives, vaginal ring, or patch and is discontinuing the product to be enrolled
- has been using a contraceptive implant or IUD, but wishes to have the device removed prior to enrollment

Adverse Events

Safety analysis of adverse events (AEs) and serious adverse events (SAEs) in first 12 months			
	**NT380-Mini (N=744)**	**TCu380A (N=183)**	**p-value***
Number of AEs	3496	802	
Number of participants with AEs	697 (93·7%)	163 (89·1%)	0·031
Number of related AEs	2195	506	
Number of participants with any related AEs	642 (86·3%)	150 (82·0%)	0·137
Number of AEs leading to study discontinuation	107	40	
Number of participants with AEs leading to study discontinuation	106 (14·2%)	40 (21·9%)	0·011
Number of SAEs	14	0	
Number of participants with SAEs	14 (1·9%)	0 (0·0%)	0·085
Enteritis	1	0	
Food poisoning	1	0	
Oesophageal achalasia	1	0	
Oesophagitis	1	0	
Suicidal ideation	2	0	
Depression	1	0	
Suicide attempt	1	0	
Non-cardiac chest pain	1	0	
Anaphylactic reaction	1	0	
Pyelonephritis	1	0	
Open fracture	1	0	
Diabetic ketoacidosis	1	0	
Thyroid cancer	1	0	

Values are n (%).

* chi-square test or Fisher's exact test
